# Supplementary material for: Efficient human-like antibody repertoire and hybridoma production in trans-chromosomic mice carrying megabase-sized human immunoglobulin loci
Source: Nat Commun. 2022 Apr 5;13:1841. doi: 10.1038/s41467-022-29421-2 (PMC8983744; doi:10.1038/s41467-022-29421-2)
Supplement: Supplementary file 1 — Supplementary Information [file 41467_2022_29421_MOESM1_ESM.pdf]

Efficient human-like antibody repertoire and hybridoma production in trans-chromosomic mice carrying megabase-sized human immunoglobulin loci

First author : Hiroyuki Satofuka, Satoshi Abe, and Takashi Moriwaki
Corresponding author : Yasuhiro Kazuki

Supplementary Information

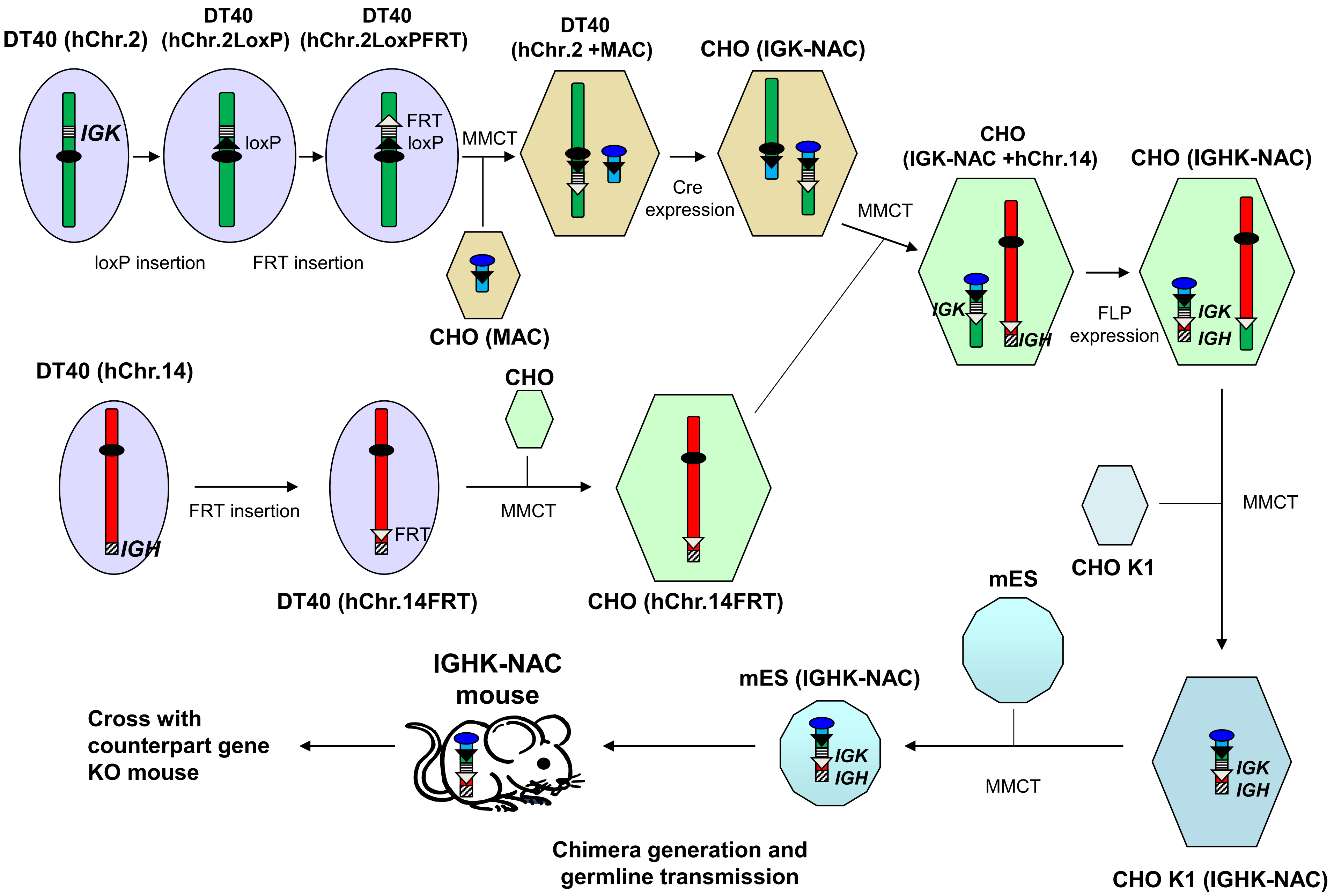

Supplementary Figure 1| Schematic depiction of the generation of fully human Ab-producing mice via chromosome engineering.
Chromosome modifications were performed in chicken DT40 cells carrying the entire hChr.2 *IGK* locus or hChr.14 *IGH* locus. Following IGK-NAC construction and its transfer into CHO cells carrying a hChr.14, the IGHK-NAC was constructed. The IGHK-NAC was transferred to mES cells through CHO K1 cells to produce the chimeric mice. Mice carrying the IGHK-NAC were mated with endogenous Ig-gene knockout mice to produce fully human Ab-producing mice.

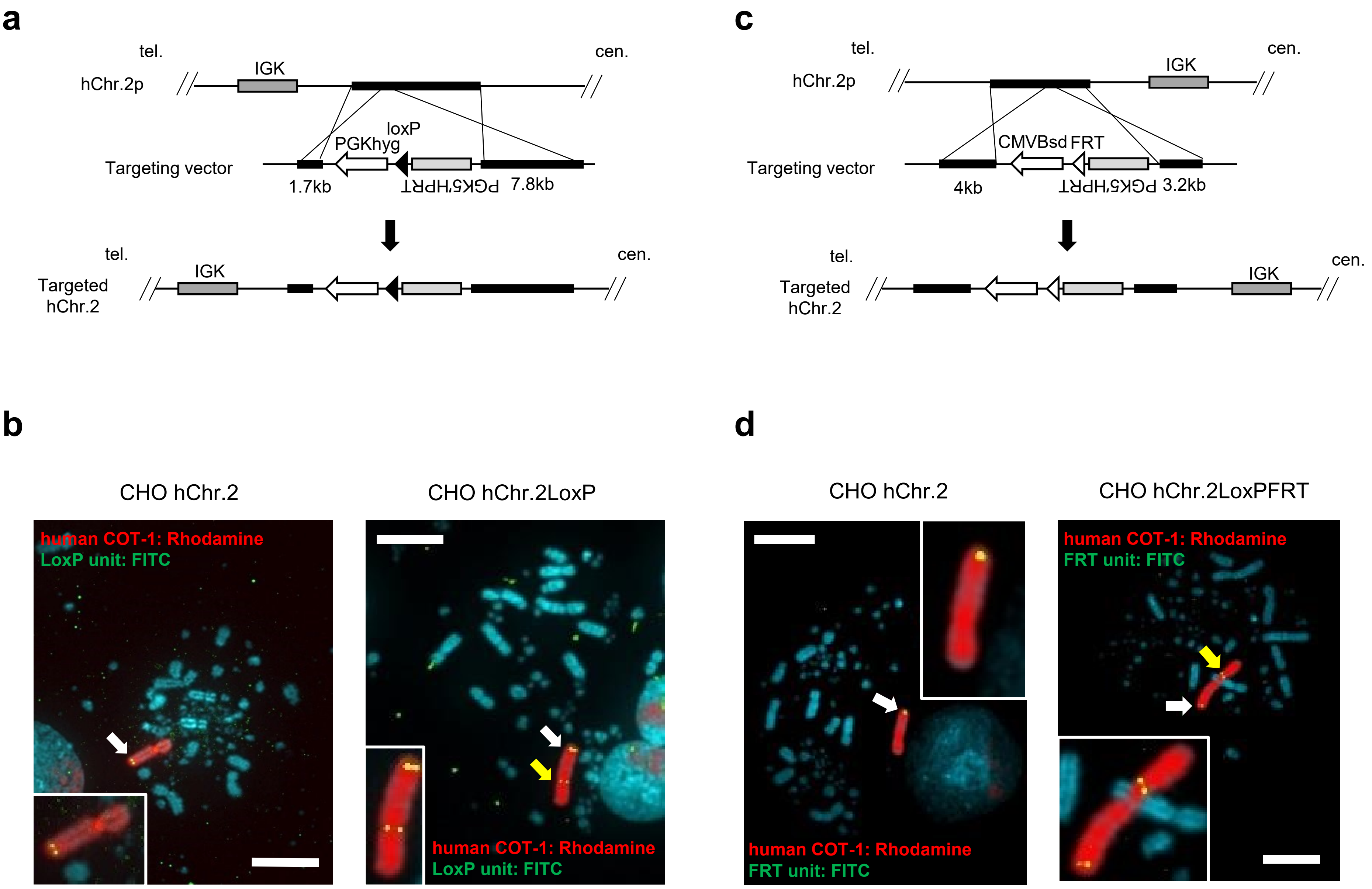

Supplementary Figure 2| Modification of hChr.2 in chicken DT40 cells.
(a) Schematic representation of the targeted integration of the loxP site into the proximal side of the IGK locus on hChr.2p. **(b)** FISH analysis detecting the loxP unit integrated into hChr.2 in DT40 cells carrying an intact hChr.2 (Left) and hChr.2LoxP (Right). Red indicates hChr.2. White and yellow arrows indicate the neomycin resistance marker and the loxP unit, respectively. Insets show enlarged images. **(c)** Strategy for the targeted integration of the FRT site into hChr.2. **(d)** FISH analysis detecting the FRT unit integrated into hChr.2 in DT40 cells carrying hChr.2loxP (Left) and DT40 cells carrying hChr.2LoxPFRT (Right). Red indicates hChr.2. White and yellow arrows indicate the neomycin resistance marker and the FRT unit, respectively. Insets show enlarged images. Scale bar (10 μm).

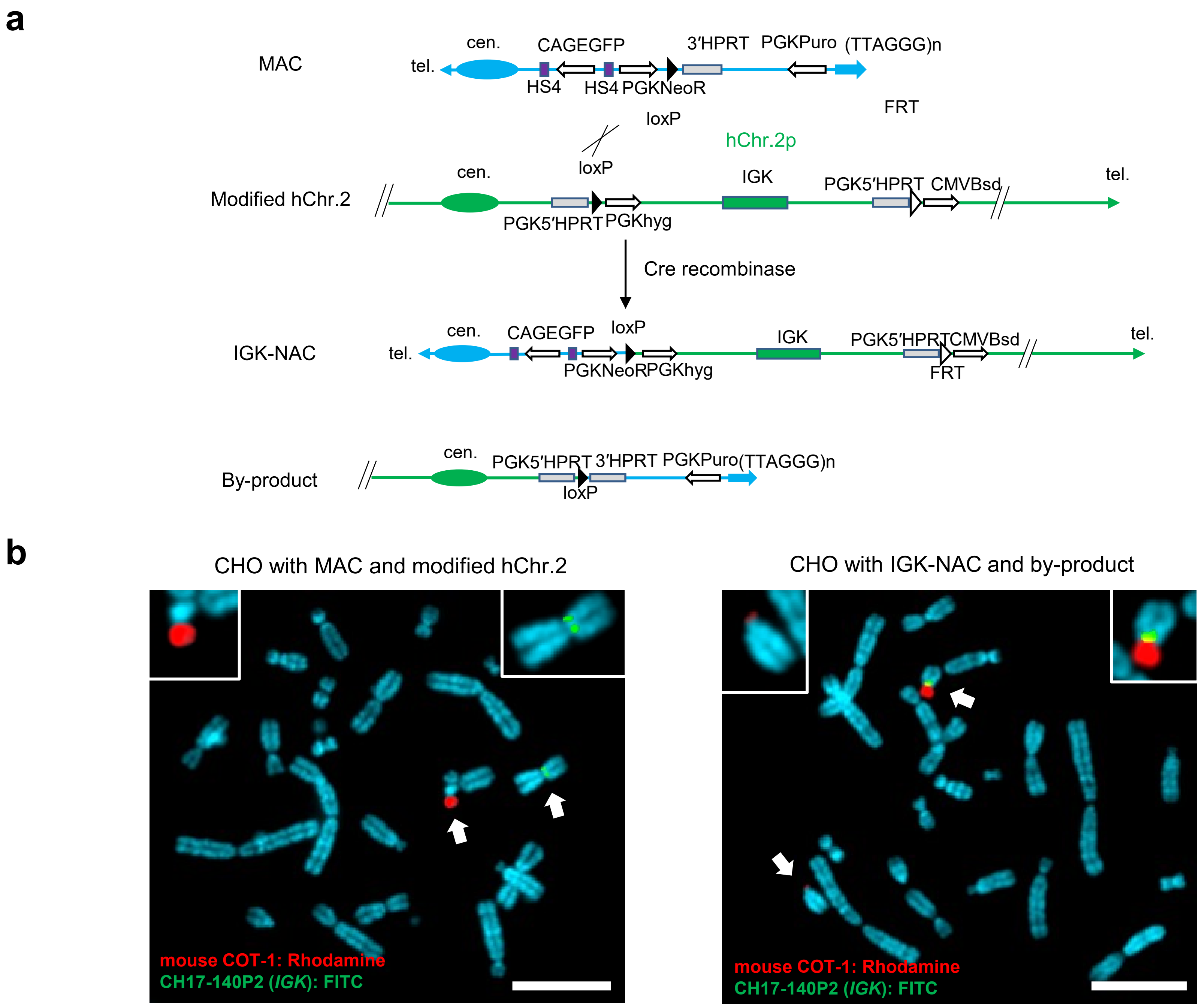

Supplementary Figure 3| Construction of IGK-NAC in CHO cells via Cre/loxP recombination.
(a) Strategy for the generation of IGK-NAC via Cre/loxP recombination-mediated translocation. Recombination between the MAC and the modified hChr.2 generates the IGK-NAC and a by-product. **(b)** FISH analysis confirmed the generation of the IGK-NAC in CHO cells carrying the MAC and modified hChr.2 (Left) and CHO cells carrying the IGK-NAC and by-product (Right). Red and green indicate the MAC and hChr.2, respectively. Insets show enlarged images. Scale bar (10 μm).

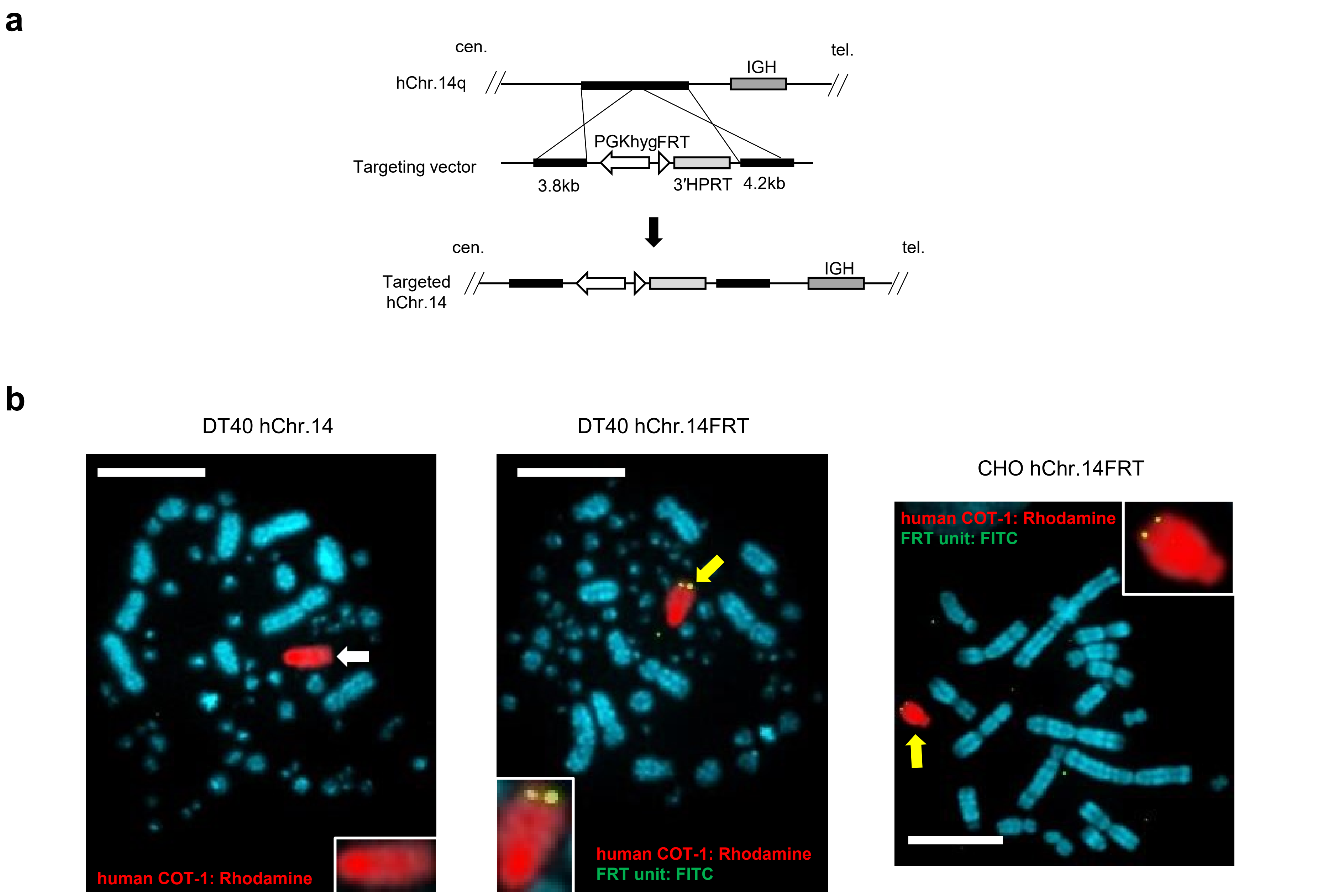

Supplementary Figure 4| Modification of hChr.14 in DT40 cells and transfer of modified hChr.14 to CHO cells. **(a)** Strategy for the targeted integration of the FRT site into hChr.14. **(b)** FISH analysis detected targeted integration of the FRT unit into hChr.14 and transfer of hChr.14FRT to CHO cells. DT40 with intact hChr.14 (left), DT40 with hChr.14FRT (middle) and CHO with hChr.14FRT (right). Red and green indicate hChr.14 and FRT units, respectively. Insets show enlarged images. Scale bar (10μm).

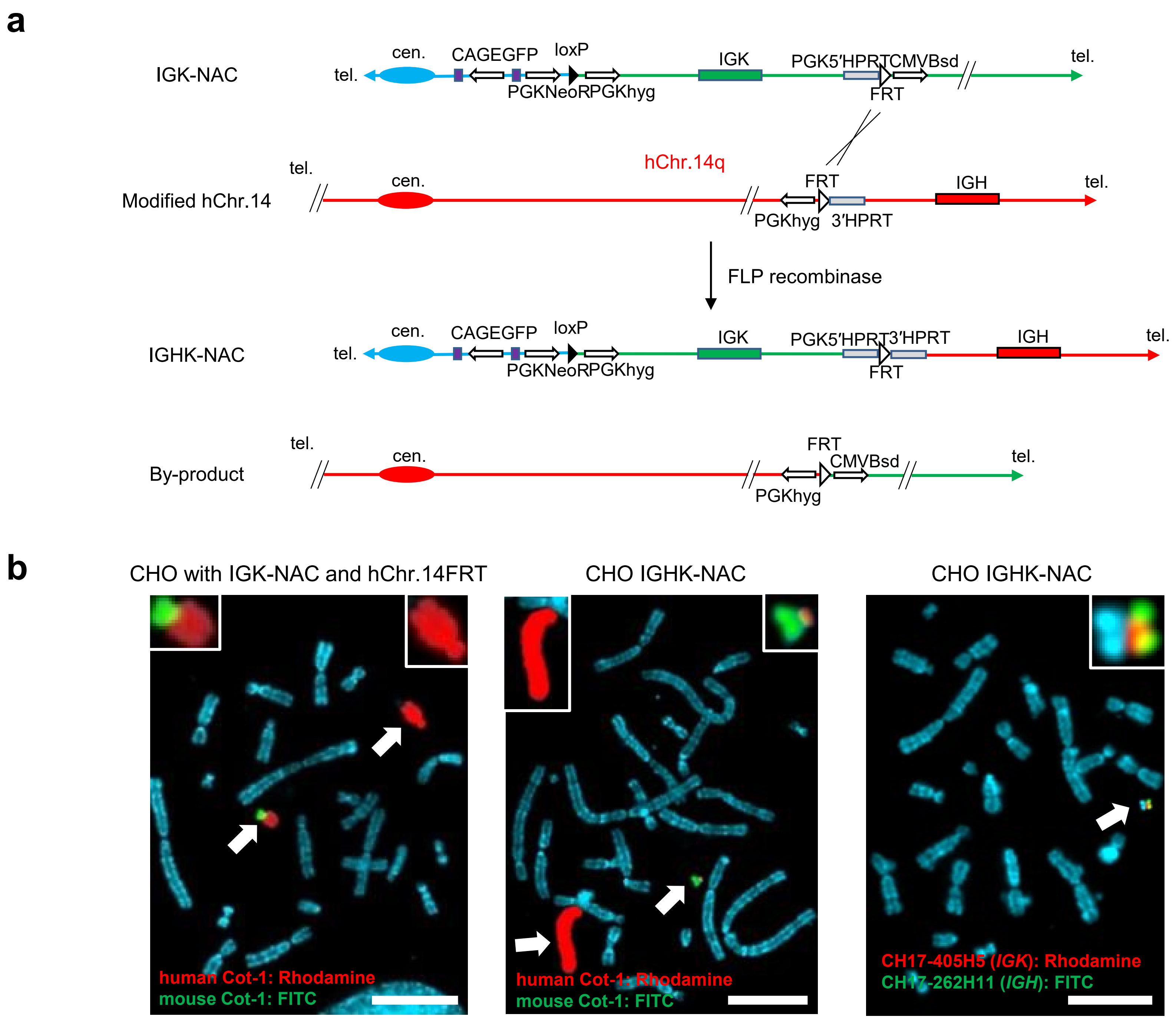

Supplementary Figure 5| Construction of IGHK-NAC in CHO cells via Flp/FRT recombination.
(a) Strategy for the generation of IGHK-NAC via Flp/FRT recombination-mediated translocation. Recombination between IGK-NAC and modified hChr.14 generates IGHK-NAC and a by-product. **(b)** FISH analysis confirmed the generation of IGHK-NAC in CHO cells carrying the IGK-NAC and modified hChr.14 (Left) and CHO cells carrying the IGHK-NAC and by-product (Middle and right). Red and green indicate human genome and MAC, respectively (Left and middle). Red and green indicate parts of the *IGK* and *IGH* regions, respectively (Right). Insets show enlarged images. Scale Bar (10μm).

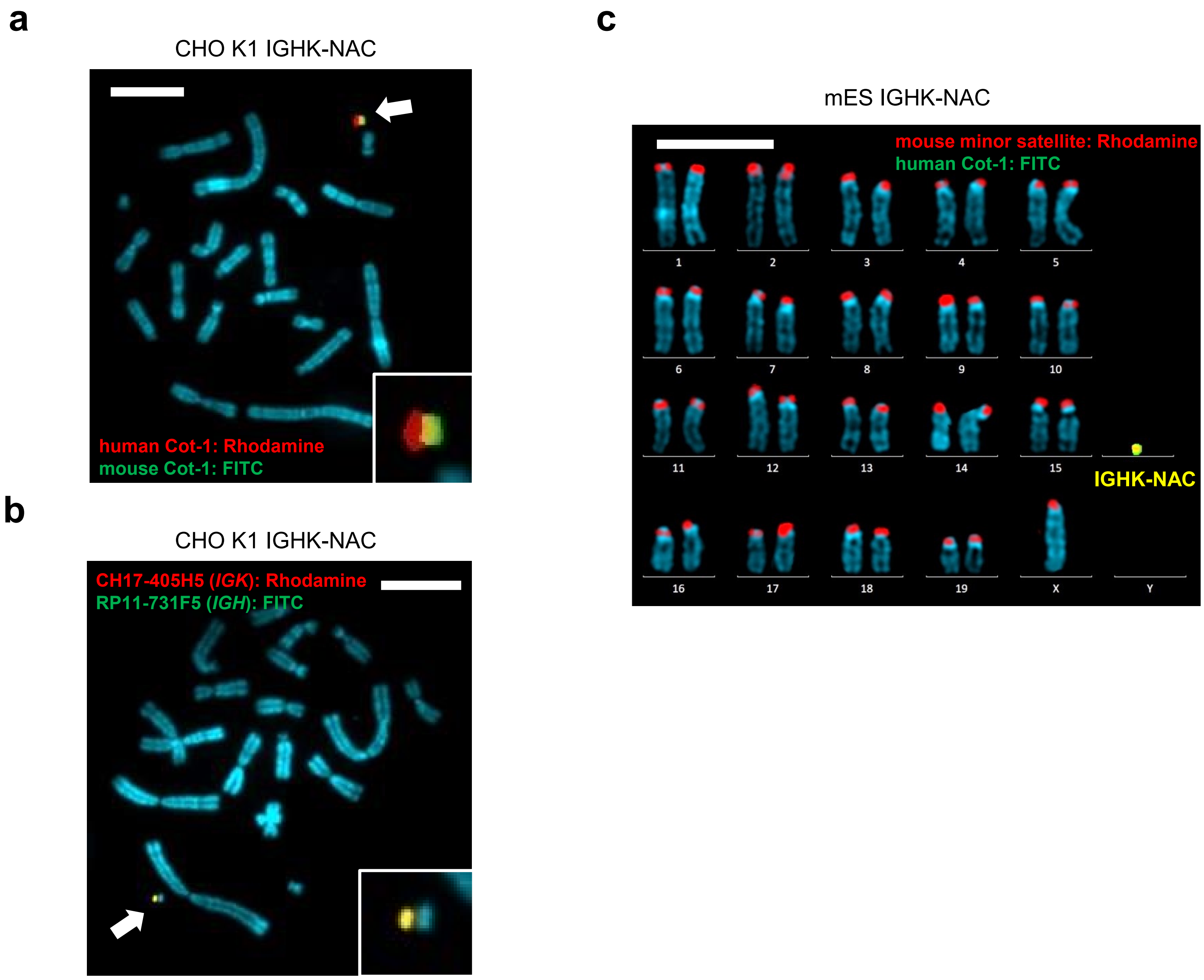

Supplementary Figure 6| Transfer of the IGHK-NAC to mES cells through CHO K1 cells.
(a) FISH analysis of CHO K1 carrying the IGHK-NAC. Green and red indicate the MAC and human genome, respectively. **(b)** FISH analysis of CHO K1 carrying the IGHK-NAC. Red and green indicate parts of the *IGK* and *IGH* regions, respectively. **(c)** Representative karyotype image obtained by FISH analysis of mES cells carrying the IGHK-NAC. Red and green indicate the mouse minor satellite and the human genome, respectively. Scale bar (10 μm).

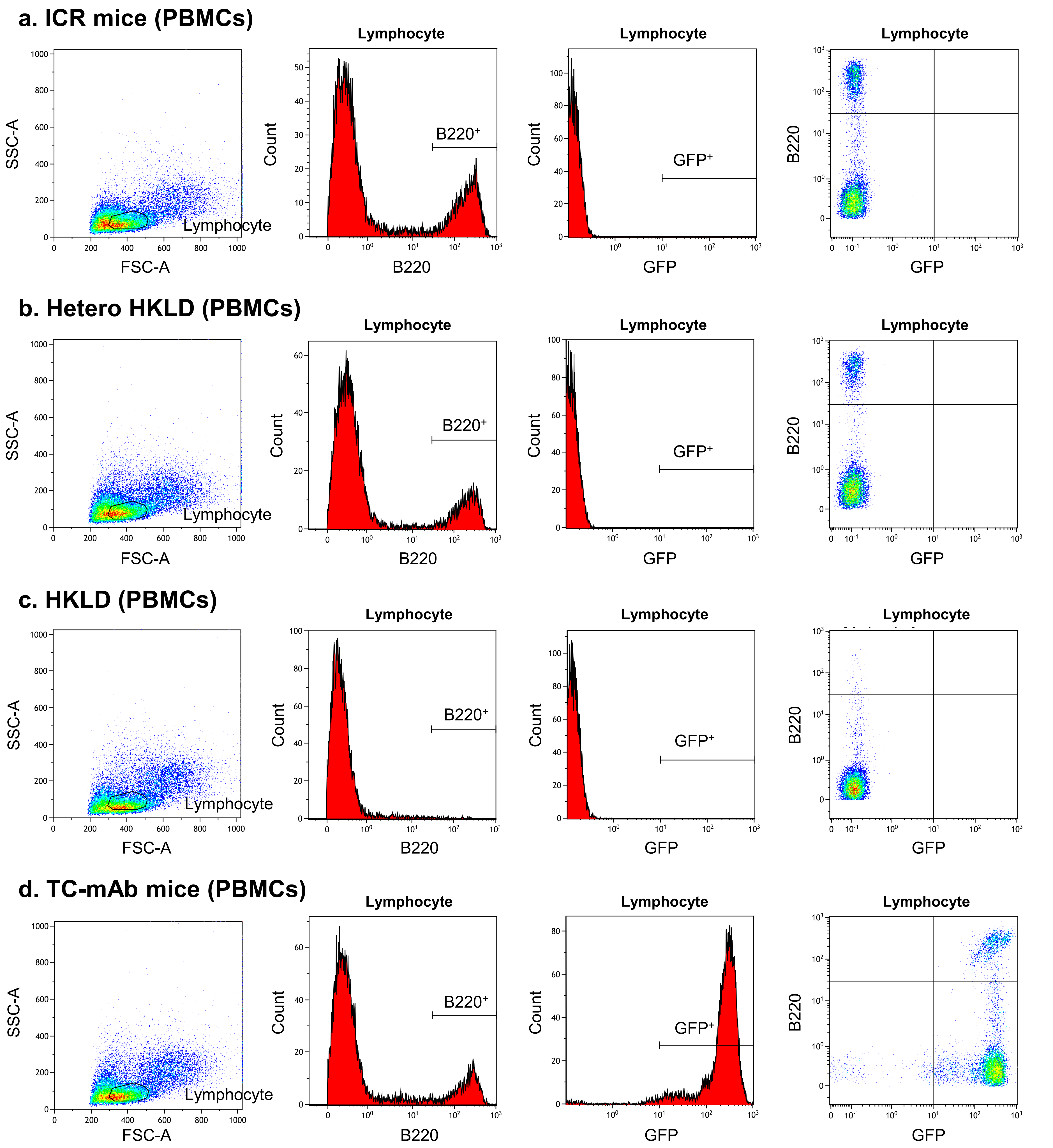

Supplementary Figure 7| Flow cytometry identification of MAC-carrying B cells in peripheral blood mononuclear cells. Flow cytometry gating strategies for MAC-containing cells (GFP^{+}) and B cells ($B220^{+}$) in peripheral blood mononuclear cells (PBMCs) of ICR **(a)** , hetero HKLD **(b)**, HKLD **(b)**, and TC-mAb mice **(d)**. The gating strategies of GFP expressing cells and $B220^{+}$ B cells were used to collect the statistical data presented on Figure 1e and f, respectively. $B220^{+}$ cells were detected in the GFP^{-} lymphocyte subset in ICR, Hetero HKLD, and HKLD mice, and in the GFP^{+} lymphocyte subset in TC-mAb mice.

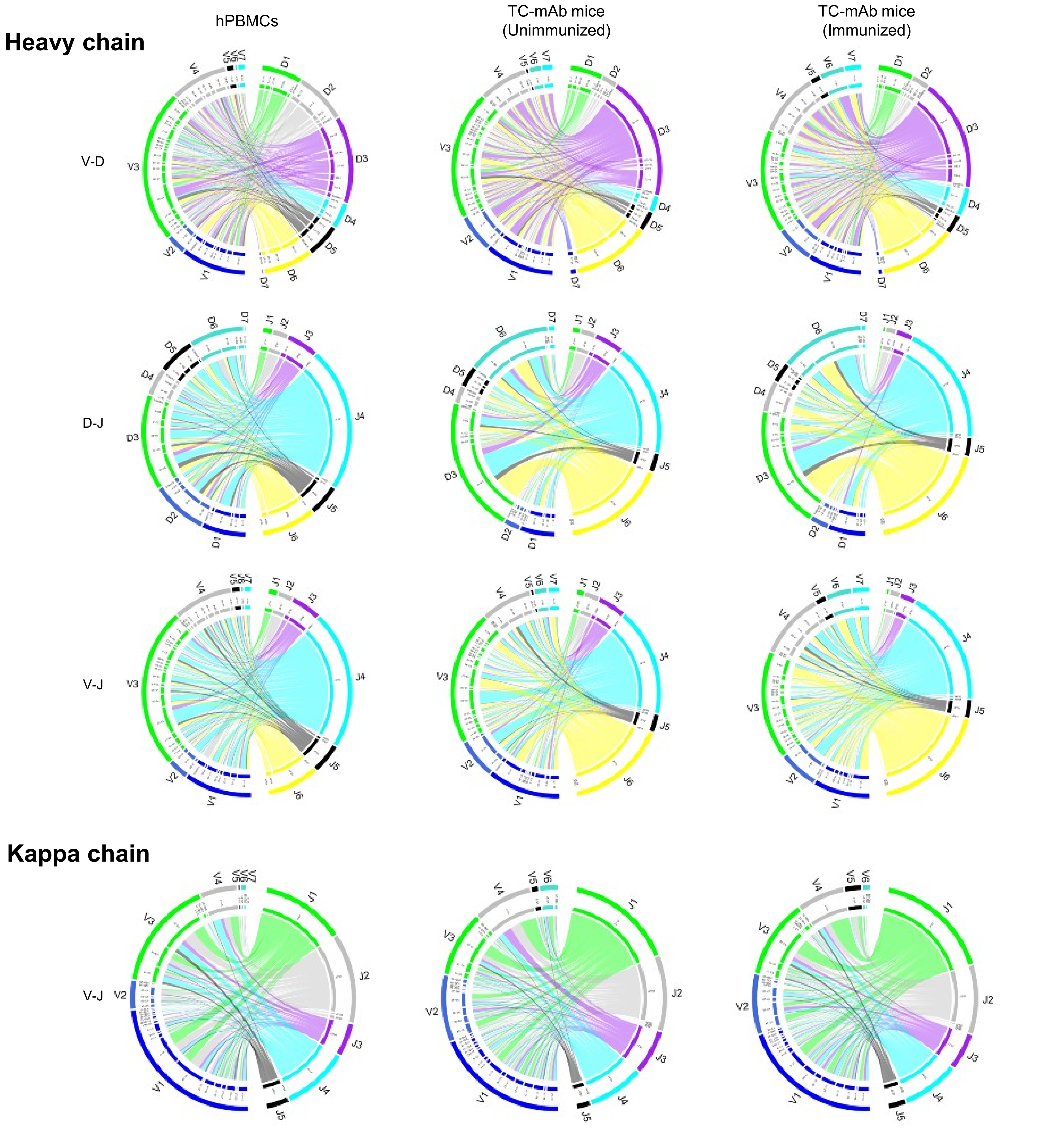

Supplementary Figure 8| Comparison of V(D)J gene association in TC-mAb mice.
The circos plots were represented to compare of V(D)J genes detailed association. Outermost tracks mark the boundaries of each V, D, or J region subfamily in the circos plot. Internal tracks indicate the relative frequencies of subgroups within subfamilies. Highlighted links indicate combinations that constitute 1% or more of all sequences observed. Links indicate the relative frequencies of specific V-D, D-J, and V-J combinations of heavy chain and V-D combinations of light chain. The wider links indicate higher frequencies of recombination. The circos plots of hPBMCs, unimmunized TC-mAb mice, and OVA-immunized TC-mAb mice were represented.

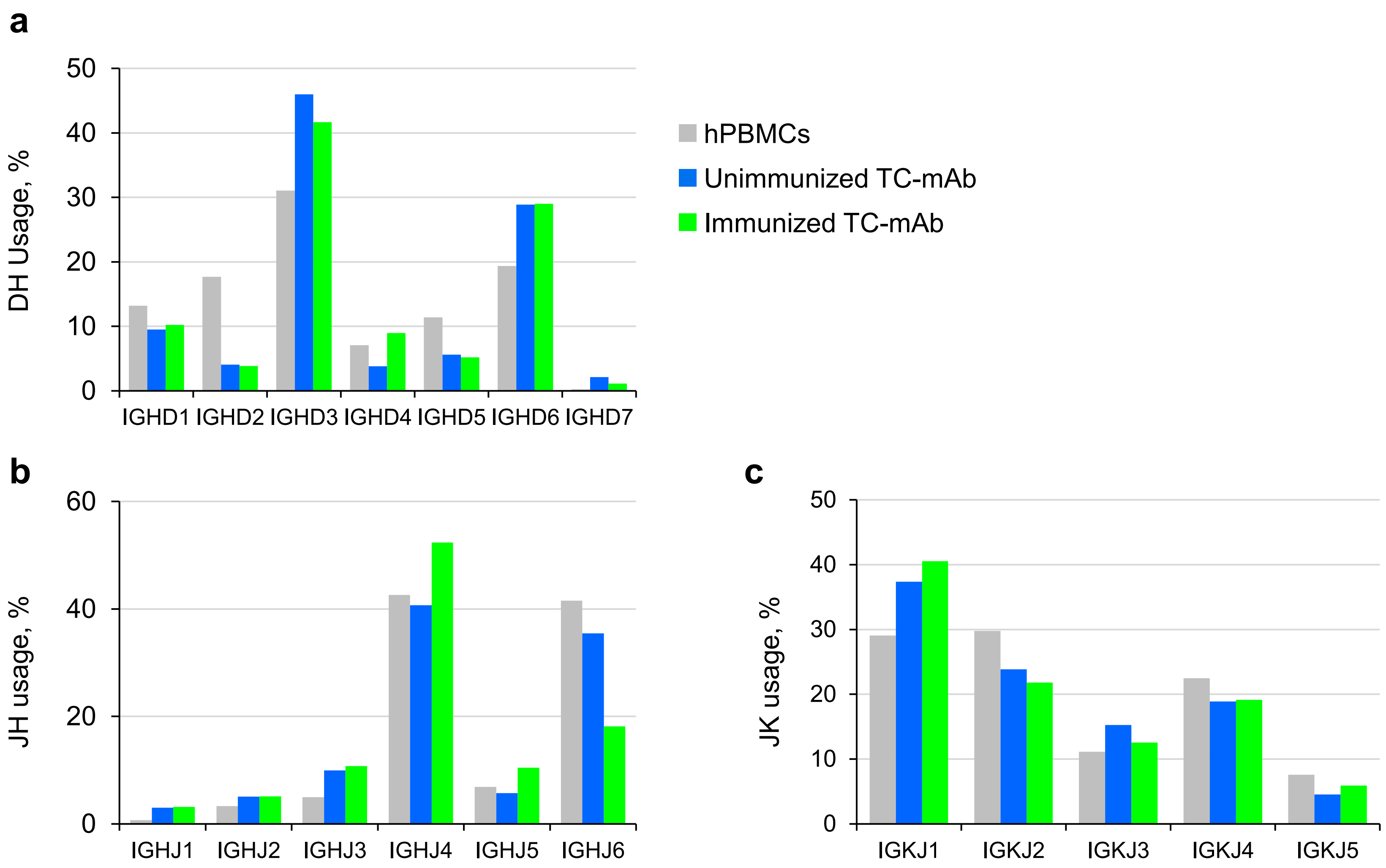

Supplementary Figure 9| Comparison with the DJ usage between hPBMCs and TC-mAb mice.
The frequency use of DH **(a)**, JH **(b)**, and JK **(c)** were compared between hPBMCs, unimmunized TC-mAb mice, and OVA-immunized TC-mAb mice.

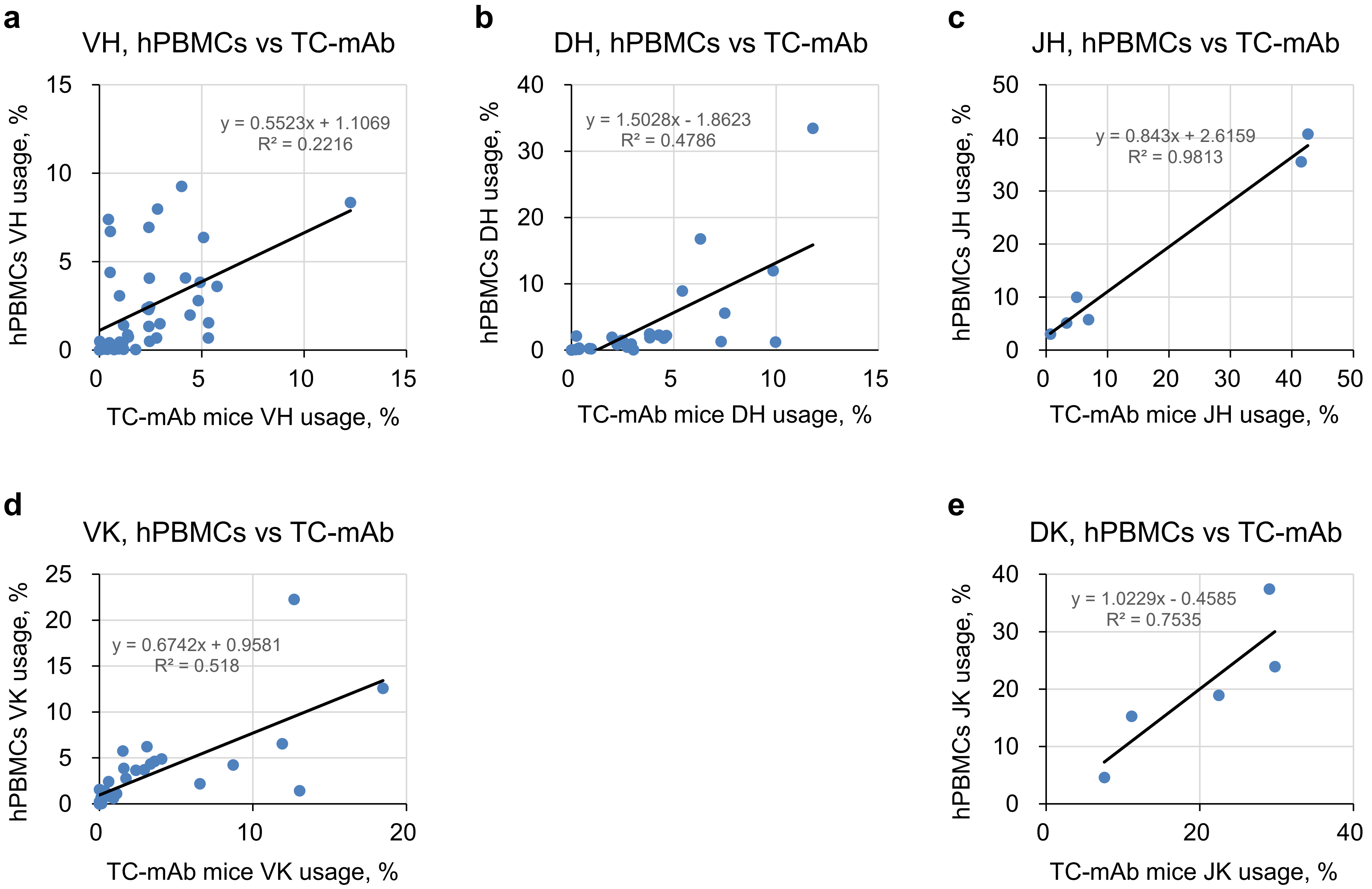

Supplementary Figure 10| Comparison with the V(D)J usage between hPBMCs and TC-mAb mice.
The frequency of VH **(a)** DH **(b)**, JH **(c)**, VK **(d)**, and JK **(e)** element is plotted and correlated between unimmunized TC-mAb mice (X-axis) and hPBMCs (Y-axis). R2 values are exhibited as correlation coefficient.

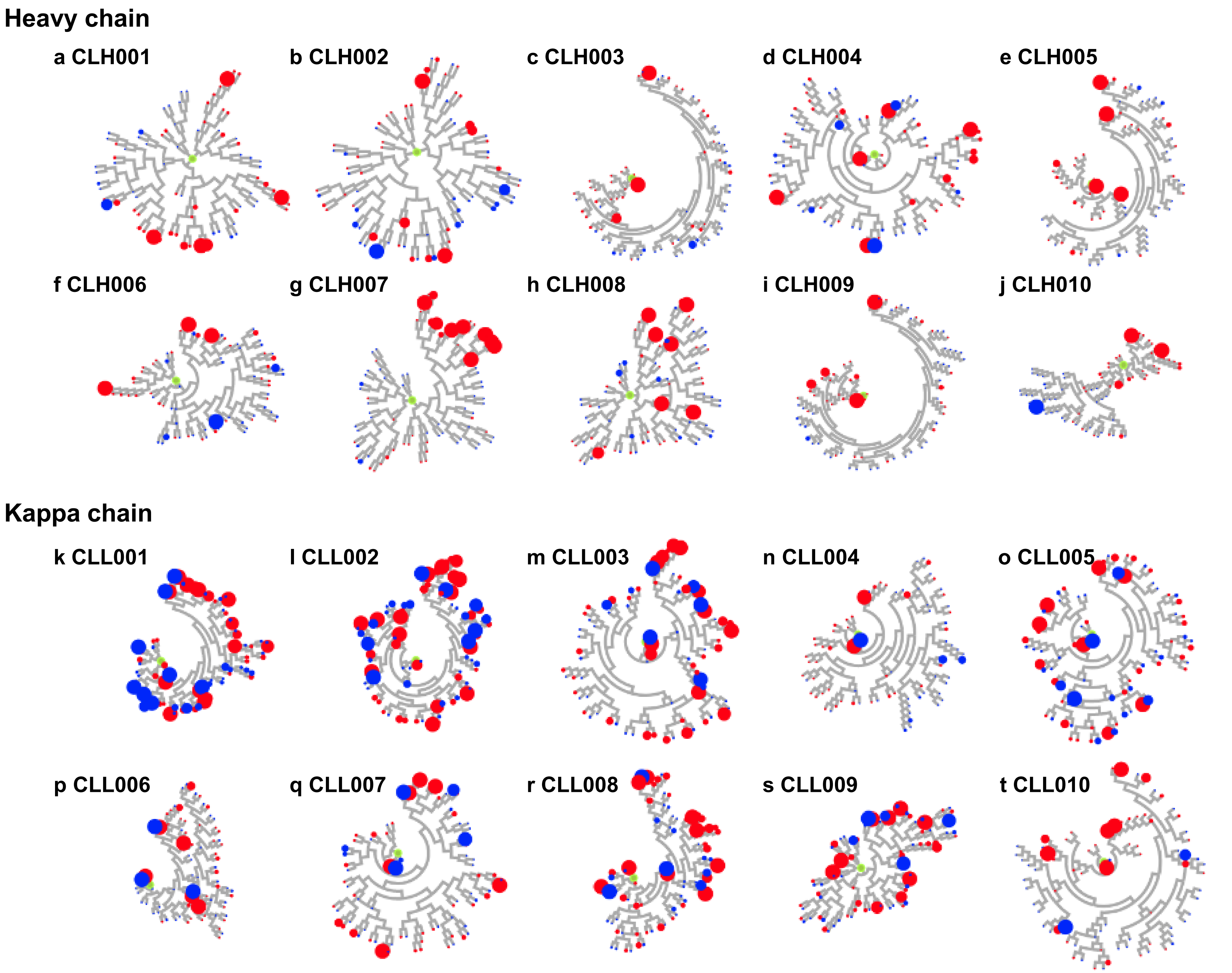

Supplementary Figure 11| Circular dendrogram and expanded clonotypes.
The phylogenetic trees of the CDRH3 and CDRL3 and expanded clonotypes were represented. The circular dendrograms of Top 20 frequently used clone lineages were over-rayed with the number of copies having the same CDR3 sequences in VH and VK, respectively. The results of CLH001-020 of heavy chain **(a-j)** and CLL001-020 of kappa chain **(k-t)** were represented. The leaves of circular dendrograms with a maximum of 50 reads, thereby the larger number drew the larger circle in the leaves. The color of the circle was indicated with (red) or without (blue) immunization in TC-mAb mice.

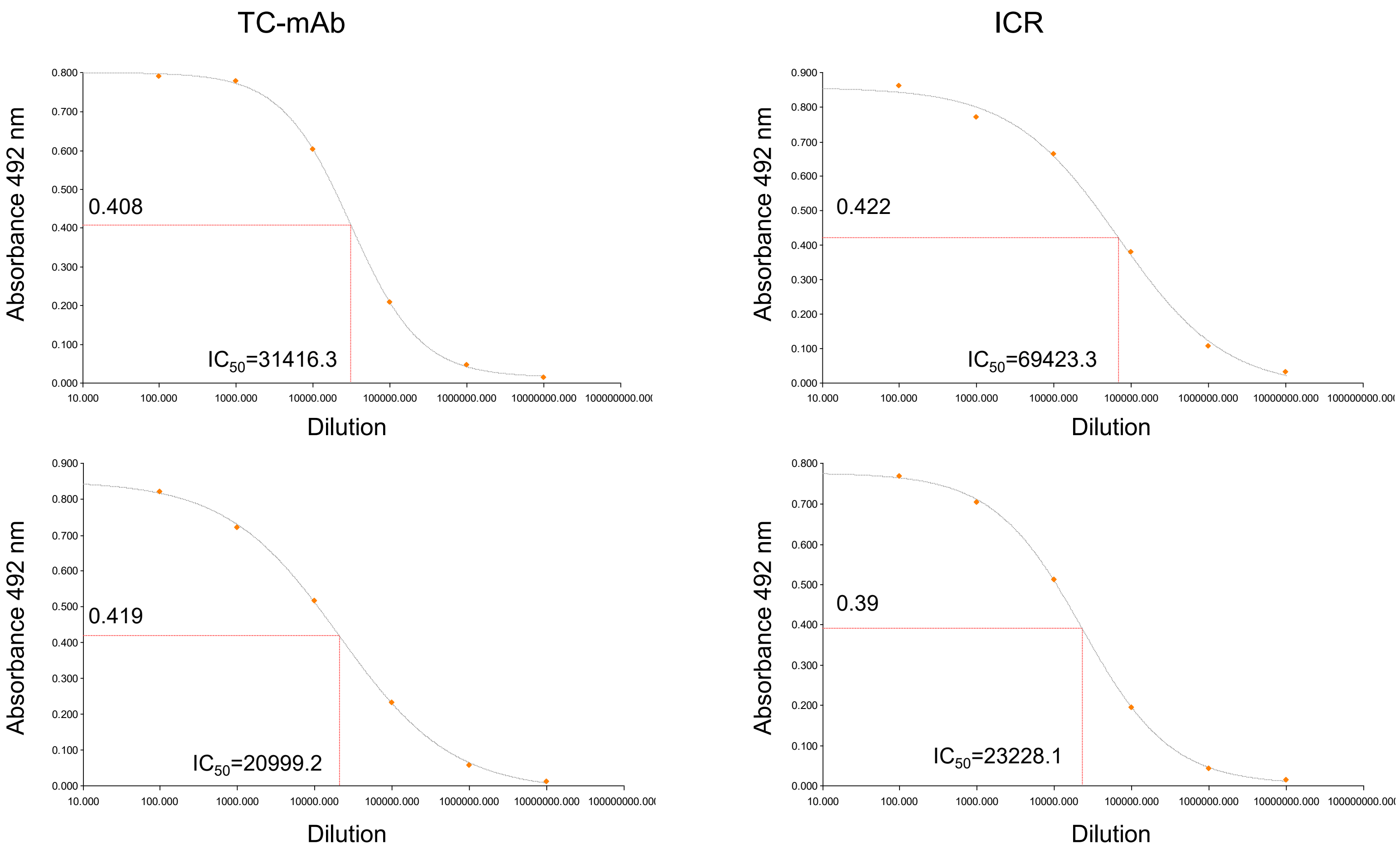

Curve Name	Y (Absorbance 492 nm)	X (Dilution of antisera)
**TC**	0.408	31416.3
	0.419	20999.2
**ICR**	0.422	69423.3
	0.390	23228.1

Supplementary Figure 12| Comparison of anti-sera affinities.
The effective concentration 50 (EC_{50}) of anti-sera obtained from TC-mAb (the two graphs on the left side) and ICR mice (the two graphs on the right side) are indicated. The titres of serially diluted OVA-specific anti-sera showed that the EC_{50} of respective anti-sera was calculated at a dilution of 2–7 in 10^4 by a 4-parameter logistic curve fitting model. The table is a summary of each absorbance and Ab concentration at EC_{50}.

a Trx-Amigo2-Ig

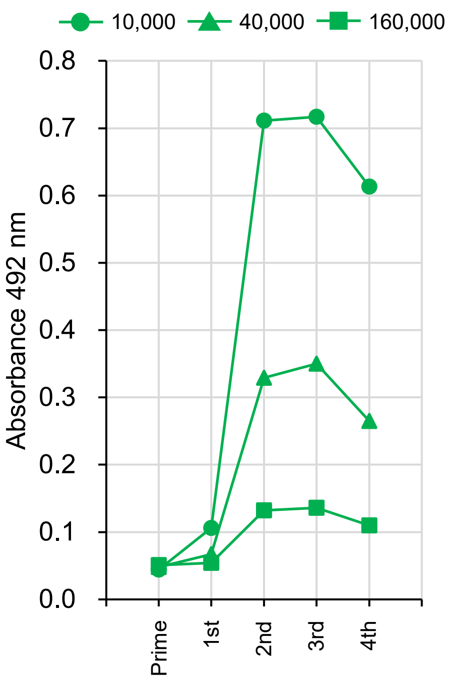

b GST-Amigo2-Ig

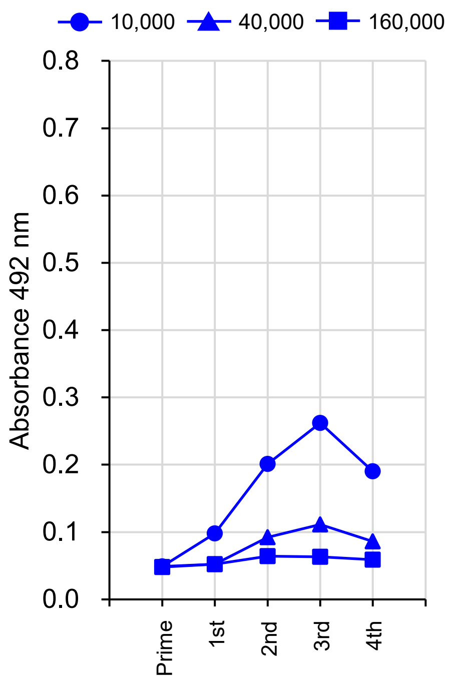

Table | Monoclonal antibody production against AMIGO2.

Mouse	TC-mAb
**Immunization**	Primary and 4 boosters
**Lymphocytes (cells/mouse)**	7.8×10^{7}
**Lymphocytes/cell fusion (cells)**	7.8×10^{7}
**ELISA positive well after HAT selection (well)**	144
**ELISA positive well after second screening (clones)**	123
**Determination of subclass (clones)**	55
**IgG1**	37
**IgG2**	1
**IgG3**	13
**IgG4**	0
**IgM**	3
**Bound to native antigen (clones)**	55
**Cloned by limiting dilution (clones) (Success rate of cloning)**	55 (100%)

Supplementary Figure 13| Summary of immunization, production and screening of anti-AMIGO2 mAbs. The titres of anti-sera (10,000 (circle), 40,000 (triangle), and 160,000 (square)-fold dilution) were analysed using the fusion proteins, Trx-AMIGO2-Ig **(a)** and GST-AMIGO2-Ig **(b)**. The Trx-AMIGO2-Ig titre indicated induction of antigen-specific Abs, but that of GST-AMIGO2-Ig indicated administration of AMIGO2-Ig-specific Abs. In the table, the numbers of hybridoma cells (clones) are indicated when TC-mAb mouse was immunized with the Trx-AMIGO2-Ig fusion protein as antigen. A portion of the harvested lymphocytes was used for cell fusion, as indicated in the 'lymphocytes/cell fusion' row. After selection with HAT medium, the antigen-positive wells, which contained a few of hybridoma cell colonies, were screened and hybridoma cells were picked from those wells. After a second screening to check the antigen-specific binding of Abs, positive hybridoma cells were analysed for subclass determination. After analysing the binding activity of Abs to native AMIGO2 on the cell surface of CHO cells transfected with pcDNA3.1 vector containing the AMIGO2 whole region, the positive mAb-producing hybridoma cells were established by the limiting dilution procedure. The number of established clones is indicated in the last row.

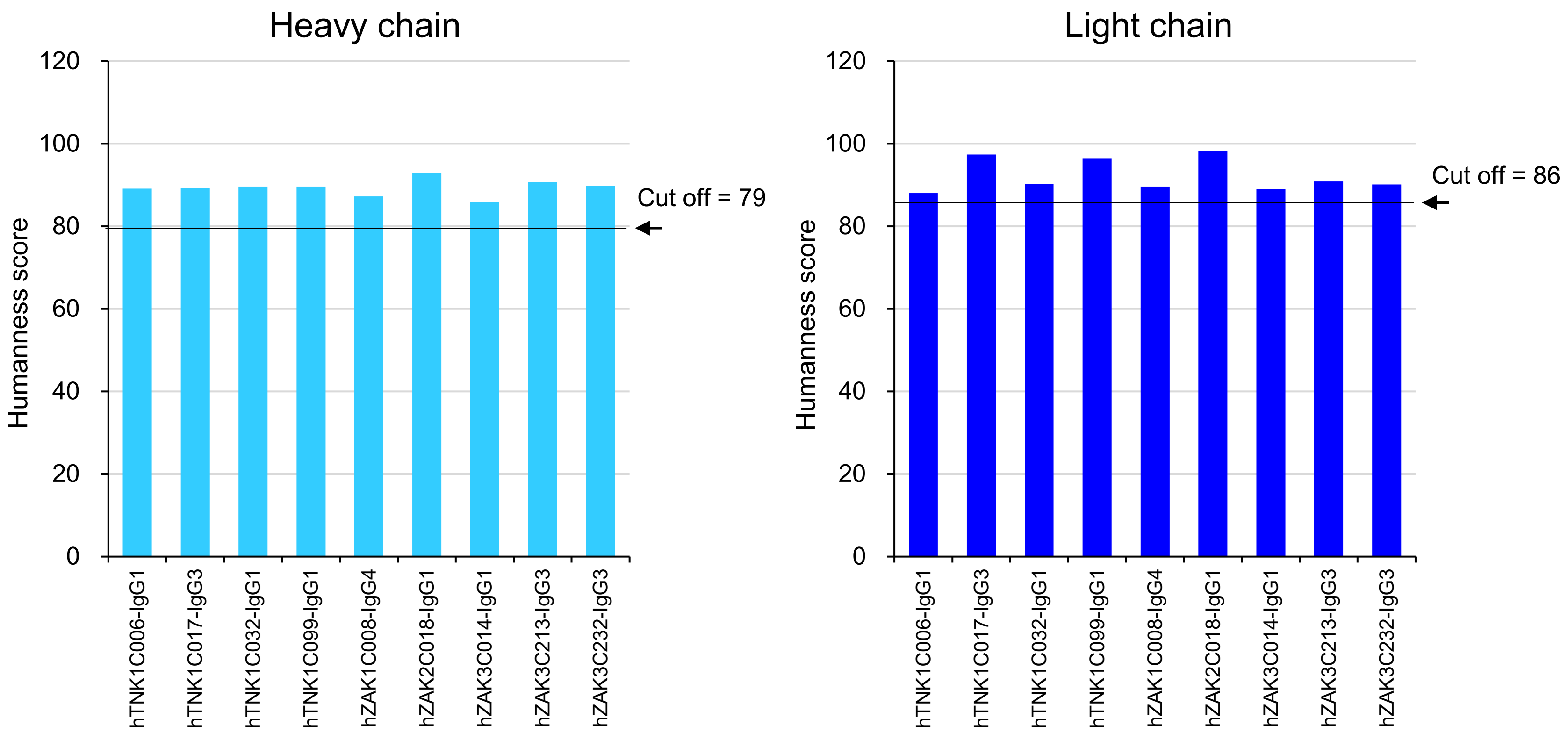

		Heavy chain		Light chain	
**Clone name**	**Subclass**	VH gene	CDR3H length	VL gene	CDR3L length
hTNK1C006-IgG1	IgG1	IGHV3-33*01|D3-10*01|J4*02	16	IGKV2-29*02|J3*01	8
hTNK1C017-IgG3	IgG3	IGHV4-59*01|D3-10*01|J6*02	19	IGKV4-1*01|J2*01	9
hTNK1C032-IgG1	IgG1	IGHV3-33*01|D3-10*01|J4*02	16	IGKV2-29*02|J3*01	8
hTNK1C099-IgG1	IgG1	IGHV4-59*01|D3-10*01|J6*02	19	IGKV4-1*01|J2*01	9
hZAK1C008-IgG4	IgG4	IGHV3-13*01|D7-27*01|J6*02	14	IGHKV3-20*01|J3*01	9
hZAK2C018-IgG1	IgG1	IGHV3-13*01|D7-27*01|J6*02	14	IGKV3-11*01|KJ2*01	9
hZAK3C014-IgG1	IgG1	IGHV3-73*02|D3-10*01|J6*02	19	IGKV2D-29*02|J1*01	9
hZAK3C213-IgG3	IgG3	IGHV3-13*01|D5-24*01|J6*02	14	IGKV1-27*01|J3*01	9
hZAK3C232-IgG3	IgG3	IGHV3-13*01|D5-24*01|J6*02	14	IGKV1-27*01|J3*01	9

Supplementary Figure 14| Humanness of mAbs.
Humanness scores of mAb isolated from TC-mAb mice were indicated. The nucleotide sequences of VH and VK region of mAbs isolated from TC-mAb mice were determined for anti-EpCAM mAbs (hZAK1C008, hZAK2C018, hZAK3C014, hZAK3C213, and hZAK3C232) and anti-AMIGO2 mAbs (hTNK1C006, hTNK1C017, hTNK1C032, and hTNK1C099). The T20 scores were determined using the T20 score analyser. To make a judgment as a human sequence, cut off values (Heavy chain was 79 and κ chain was 86) was used according to the threshold set by authors. The thresholds were indicated as arrows. The table was the summary of antibodies sequence including their subclass, matched germline genes of VH and VK, and CDR length.

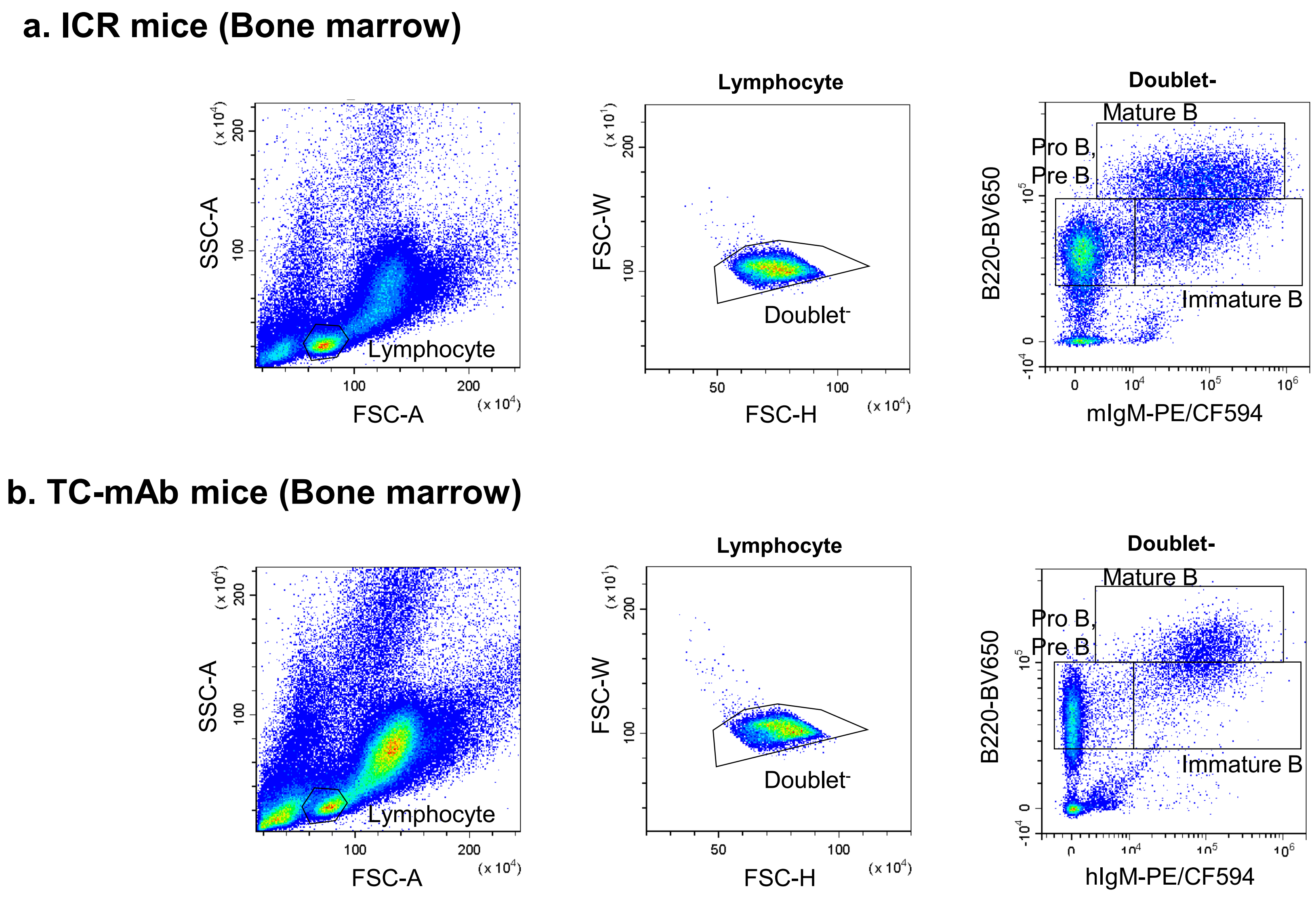

Supplementary Figure 15| Flow cytometry identification of PreB/ProB, immature B and mature B cells.
Flow cytometry gating strategies for in spleen cells of Pre B/Pro B cells ($B220^{lo}IgM^{-}$), Immature B cells ($B220^{lo}IgM^{+}$) and mature B cells ($B220^{hi}IgM^{+}$) in bone marrow cells of ICR mice (**a**) and TC-mAb mice (**b**). The same panels of B220-IgM are presented on Figure 7a.

a. ICR mice (Bone marrow)

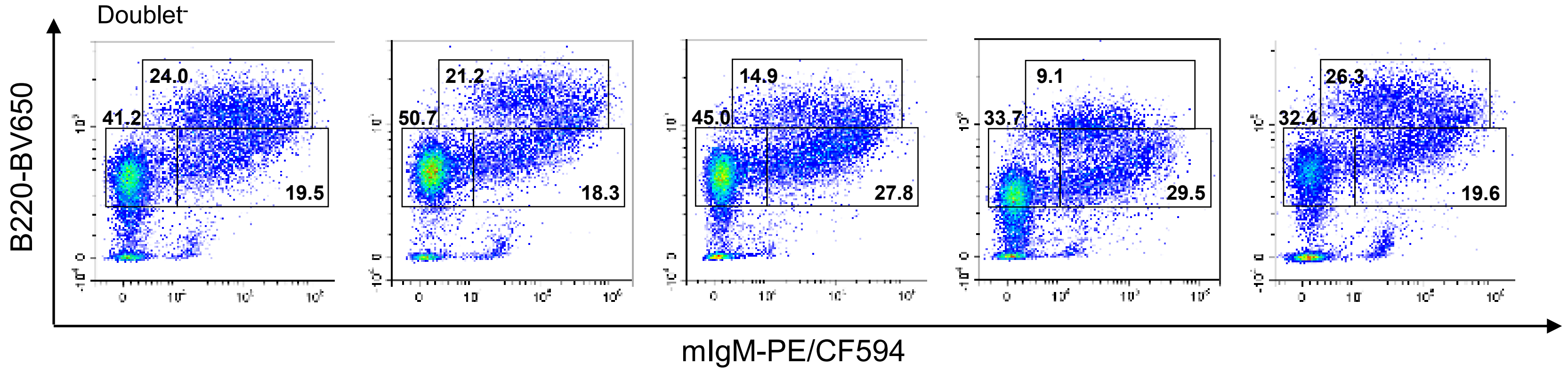

b. TC-mAb mice (Bone marrow)

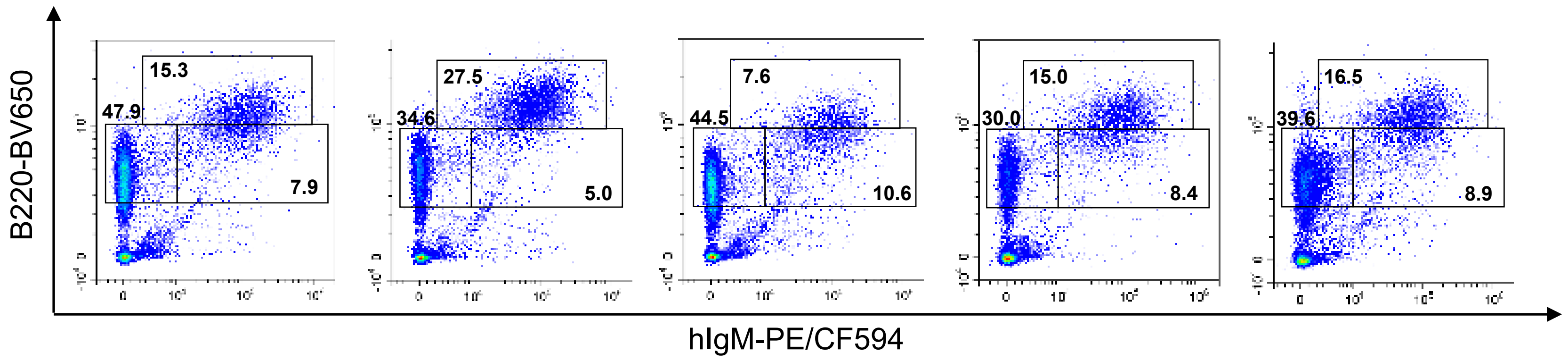

Supplementary Figure 16| Representation of flow cytometry in ProB/PreB, immature B and mature B cells in the bone marrow cells.

The compartments of lymphocyte, of ProB/PreB cells (B220^{lo}IgM^{-}), Immature B cells (B220^{lo}IgM^{+}) and mature B cells (B220^{hi}IgM^{+}) in bone marrow cells of 20-22 weeks-age ICR mice (n=5) **(a)** and TC-mAb mice (n=5) **(b)**. Numbers in the flow cytometry results indicate the percentage of each B cell subset(s).

a. ICR mice (Spleen)

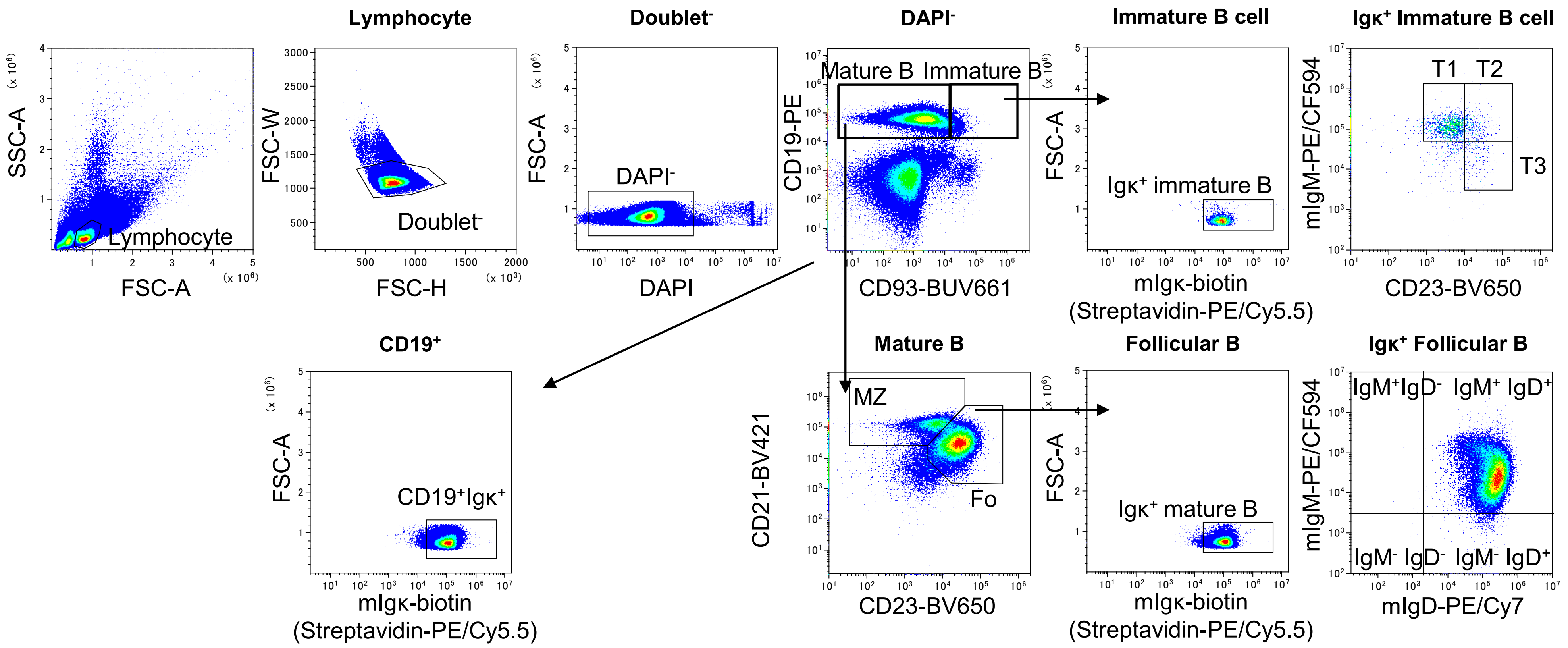

b. TC-mAb mice (Spleen)

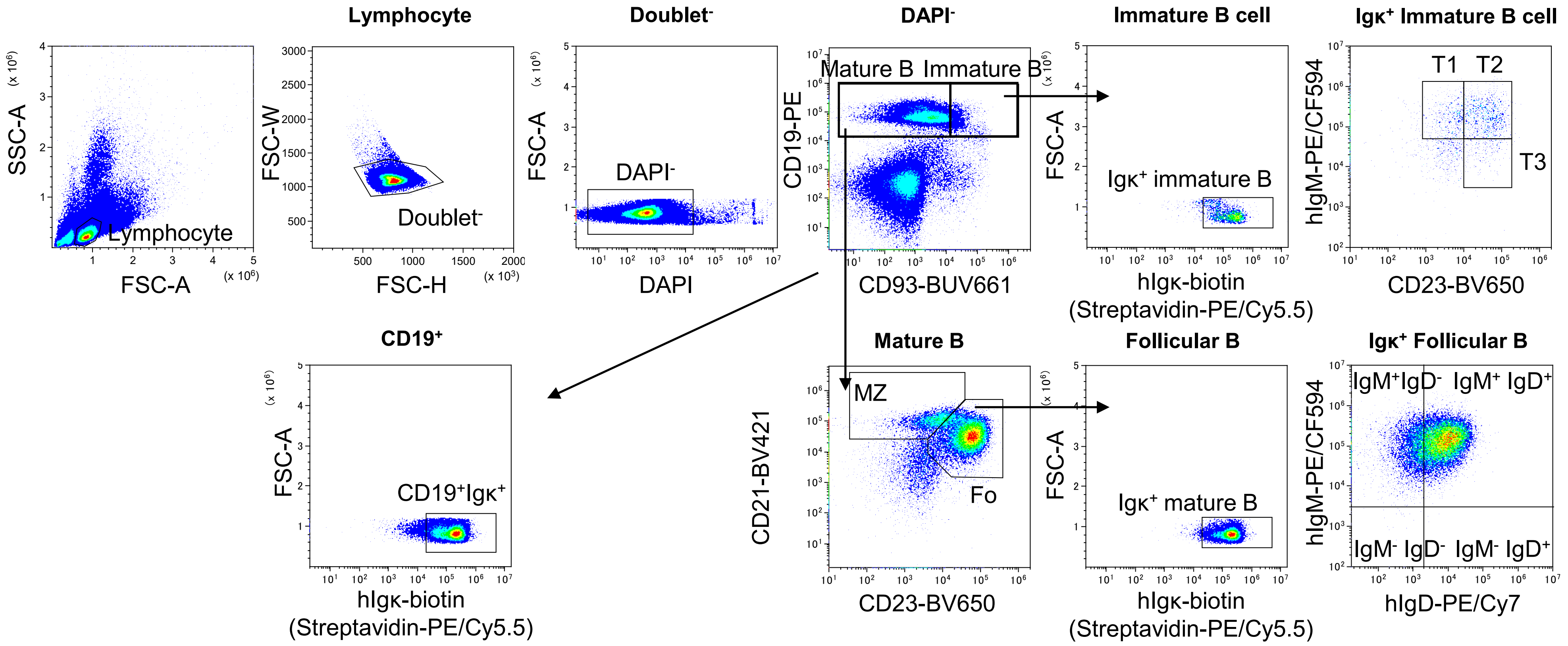

Supplementary Figure 17| Flow cytometry identification of mature B, immature B and transitional (T1, 2, and 3) B cells. Flow cytometry gating strategies for mature B, immature B, and transitional B cells in spleen cells of ICR mice **(a)** and TC-mAb mice **(b)**. The same of CD19-CD93, CD21-CD23, IgM-CD23 (Igκ+ immature B cells) panels are presented on Figure 7b.

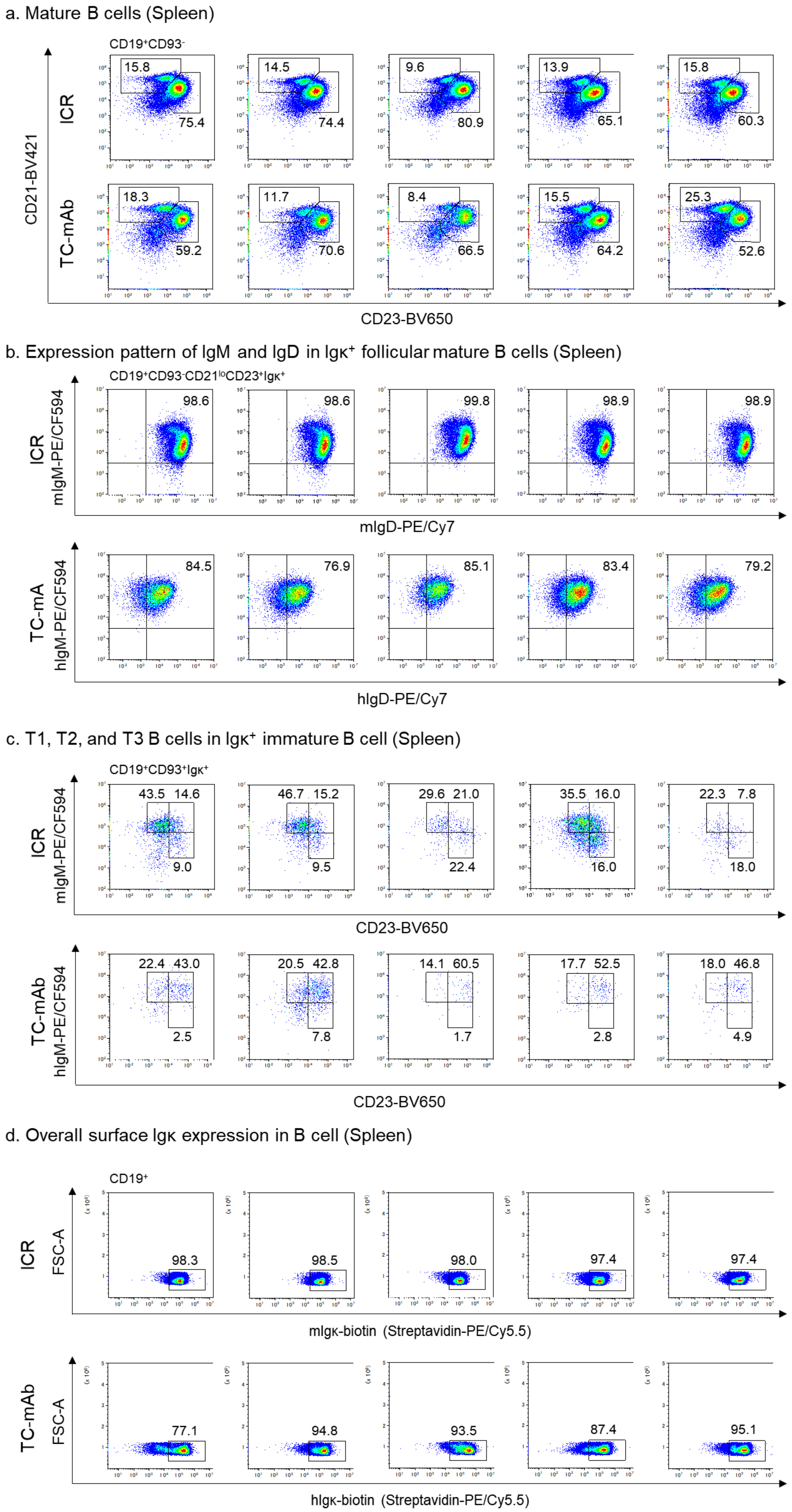

Supplementary Figure 18| Representation of flow cytometry in mature B, immature B, and transitional (T1, 2, and 3) B cells in the spleen cells.

The compartments of lymphocyte, mature (CD19^{+}CD93^{-}), and immature (Igκ^{+}CD19^{+}CD93^{+}) B cells in the spleen of 8-9 weeks-age ICR mice (n=5) and TC-mAb mice (n=5). Numbers in the flow cytometry results indicate the percentage of each B cell subset(s). Follicular B (CD19^{+}CD93^{-}CD21^{lo}CD23^{hi})and marginal zone B (CD19^{+}CD93^{-}CD21^{hi}CD23^{lo}) cells in mature B cells **(a)**, expression pattern of IgM and IgD in Igκ^{+} follicular B cells **(b)**, and transitional (T1 (IgM^{hi}CD23^{-}), T2 (IgM^{hi}CD23^{+}), T3 (IgM^{lo}CD23^{+})) B cells in Igκ^{+} immature B cells **(c)**. **(d)** Overall expression level of surface Igκ in spleen B cells (CD19^{+}). The data collected from ICR mice (five panels of upper row) and TC-mAb mice (five panels of lower row) are indicated, respectively. The statistical graphs of the percentages of B cell subsets are presented on Figure 7c.

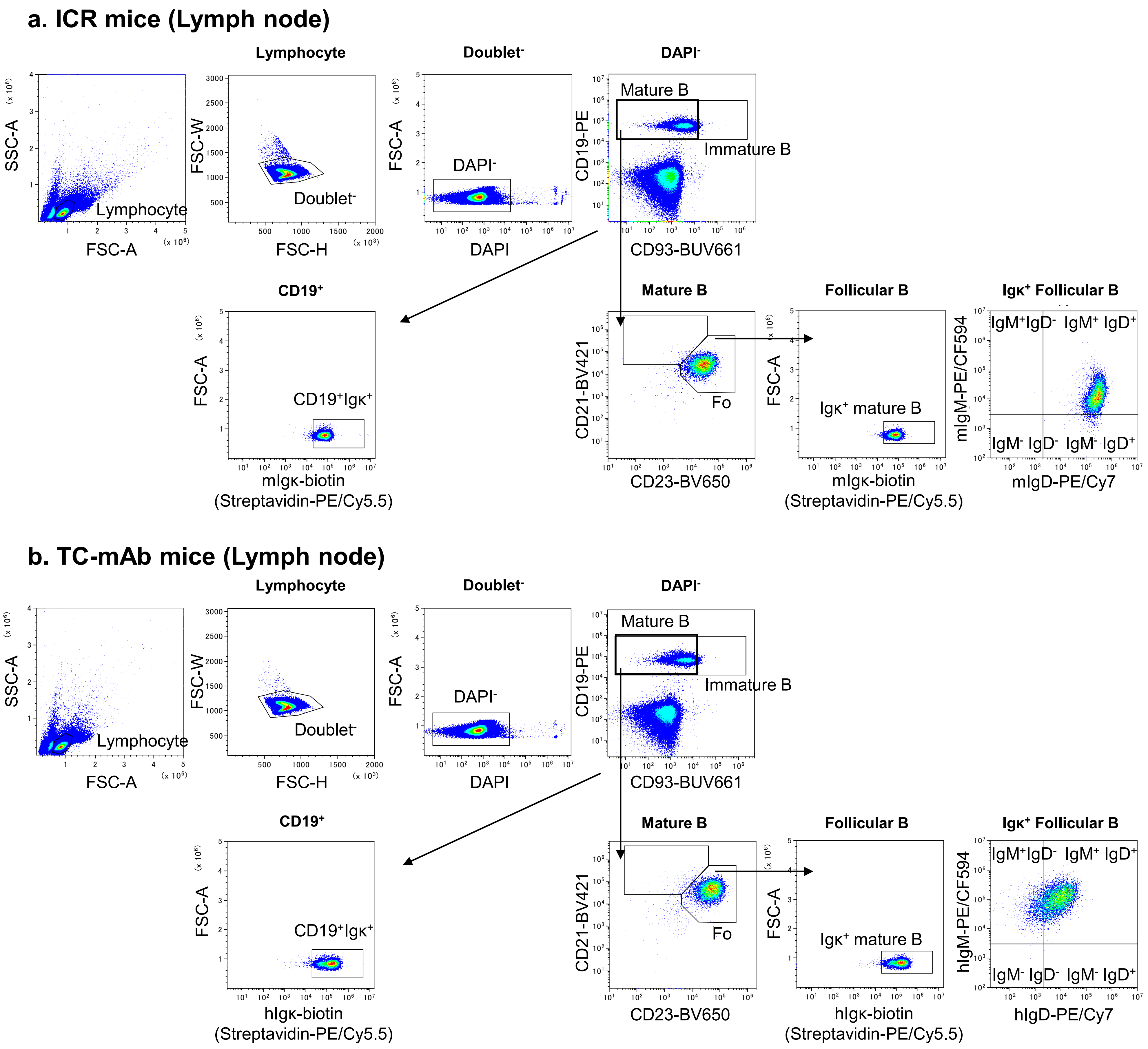

Supplementary Figure 19| Flow cytometry identification of mature B, immature B and transitional (T1, 2, and 3) B cells. Flow cytometry gating strategies for mature B, immature B, and transitional B cells in lymph node cells of ICR mice **(a)** and TC-mAb mice **(b)**.

a. Mature B cells (Lymph node)

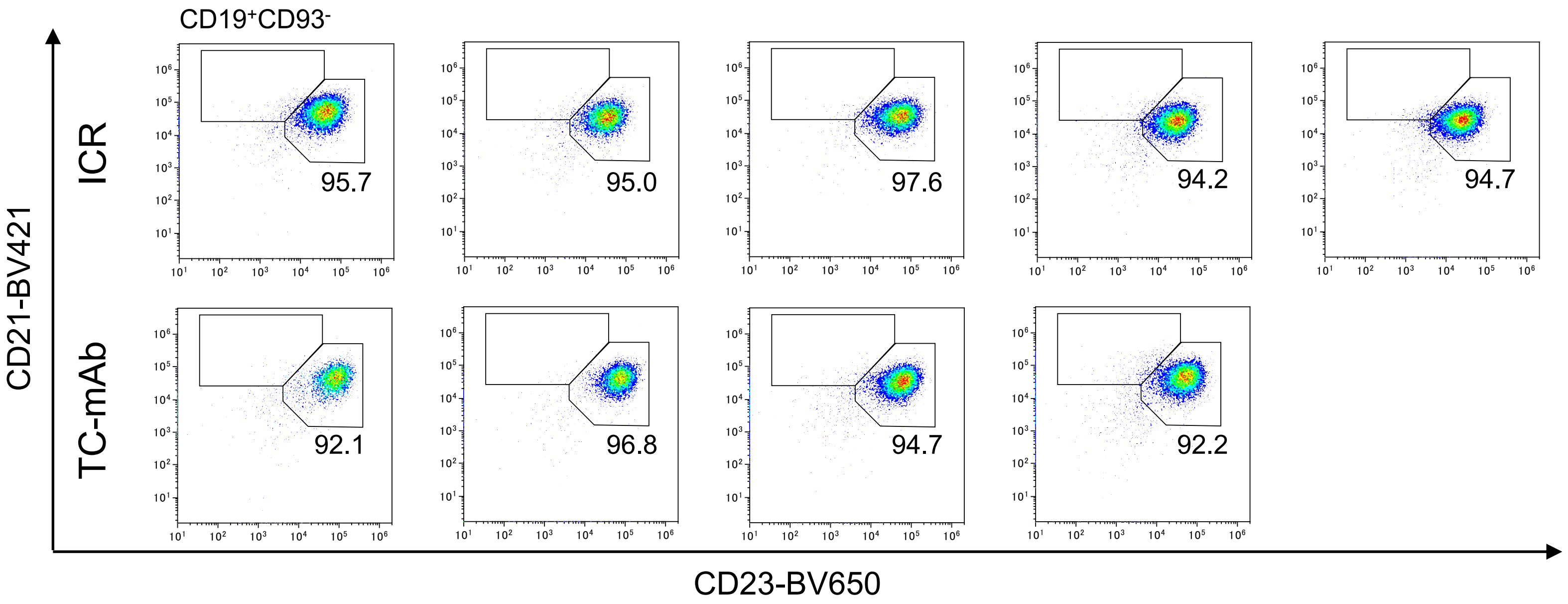

b. Expression pattern of IgM and IgD in $Ig\kappa^{+}$ follicular mature B cells (Lymph node)

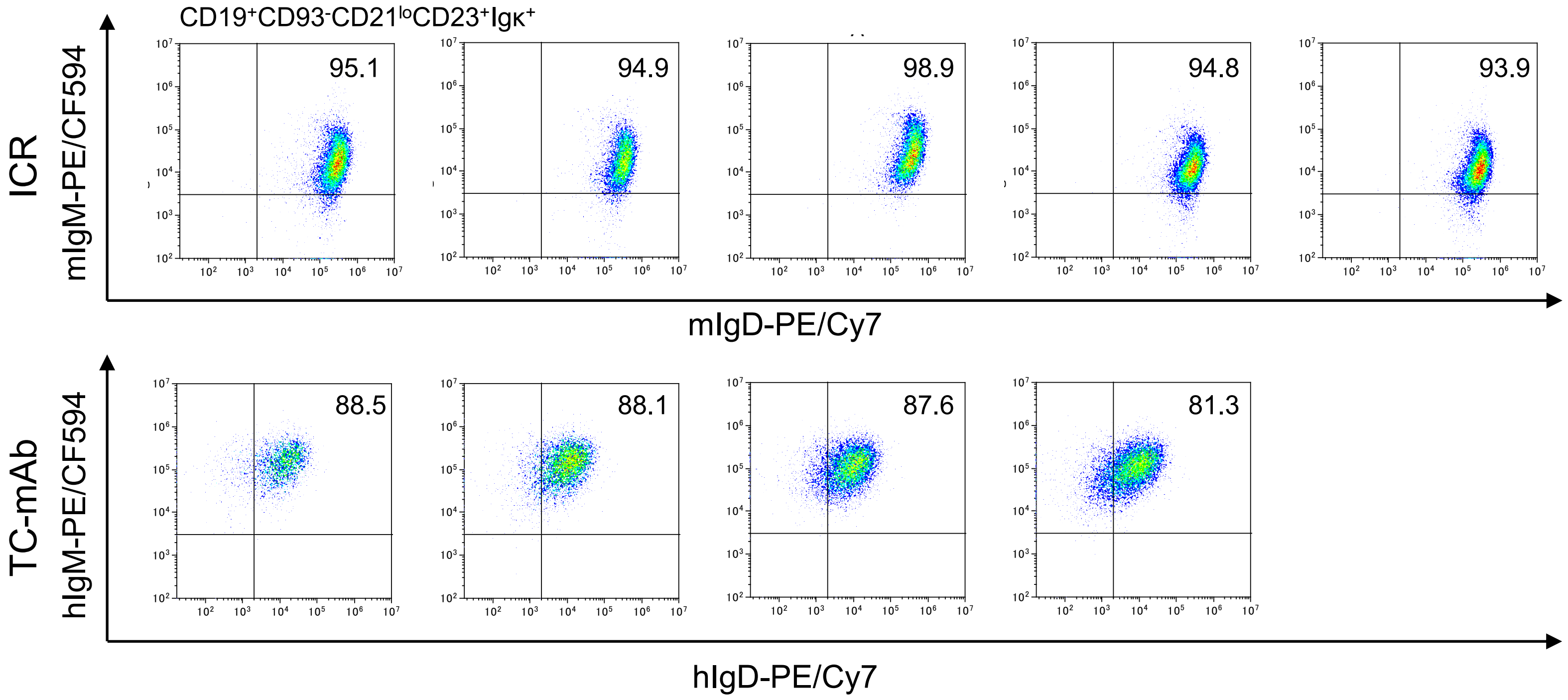

c. Overall surface Igκ expression level in B cells (Lymph node)

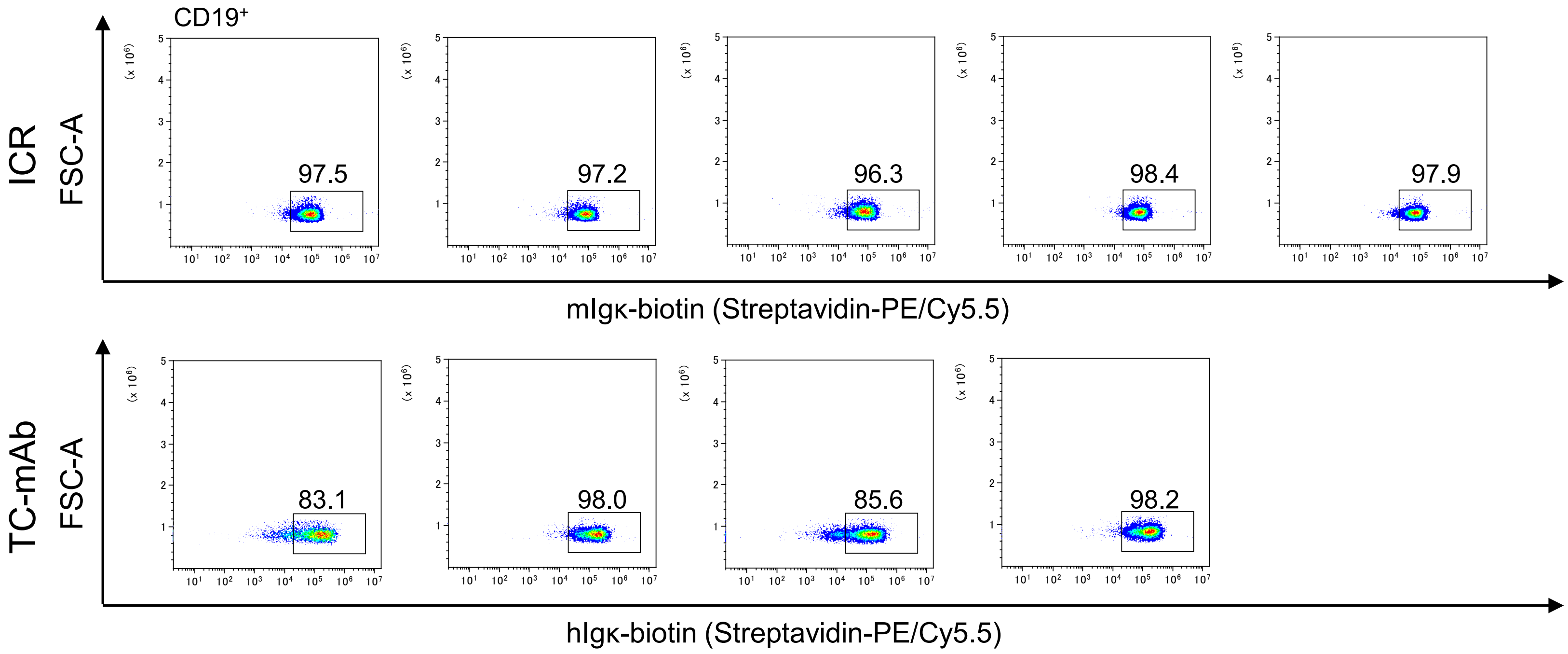

Supplementary Figure 20| Representation of flow cytometry in mature B, and transitional (T1, 2, and 3) B cells in the lymph node cells.

The compartments of lymphocyte, mature ($CD19^{+}CD93^{-}$) cells in the spleen of 8-9 weeks-age ICR mice (n=5) and TC-mAb mice (n=4). Numbers in the flow cytometry results indicate the percentage of each B cell subset(s). Follicular B ($CD19^{+}CD93^{-}CD21^{lo}CD23^{hi}$) of mature B cells in the Lymph node cells **(a)**,expression pattern of IgM and IgD in $Ig\kappa^{+}$ follicular B **(b)**. **(c)** Overall expression level of surface Igκ in lymph node B cells ($CD19^{+}$). The data collected from ICR mice (five panels of upper row) and TC-mAb mice (four panels of lower row) are indicated, respectively. The statistical graph of the percentages of follicular B cell subsets is presented on Figure 7c.

a. ICR mice (8 week-age, spleen)

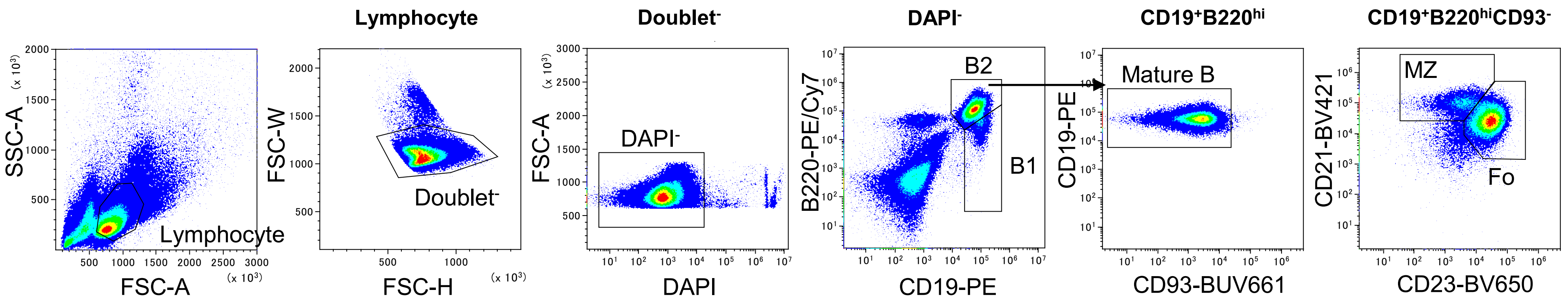

b. TC-mAb mice (8 week-age, spleen)

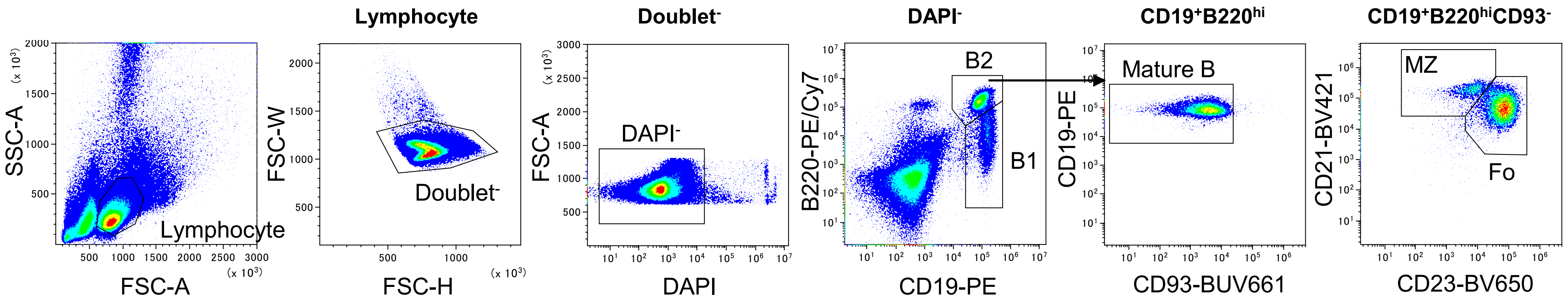

Supplementary Figure 21| Flow cytometry identification of follicular and marginal zone B cells in spleen cells.
Flow cytometry gating strategies for follicular and marginal zone B cells in the spleen. The distribution were analysed using 8 weak-age of ICR **(a)** and TC-mAb mice **(b)**.

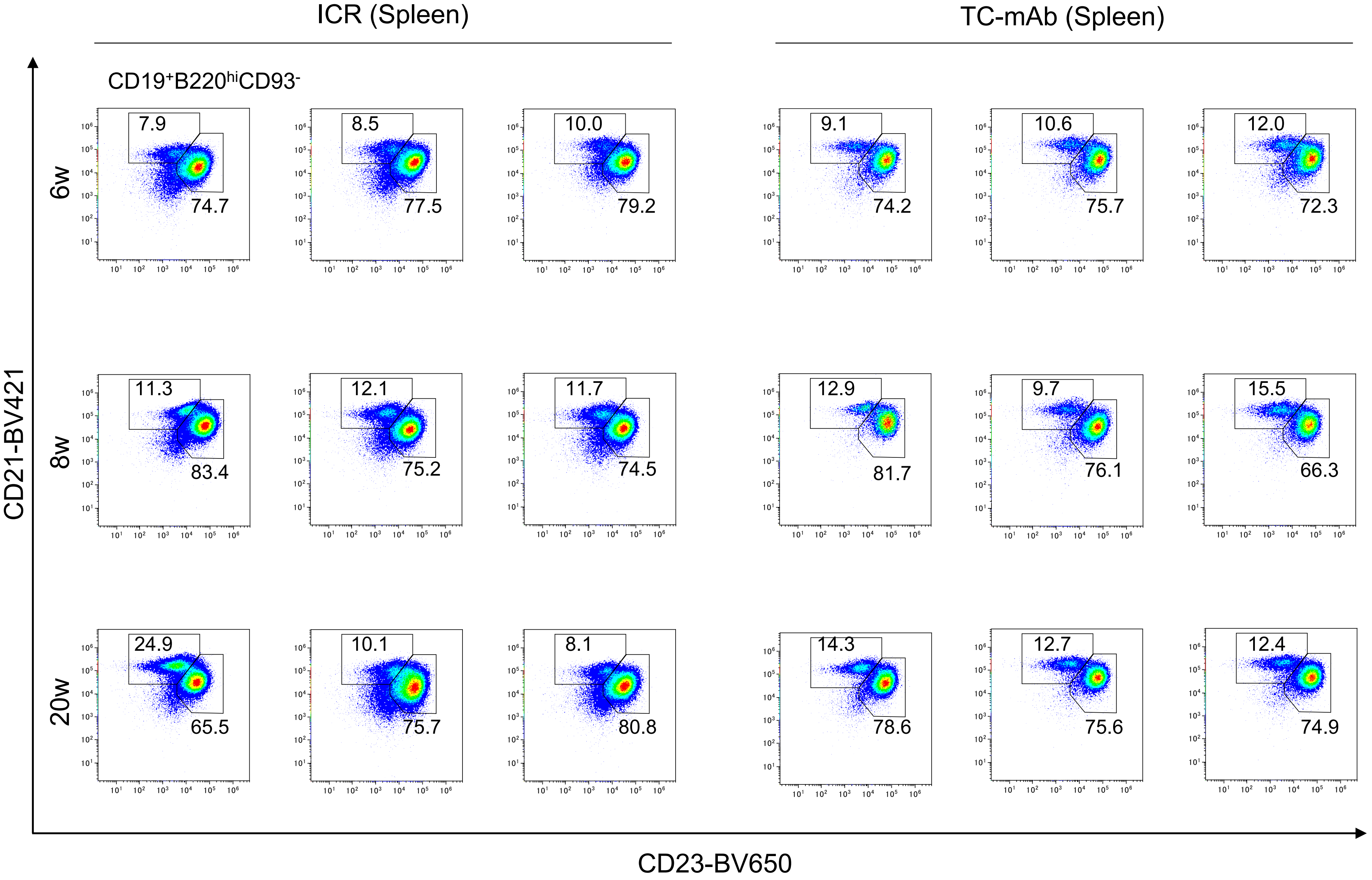

Supplementary Figure 22| Representation of flow cytometry in follicular and marginal zone B cells in the spleen cells. The distribution of follicular (CD21^{lo}CD23^{hi}) and marginal zone (CD21^{hi}CD23^{lo}) B cells in the spleen mature B (CD19^{+}B220^{hi}CD93^{-}) of TC-mAb mice (n=3) and ICR mice (n=3). The data collected from 8-9 weeks-age ICR mice (three panels of left side) and TC-mAb mice (three panels of right side) are indicated, respectively. The percentage of B cell subsets are represented in marginal zone and follicular B cells in the spleen cell. The weak-ages of analysed mice are indicated at left side of panels. The statistical graphs of the cell numbers of B cell subsets are presented on Figure 7d.

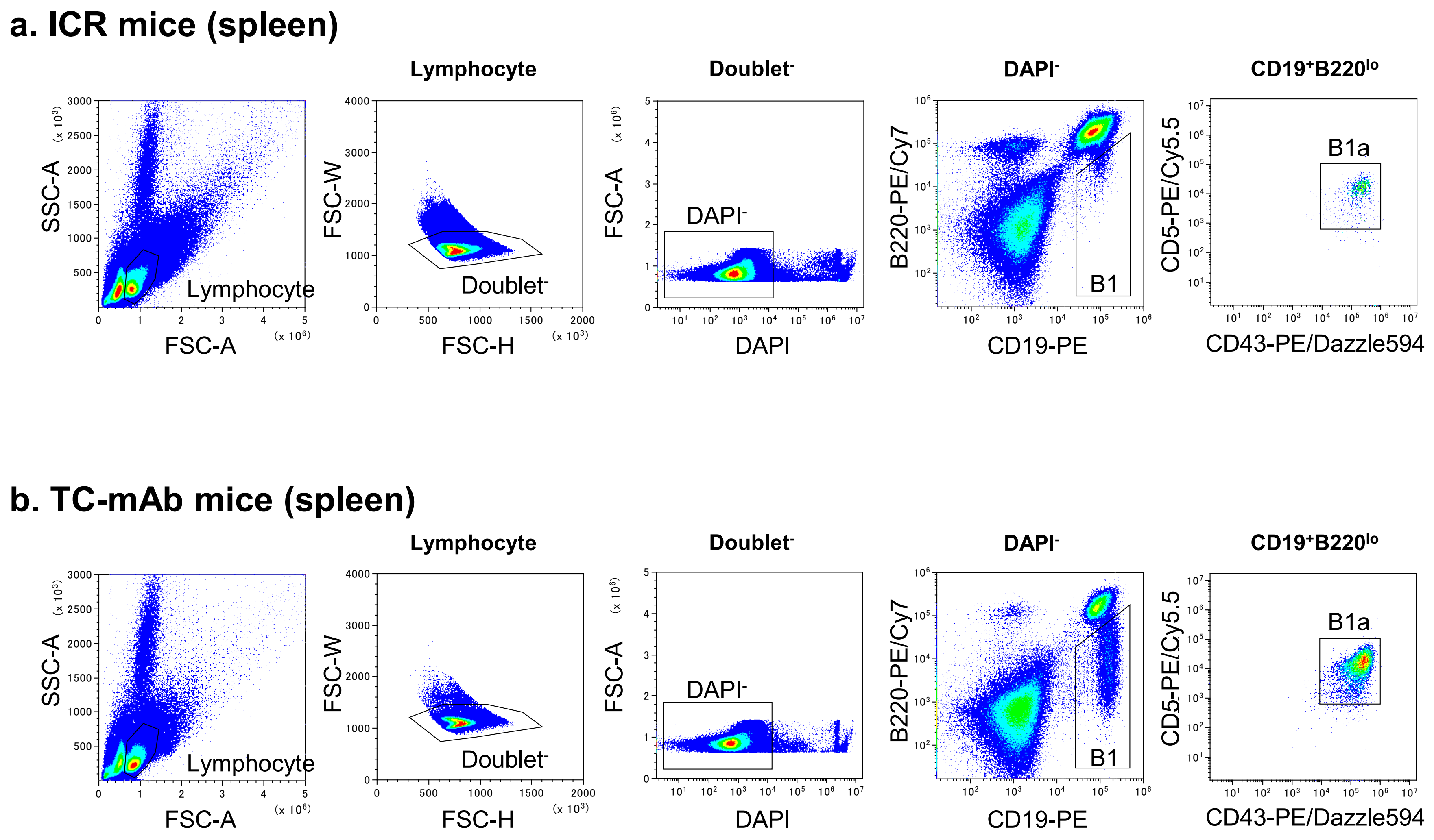

Supplementary Figure 23| Flow cytometry identification of B1a in spleen cells.
Flow cytometry gating strategies for B1a B cells in the spleen cells. The distribution were analysed using 8 weak-age of ICR **(a)** and TC-mAb mice **(b)**.

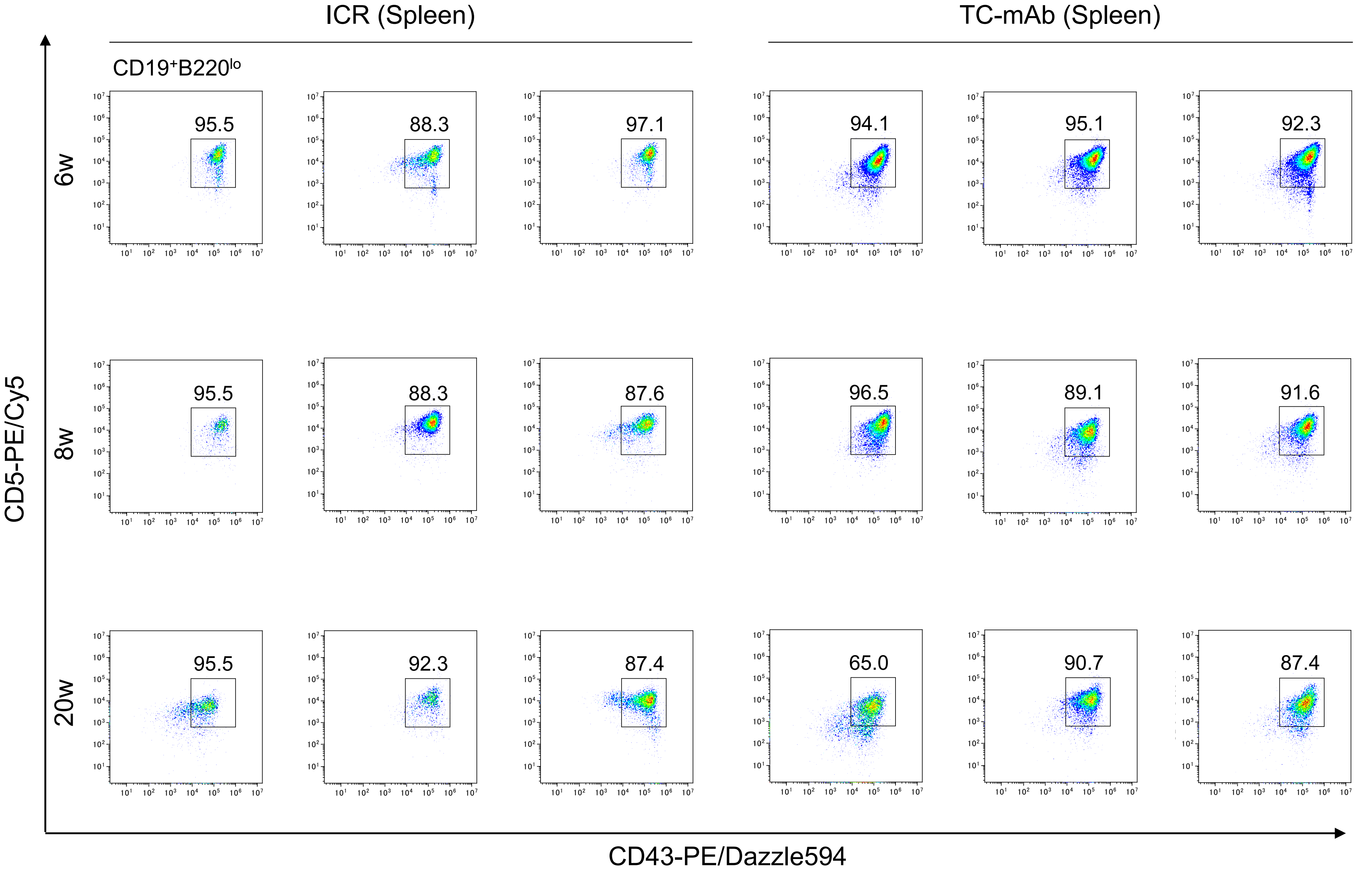

Supplementary Figure 24| Representation of flow cytometry in B1a in the spleen cells.
The distribution of B1a B cells ($CD5^{+}CD43^{+}$) in the spleen cells($CD19^{+}B220^{lo}$) of ICR (n=3) and TC-mAb mice (n=3). The data collected from 8-9 weeks-age ICR mice (three panels of left side) and TC-mAb mice (three panels of right side) are indicated, respectively. The percentage of B cell subsets are represented in B1a B cells in the spleen cell. The weak-ages of analysed mice are indicated at left side of panels. The statistical graph of the cell numbers of B1a B cell subsets is presented on Figure 7e.

a. ICR mice (8 week-age, PECs)

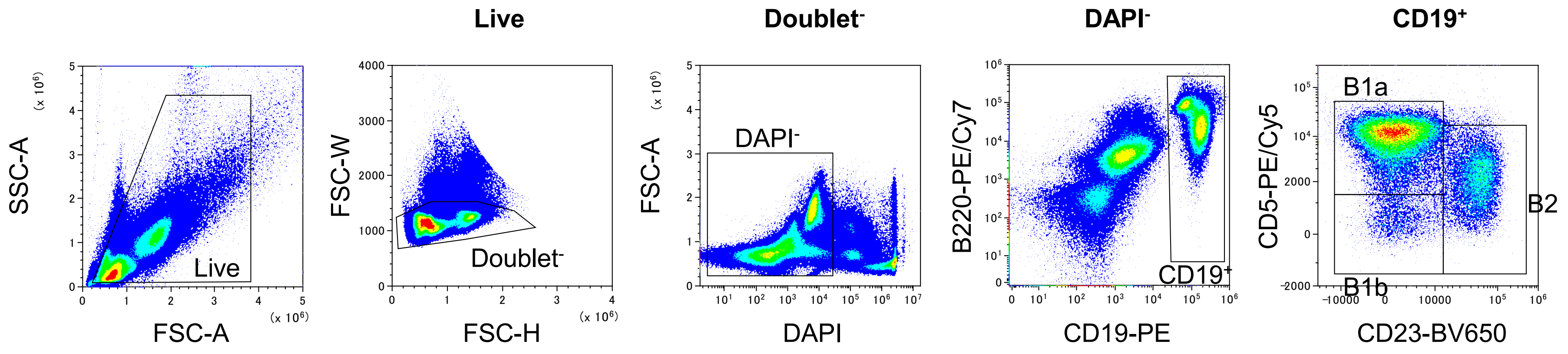

b. TC-mAb mice (8 week-age, PECs)

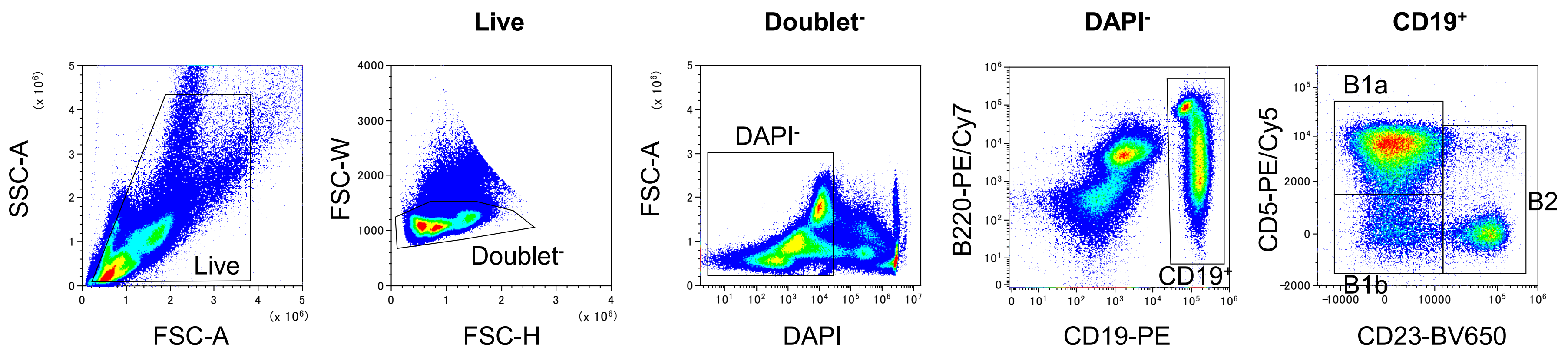

Supplementary Figure 25| Flow cytometry identification of B1a/b and B2 B cells in peritoneal exudate cells (PECs) cells. Flow cytometry gating strategies for B1a/b and B2 B cells in the PECs cells. The distribution were analysed using 8 weak-age of ICR **(a)** and TC-mAb mice **(b)**.

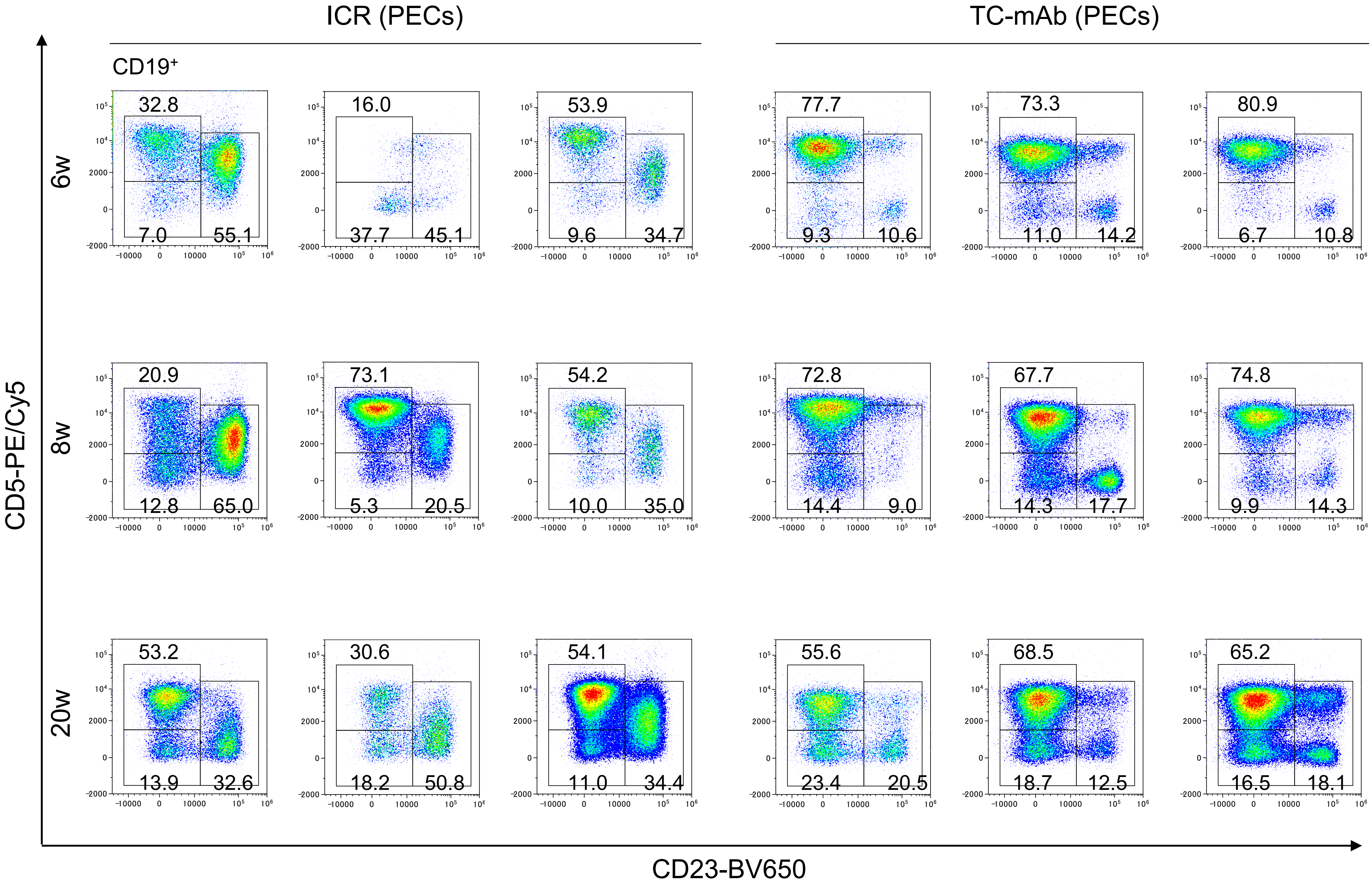

Supplementary Figure 26| Representation of flow cytometry in B1a/b and B2 B cells in the peritoneal exudate cells (PECs) cells.
The distribution of B1a ($CD5^-CD23^-$),B1b ($CD5^+CD23^-$) and B2 B ($CD5^-CD23^+$) cells in the PECs B cells ($CD19^+$) of ICR (n=3) and TC-mAb mice (n=3). The data collected from ICR mice (three panels of left side) and TC-mAb mice (three panels of right side) are indicated, respectively. The percentage of B cell subsets are represented in B1a, B1b, and B2 B cells in the PECs. The statistical graphs of the cell numbers of B cell subsets are presented on Figure 7e.

a. ICR mice (day 14 after immunization, spleen)

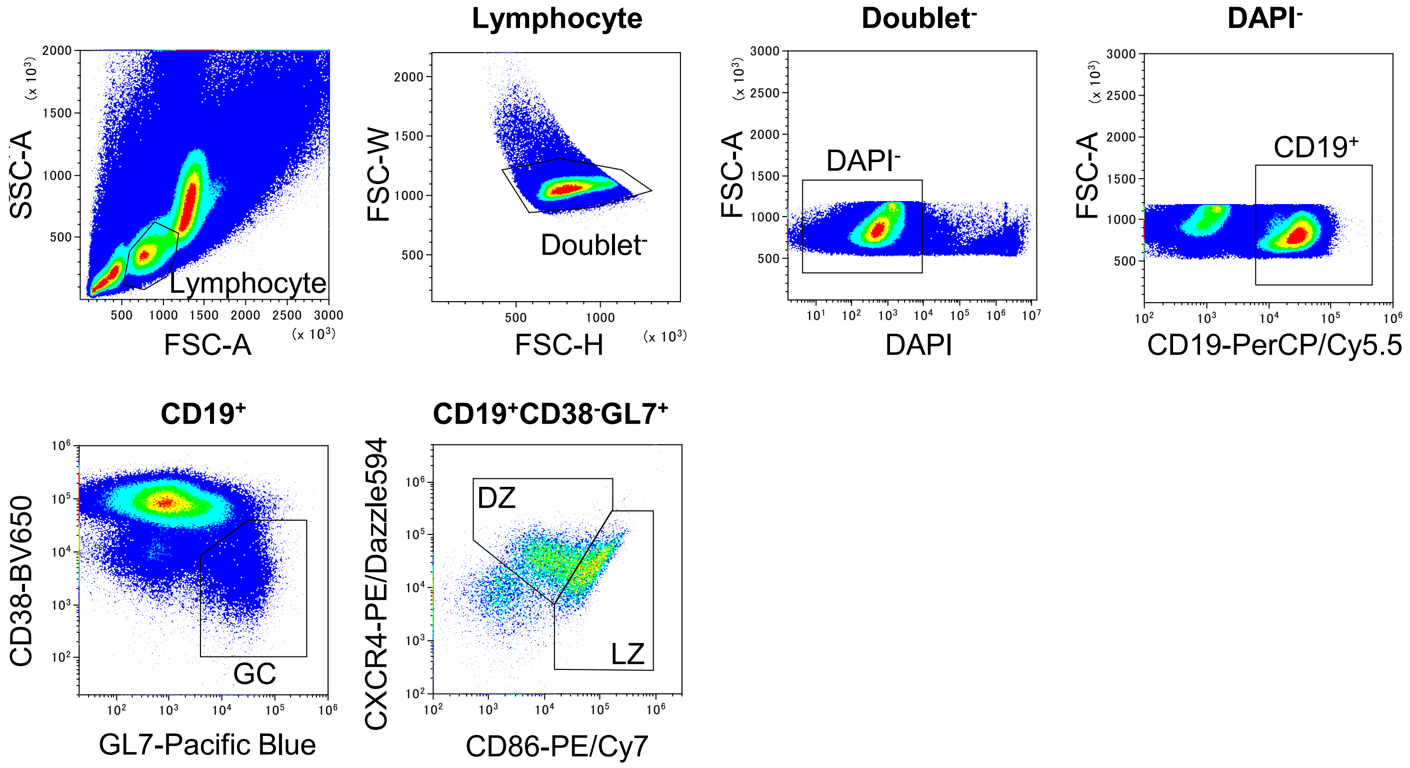

b. TC-mAb mice (day 21 after immunization, spleen)

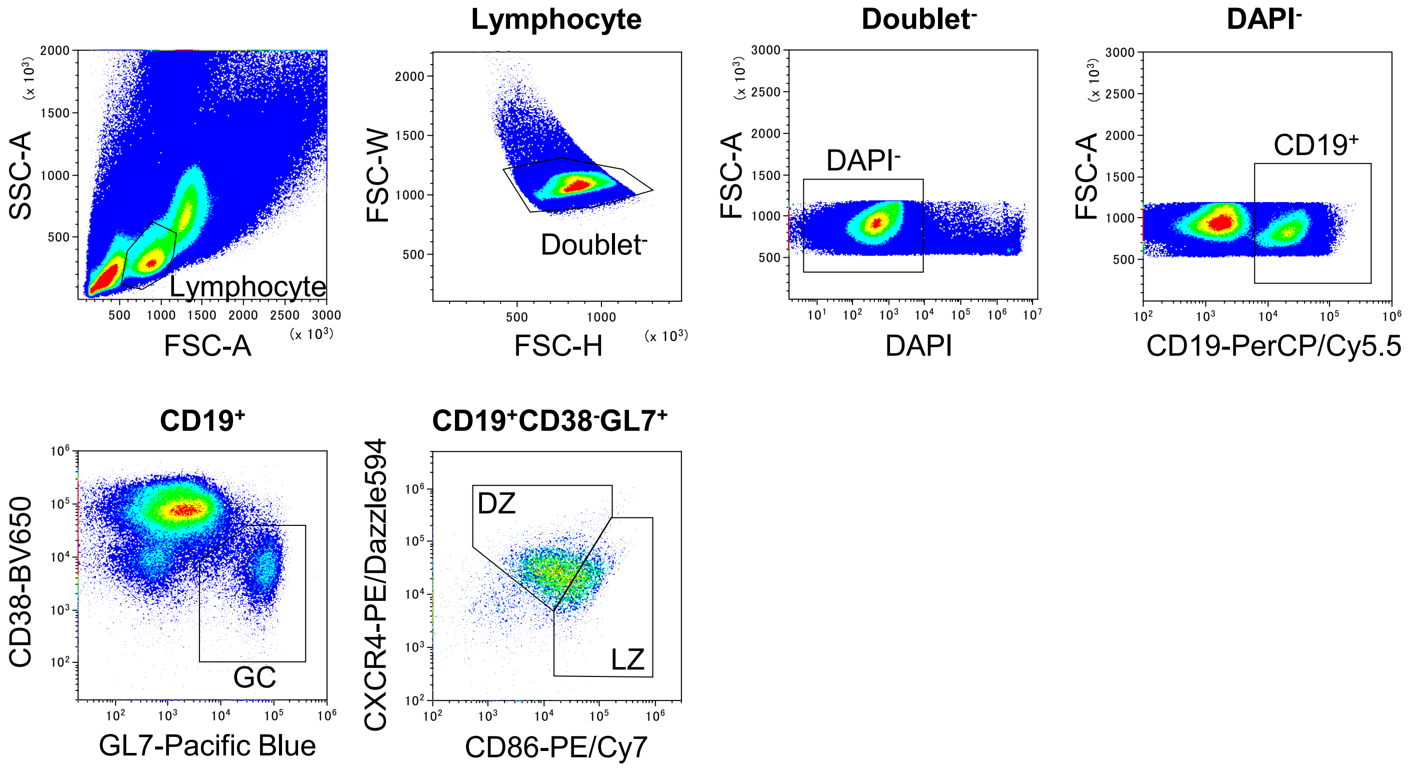

Supplementary Figure 27| Flow cytometry identification of germinal centre (GC) B cells and dark zone (DZ) and light zone (LZ) in spleen cells.

Flow cytometry gating strategies for GC B cells and DZ and LZ in spleen cells of ICR **(a)** and TC-mAb mice **(b)** after primary immunization with OVA. The same of CD38-GL7, CXCR4-CD86 panels are presented on Figure 8a.

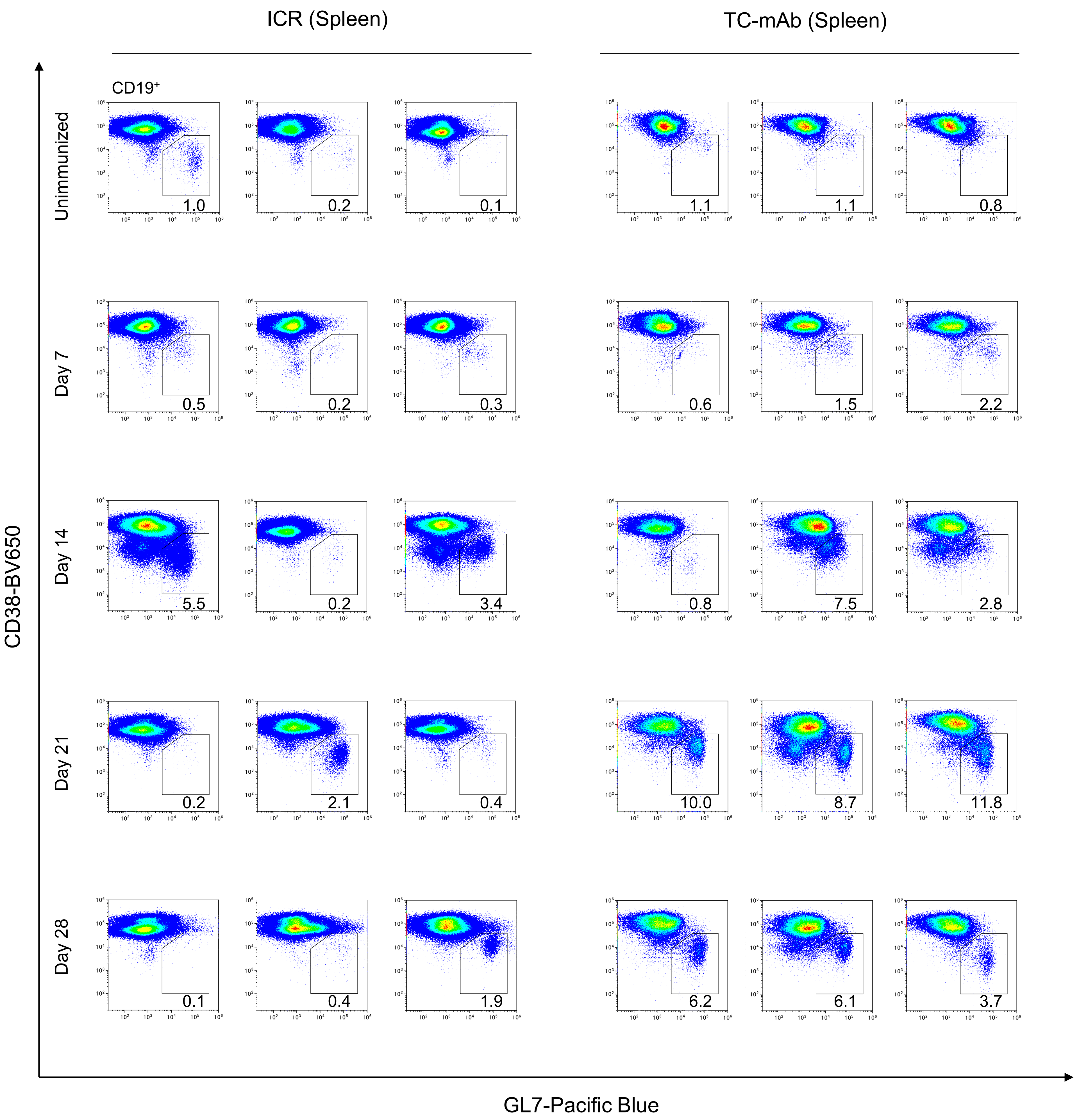

Supplementary Figure 28| Representation of flow cytometry in germinal centre (GC) B cells in spleen cells.
The distribution of GC B cells ($CD38^{lo/-}GL7^{+}$) in the spleen B cells ($CD19^{+}$) of ICR (n=3) and TC-mAb mice (n=3) before and after immunization with OVA. The spleen cells were collected from unimmunized mice and immunized mice at 7, 14, 21, and 28 days after immunization. The data collected from ICR mice (left side panels) and TC-mAb mice (right side panels) are indicated, respectively. The percentage of B cell subsets are represented in the GC B in the spleen. The statistical graph of the percentages of B cell subsets is presented on Figure 8b.

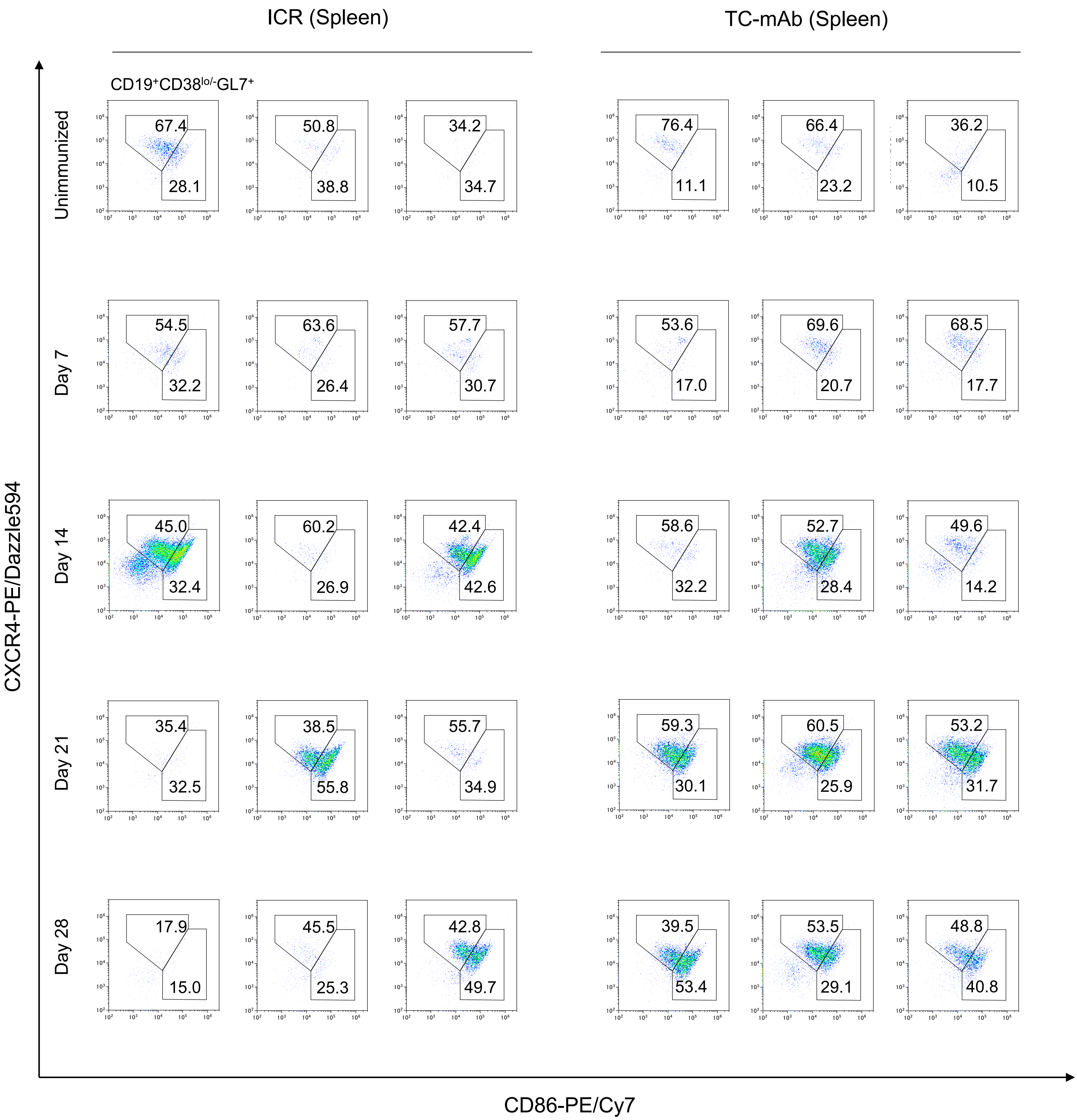

Supplementary Figure 29| Representation of flow cytometry in dark zone (DZ) and light zone (LZ) in germinal centre (GC) B cells of spleen.

The distribution of DZ ($CXCR4^{hi}CD86^{lo}$) and LZ ($CXCR4^{lo}CD86^{hi}$) in the spleen GC B ($CD19^{+}CD38^{lo/-}GL7^{+}$) of ICR (n=3) and TC-mAb mice (n=3) before and after immunization with OVA. The spleen cells were collected from unimmunized mice and immunized mice at 7, 14, 21, and 28 days after immunization. The data collected from ICR mice (left side panels) and TC-mAb mice (right side panels) are indicated, respectively. The percentage of B cell subsets are represented in the DZ and LZ in the GC B cells of spleen. The statistical graph of the ratios of dark zone and light zone B cell subsets is presented on Figure 8c.

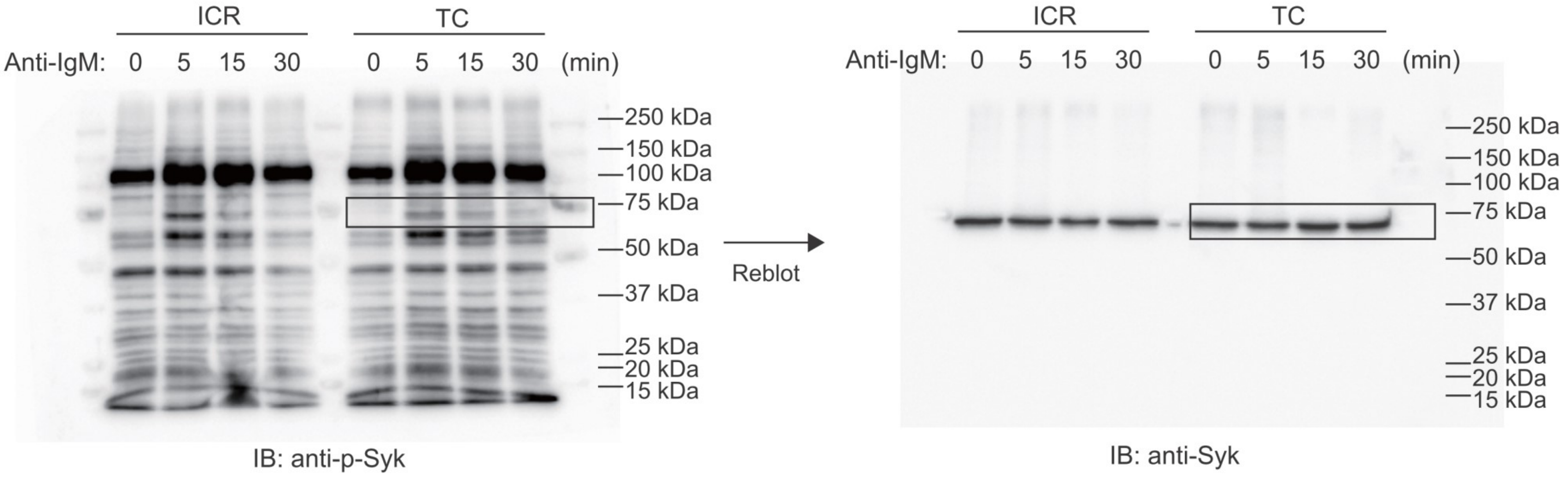

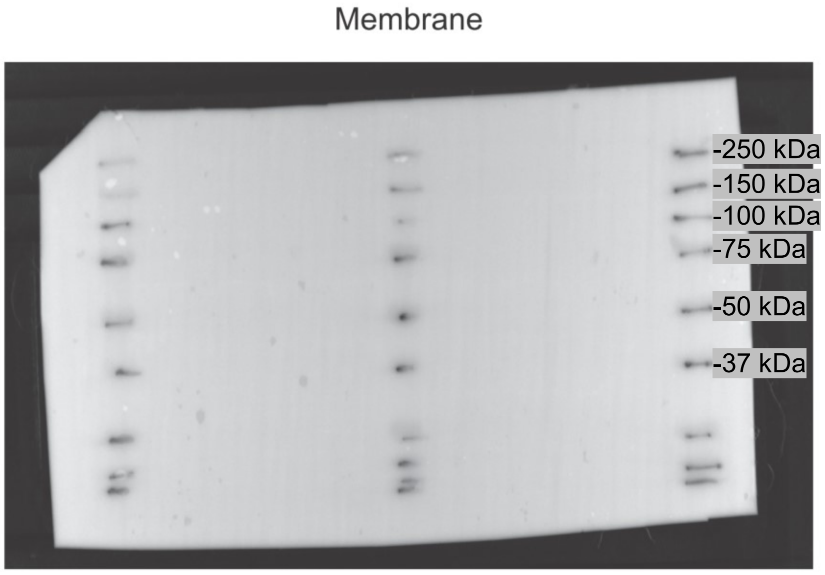

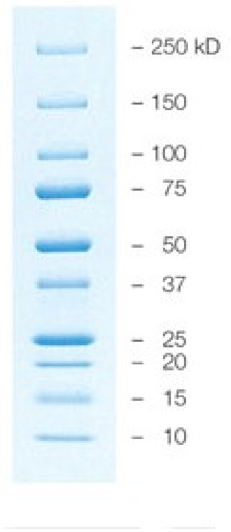

MW marker used for Western Blots:
Bio-Rad Precision Plus Protein All Blue Standards

Supplementary Figure 30| B cell stimulation analysis using spleen tyrosine kinase (Syk) activation.
Western blotting of p-Syk (Tyr525/526) **(a)** and Syk **(b)** in $CD43^+$ B cells of spleen. The $CD43^+$ B cells were separated from ICR and TC-mAb mice using anti-CD43 magnetic beads (see Supplementary methods), and the purified B cells (1×10^7 cells/ml) were stimulated with 10 μg/mL goat anti-mouse IgM F(ab)′2 or 10 μg/mL goat anti-human F(ab)′2, respectively, for indicated times. Phosphorylation status of Syk of B cells were assessed by western blotting of whole-cell lysates of stimulated B cells. Data are representative of at least two independent experiments. The first antibody was removed by the WB Stripping Solution (Nacalai Tesque). A second antigen-antibody reaction was conducted on the same blotting membrane. IB: immunoblot. Framed area indicated the cropped image as seen in the Figure 4i. **(c)** Transferred membrane after separating of protein by SDS-PAGE.

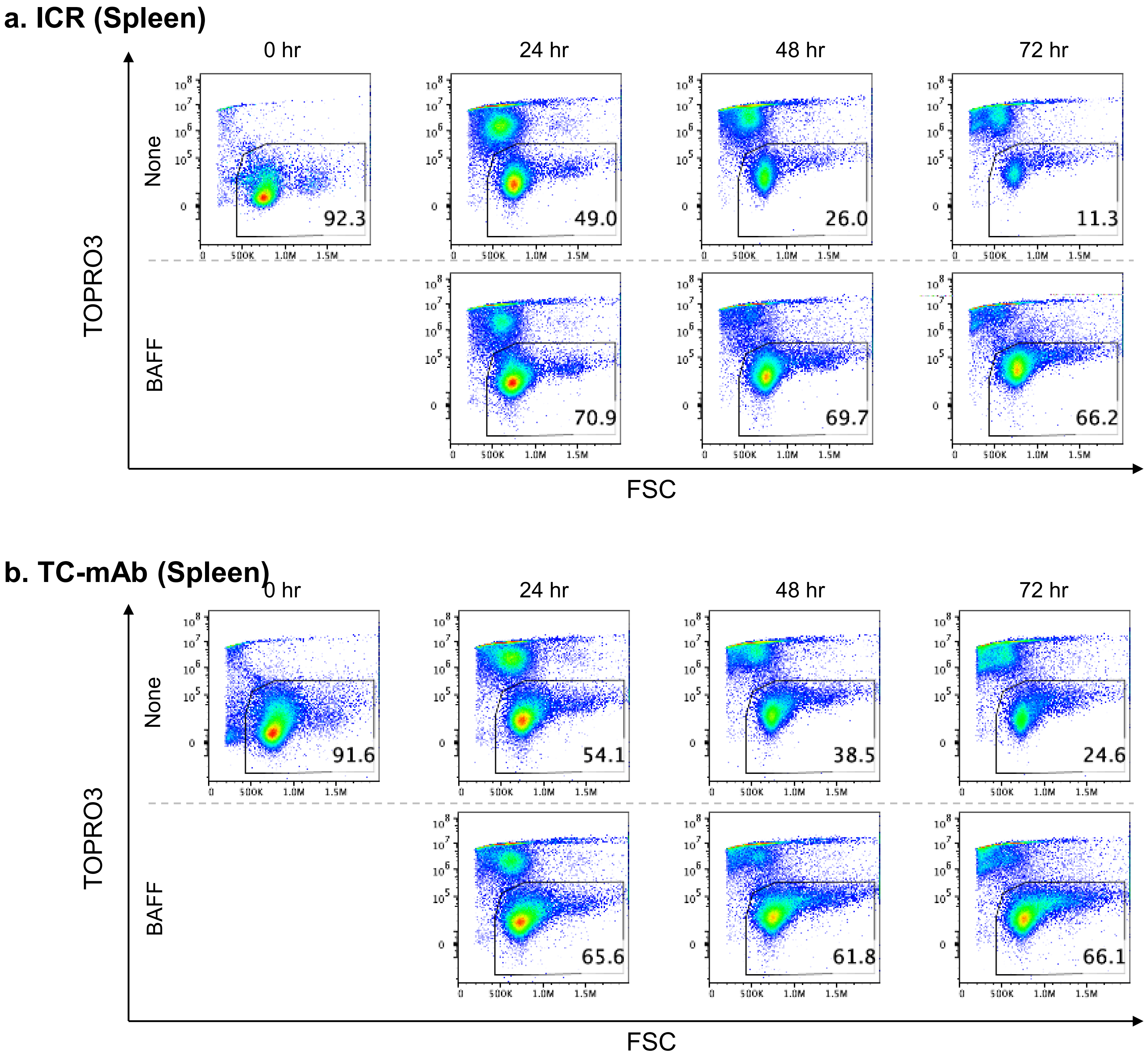

Supplementary Figure 31| B cell survival assay.
B cell survival assay using BAFF stimulation. The purified B cells from ICR **(a)** and TC-mAb mice **(b)** were cultured in the absence (None) or presence of 25 ng/ml BAFF for 24, 48, and 72 hrs. Staining with TOPRO3 was analysed by flow cytometry and percentages of TOPRO3-negative -gated cells (live cells) are shown. Results were pooled from two independent experiments.

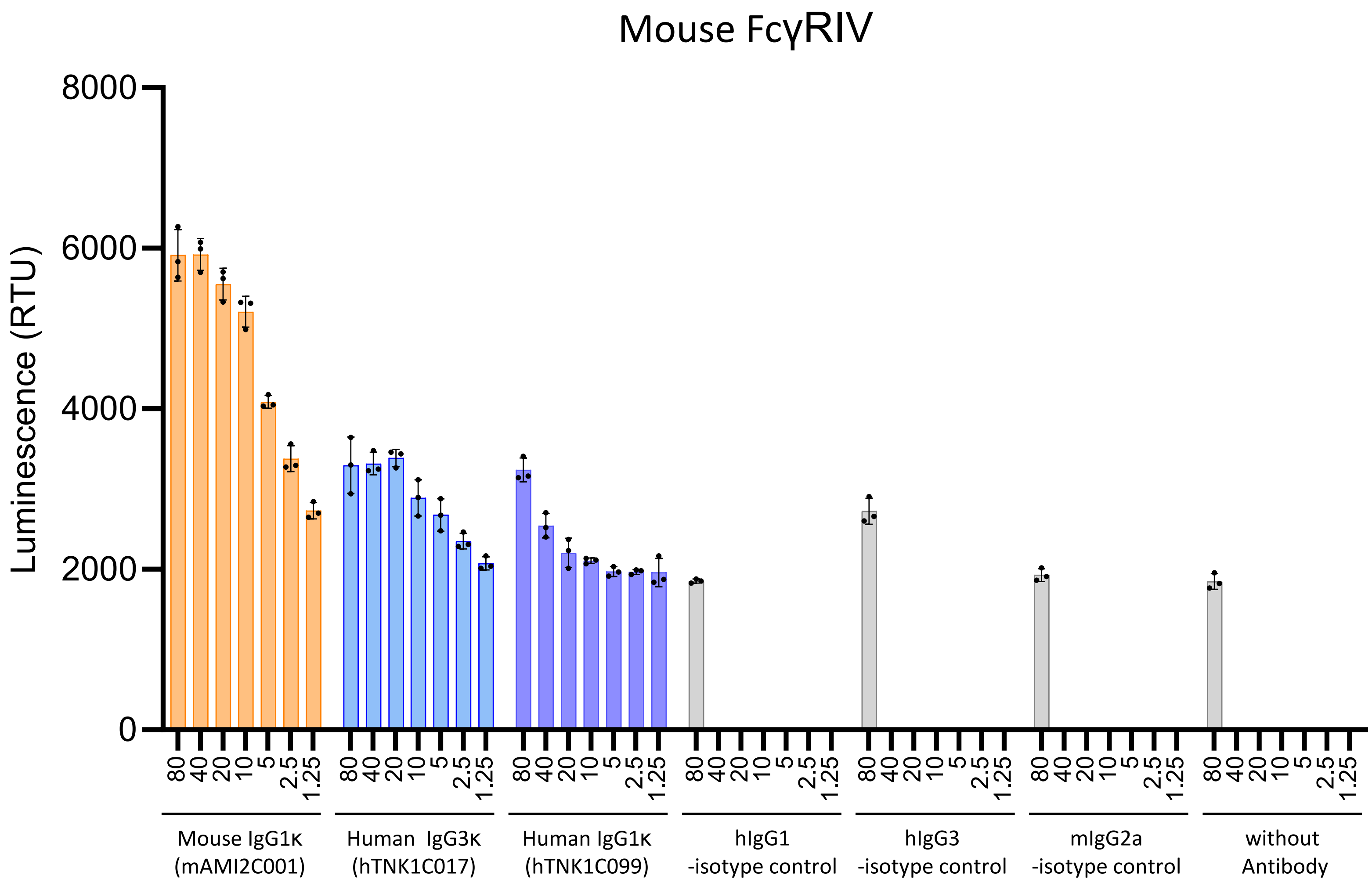

Supplementary Figure 32| Activation analysis of the mouse Fc gamma receptor IV by human and mouse mAbs.
The mouse Fc gamma RIV reporter bioassay to reveal activation of mouse receptor signalling by the obtained fully human mAbs. The obtained mAbs against AMIGO2 such as mAMI2C001 (mouse IgG1κ), hTNK1C017 (human IgG3κ), and hTNK1C099 (human IgG1κ) were mixed with the specific antigen (Trx-AMIGO2-Ig) and anti-His Tag magnetic beads to form immune complexes, respectively. The respective immune complexes prepared using the indicated concentrations of mAb were mixed with mFc gamma receptor IV effector cells and incubated at 37° C for 6 hrs, and then luminescence was measured. The isotype control of human IgG1, IgG3, and mouse IgG2a were used as negative controls. Three independent repetitions of the experiment are performed, with each point representing a data point. For the isotype control and no primary antibody groups, concentrations below 40 have not been performed.
Human Fc gamma RIIIa is known as the activating receptor and high affinity against IgG1, 3, and 4, respectively (Frontiers in Immunology 10:2968DOI:10.3389/fimmu.2019.02968), and its counterpart is thought to be as the mouse Fc gamma RIV (Trends in Immunology, June 2015, Vol. 36, No. 6). The binding analysis of recombinant human and mouse Fc gamma receptors also indicate human IgG1 and IgG3 significantly bind to mouse Fc gamma RIV (MABS, 2017, VOL9, No.5, p.767-773). Consisting with these reports, the immune complex of both mouse and human AMIGO2-specific mAbs elicited the signal of FcgRIV in dose-dependent manner although there is a difference in strength.

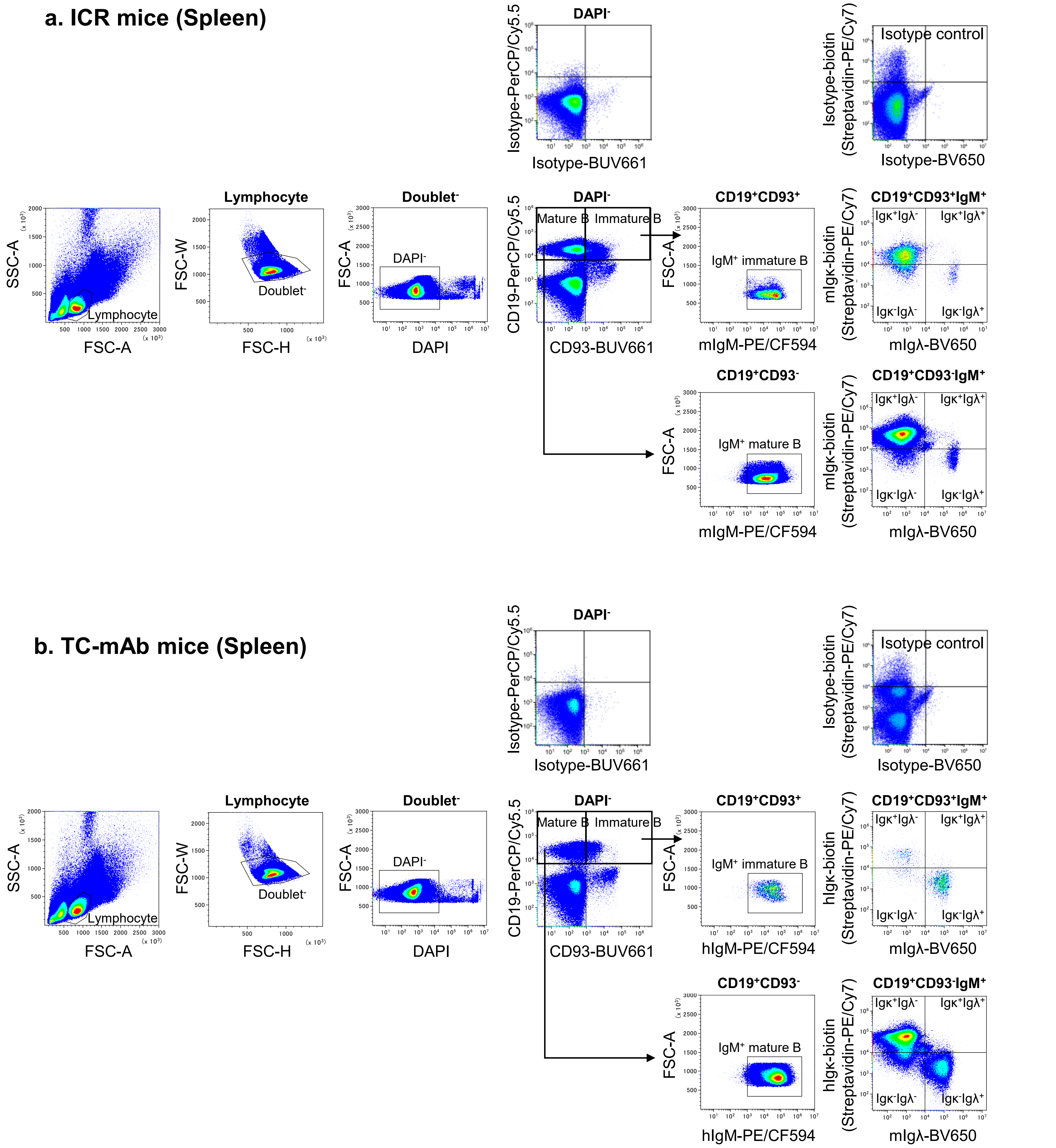

Supplementary Figure 33| Flow cytometry identification of human Igκ and mouse Igλ expressing B cells.
Flow cytometry gating strategies for human Igκ and mouse Igλ expressing B cells in spleen cells of ICR **(a)** and TC-mAb mice **(b).** Dot plots stained with fluorescence-labelled isotype controls (Supplementary Table 8) are shown at the top of each dot plot such as CD93 and CD19, mIgλ and mIgκ, and mIgλ and hIgκ, and are used to detect positive subsets.

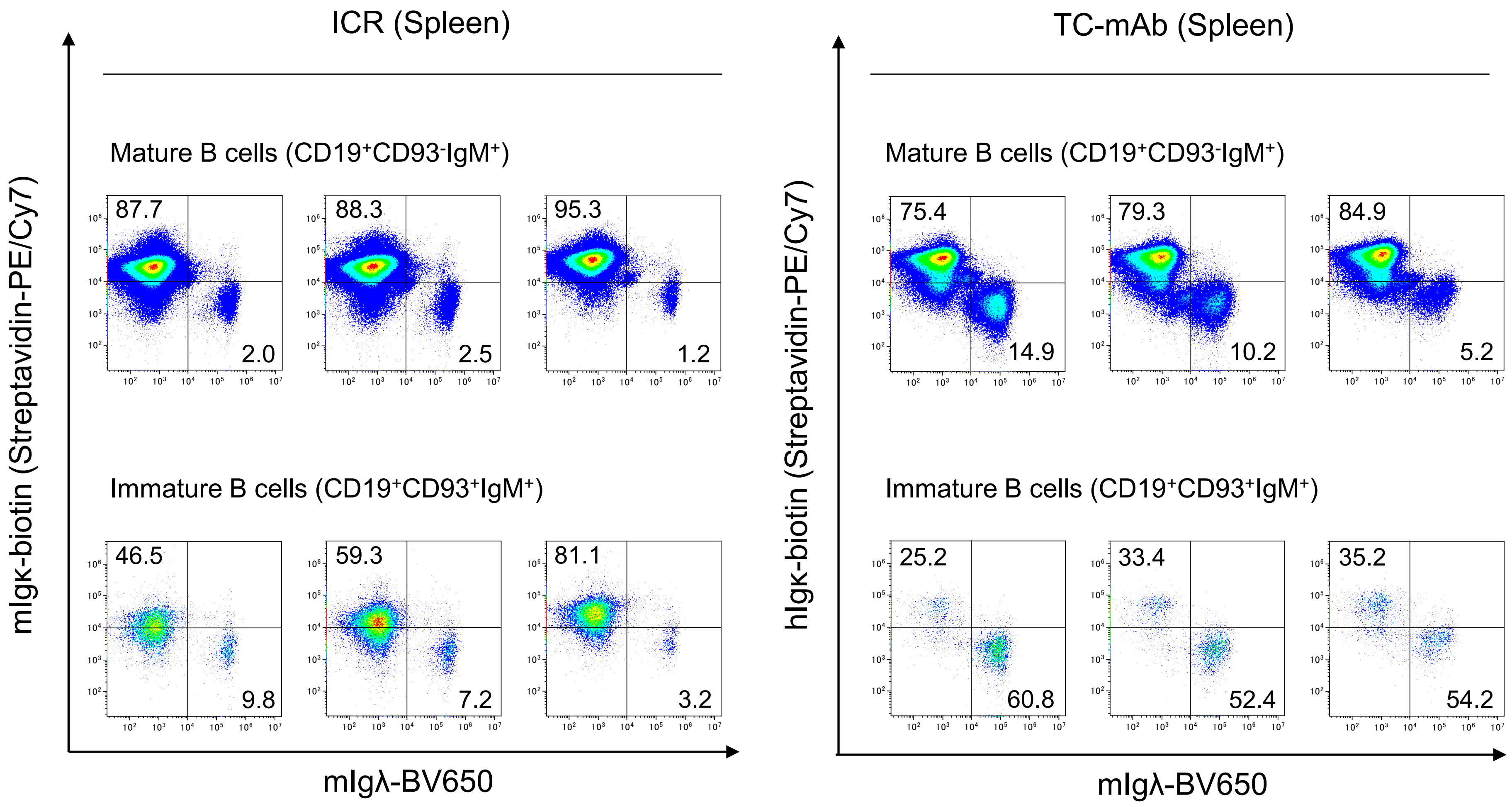

Supplementary Figure 34| Representation of flow cytometry in of human Igκ and mouse Igλ expressing B cells.
The distribution of human Igκ and mouse Igλ expressing B cells in the IgM$^+$ spleen mature (CD19$^+$CD93$^-$IgM$^+$) and immature (CD19$^+$CD93$^+$IgM$^+$) B cells of 13 weeks-age ICR (n=3) and TC-mAb mice (n=3). The percentage of B cell subsets are represented in human Igκ and mouse Igλ expressing B cells. The data collected from ICR mice (left side panels) and TC-mAb mice (right side panels) are indicated, respectively. The populations of mature B cells (upper) and immature B cells (lower) are represented.

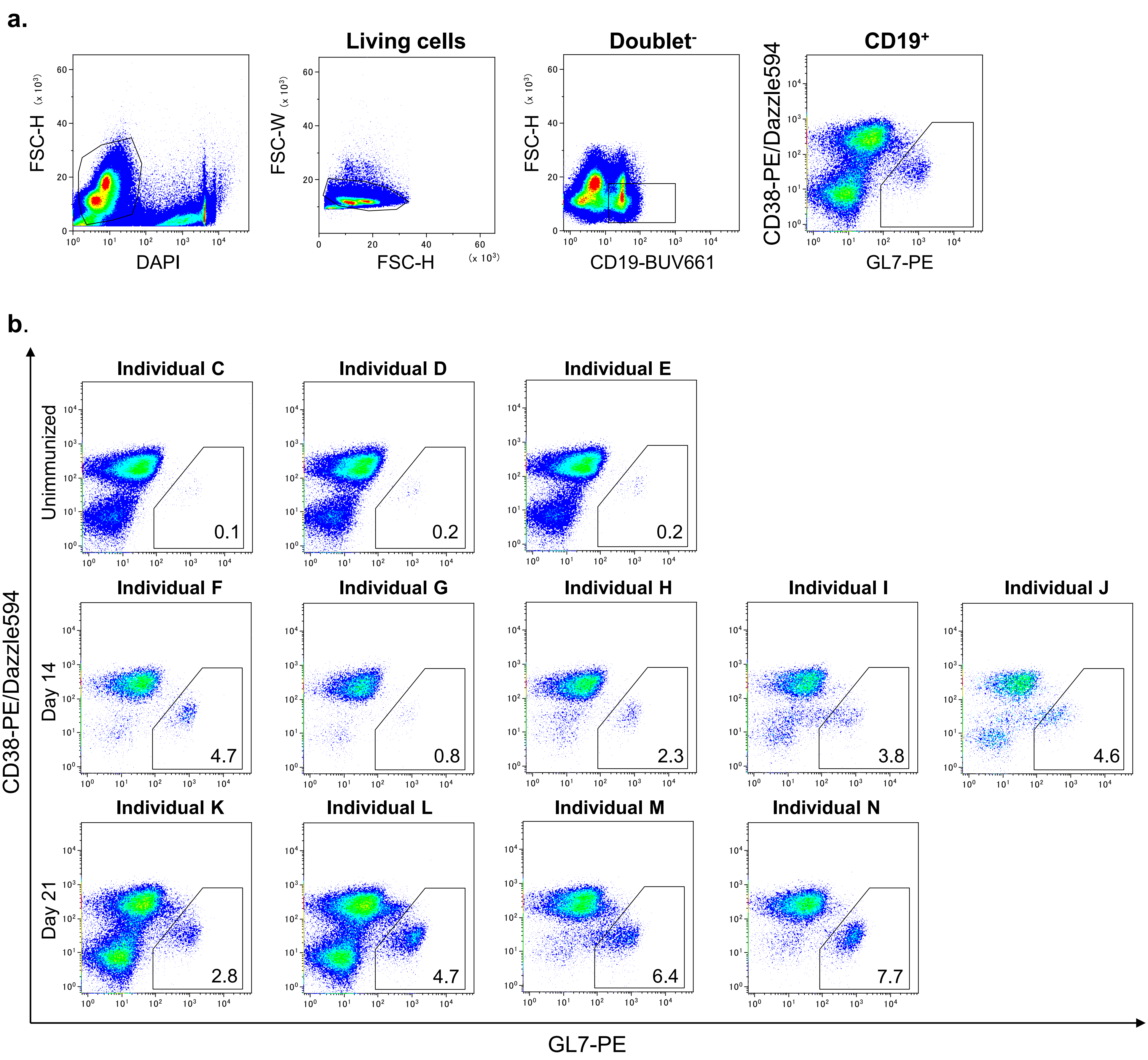

Table| RNA extraction of germinal centre B cells from TC-mAb mice

Individuals	F	G	H	I	J	K	L	M	N
Day after immunization			14				21		
Sorted cells (×10^5)	1.02	0.14	0.45	1.03	0.84	1.16	2.99	2.16	1.39
RNA concentration (ng/µl)	47.3	36.9	35.0	27.1	21.8	43.2	56.9	37.0	24.7
RNA integrity number	9.3	7.0	9.4	9.5	9.6	9.6	9.6	9.5	9.6

Supplementary Figure 35| Flow cytometry identification of germinal centre (GC) B cells in spleen cells of TC-mAb mice. Flow cytometry gating strategies to collect GC B cells after immunization with OVA (**a**) and the sorted fractions of GC B cells (CD19$^+$CD38$^{lo/-}$GL7$^+$) of respective TC-mAb mice at the time point after immunization with OVA (**b**). The number of days that have passed since immunization is indicated on the left side of panels. The table indicates the number of separated cells as GC B cell fraction using cell sorter. Total RNA concentration and RNA integrity number was measured by 2100 Bioanalyzer (Agilent, Santa Clara ,CA, USA). Equal amounts of purified total RNA samples were mixed to prepare pool samples on days 14 and 21.

a. Germinal centre B cells of the immunized TC-mAb (Spleen)

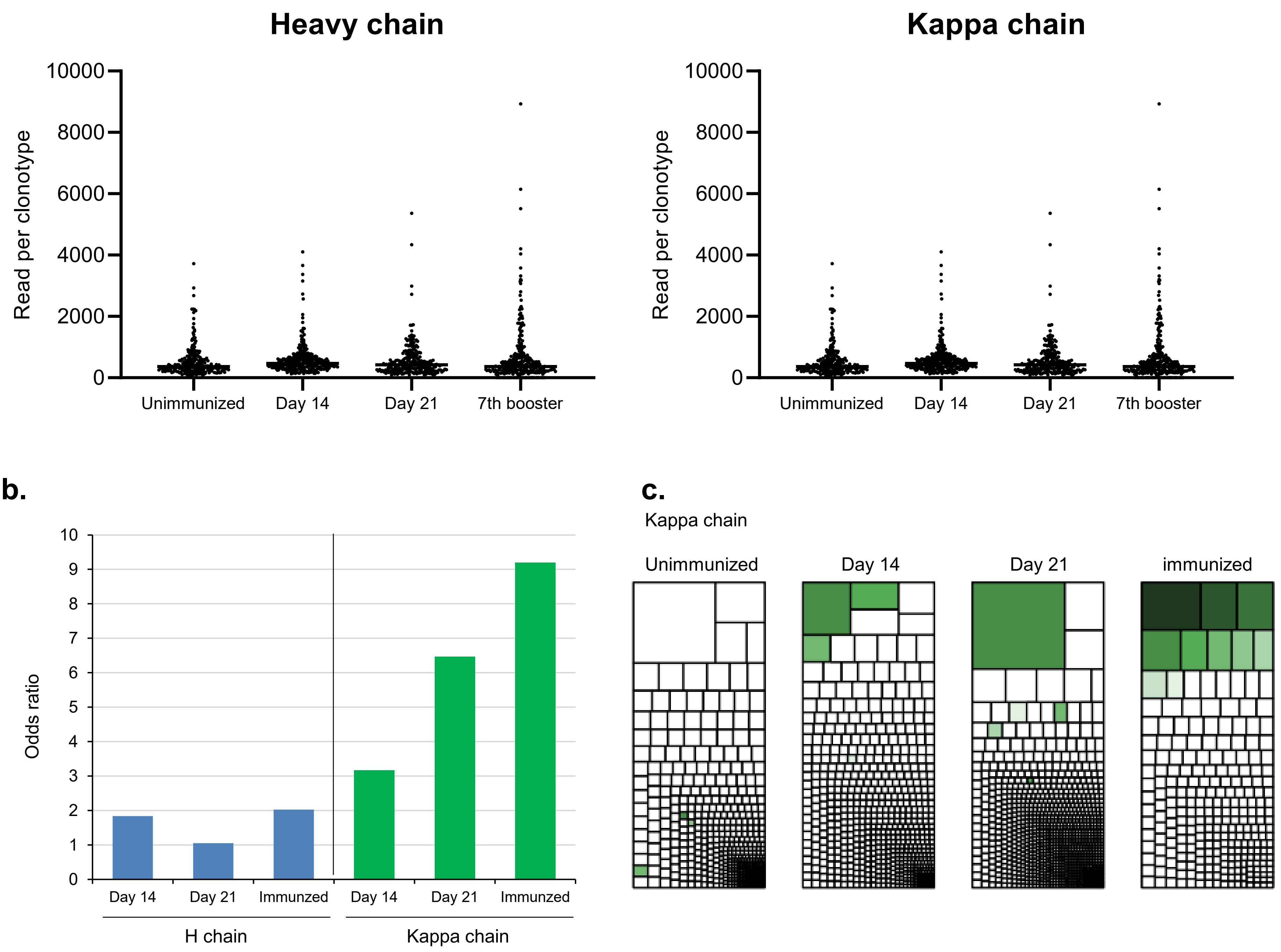

Supplementary Figure 36| Analysis of clonal expansion in germinal centre B of immunized TC-mAb mice. Analysis of expanded clonotypes in OVA-immunized TC-mAb mice. Heavy and kappa chain repertoire of the pooled GC B cells of day 14 (individual F-J, Supplementary Fig. 34) and day 21 (individual K-N) were analysed by NGS. The IgBlast-annotated reads were collated into data sets for subsequent analyses. The clonotypes were identified by the CDR3 amino acid sequence. **(a)** Beeswarm plot of the annotated reads for each clonotype. We analysed the number of reads for each clonotype in H and kappa chain which changes with the course of immunization (unimmunized (pool of five mice), day 14 (pool of five mice), day 21 (pool of four mice), and immunized (one individual)). In this figure, the y-axis shows the number of reads per clonotype which corresponds to the size of clonotype. Each dot point represents the group of “reads per clonotype” categorized with binning size of 1 read resolution. As immunization progressed, an increased in clonotype with a large number of reads was observed. **(b)** Focusing on the diversity of H and kappa chain, the degree of accumulation of somatic hyper mutation (SHM) were estimated by calculating odds score (see Methods section). The accumulation of SHM was clearly observed in kappa chain. **(c)** Mosaic plot of clonotypes. Clonotype was clustered by uniqueness of CDR3 amino acid sequences. The size of square area of each clonotype is proportional to the number of clones in the clonotype. Clone expansion was analysed as an expanded clones with more than 100 reads only in the immunized sample and less than 100 reads in the unimmunized sample. For the top 10 clonotypes of immunized sample, green gradation colors were assigned and the same coloring was used for the identical clonotype between the unimmunized, day 14, and day 21 samples. The same data set used in Fig. 4e and Supplementary Fig. 10 was used for the analysis of immunized sample. It was shown that some of the expanded clonotypes observed in the immunized sample were also expanded on day 14 and day 21.

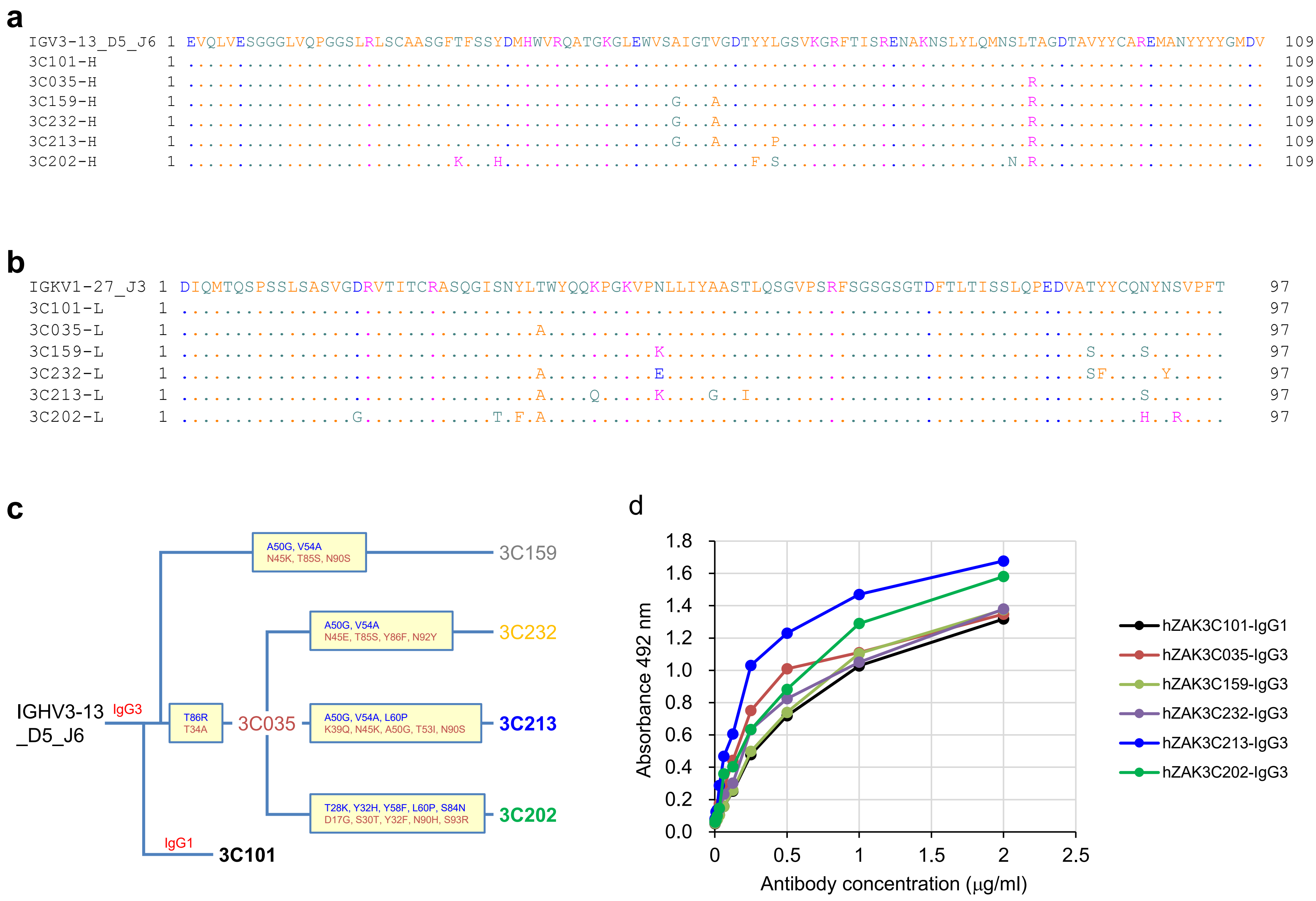

Supplementary Figure 37| Analysis of V region sequences and titres of EpCAM-specific mAbs.
The V region sequence and titres of anti-EpCAM mAbs obtained from TC-mAb mice. Alignment of VH **(a)** and VK **(b)** amino acid sequences from six anti-EpCAM mAbs of the same clone lineage. Amino acids that differ from the common germline VH (IGHV3-13|D5|J6) are indicated on the top, and the identical residues are denoted by a dash. **(c)** Dendrogram of the sequence relationship from germline for each of six mAbs. The changed amino acids are indicated in boxes. **(d)** Affinity against EpCAM antigen (Trx-EpEX) was determined by ELISA at a sequential dilution concentration of 2.0-0.002 μg/ml).
The 3C101 contains germline sequence (IGV3-13|D5|J6 and IGKV1-27|J3) in VH and VL but was class-switched from IgM to IgG1. Sequential occurrence of somatic hypermutation(s) from 3C101 to 3C035 and others enhanced their affinity against their antigen (Trx-EpEX), leading to estimate a clonal expansion of antigen-specific mAb producing B cells by immunization in TC-mAb mice .

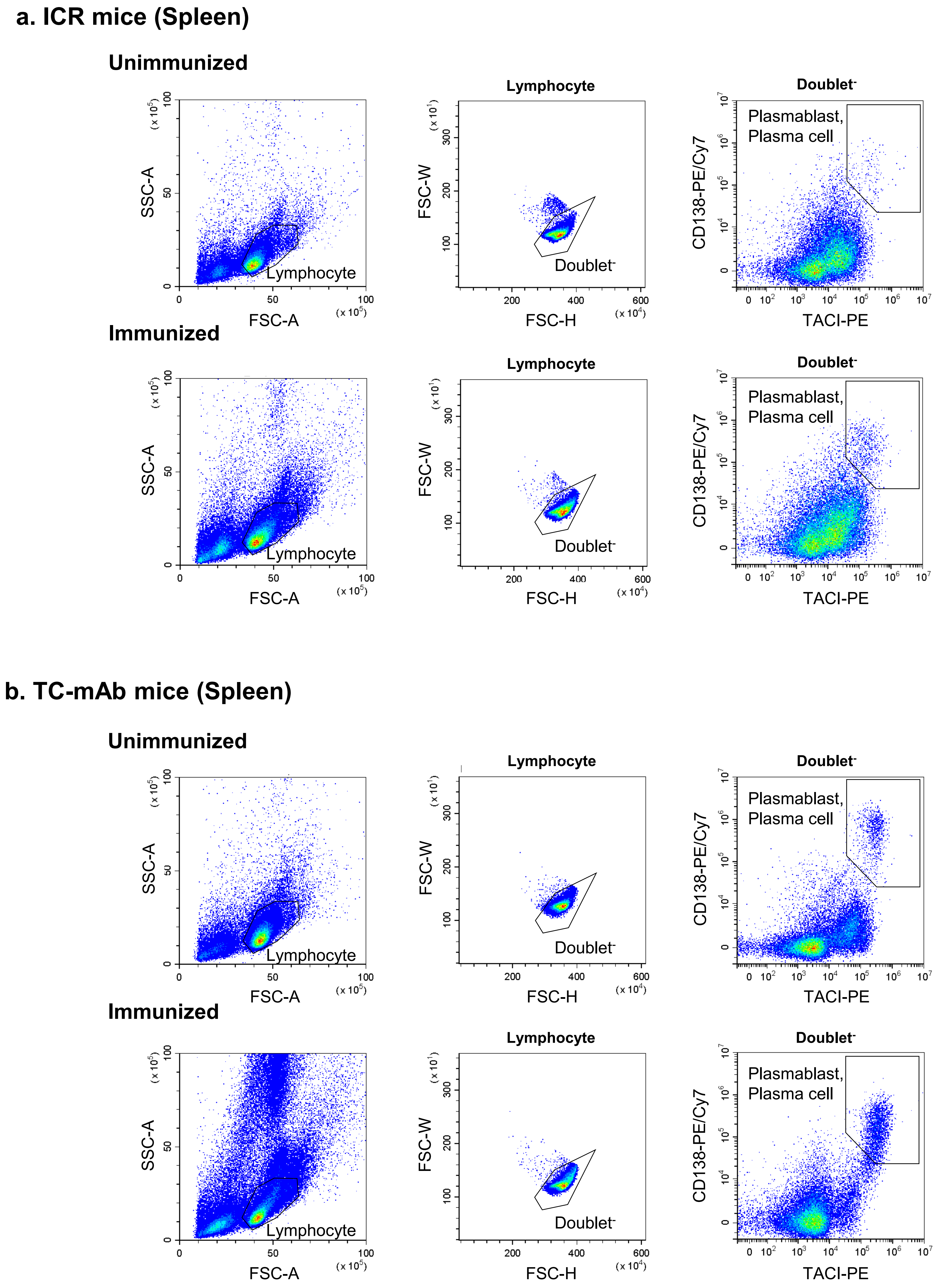

Supplementary Figure 38| Flow cytometry identification of plasmablasts/plasma cells

Flow cytometry gating strategies for in spleen cells of plasmablasts/plasma cells (CD138^{+}TACI^{+}) in spleen cells of ICR mice (**a**) and TC-mAb mice (**b**). The same of CD138-TACI panels are presented on Figure 9b.

Plasmablasts/Plasma cells (Spleen)

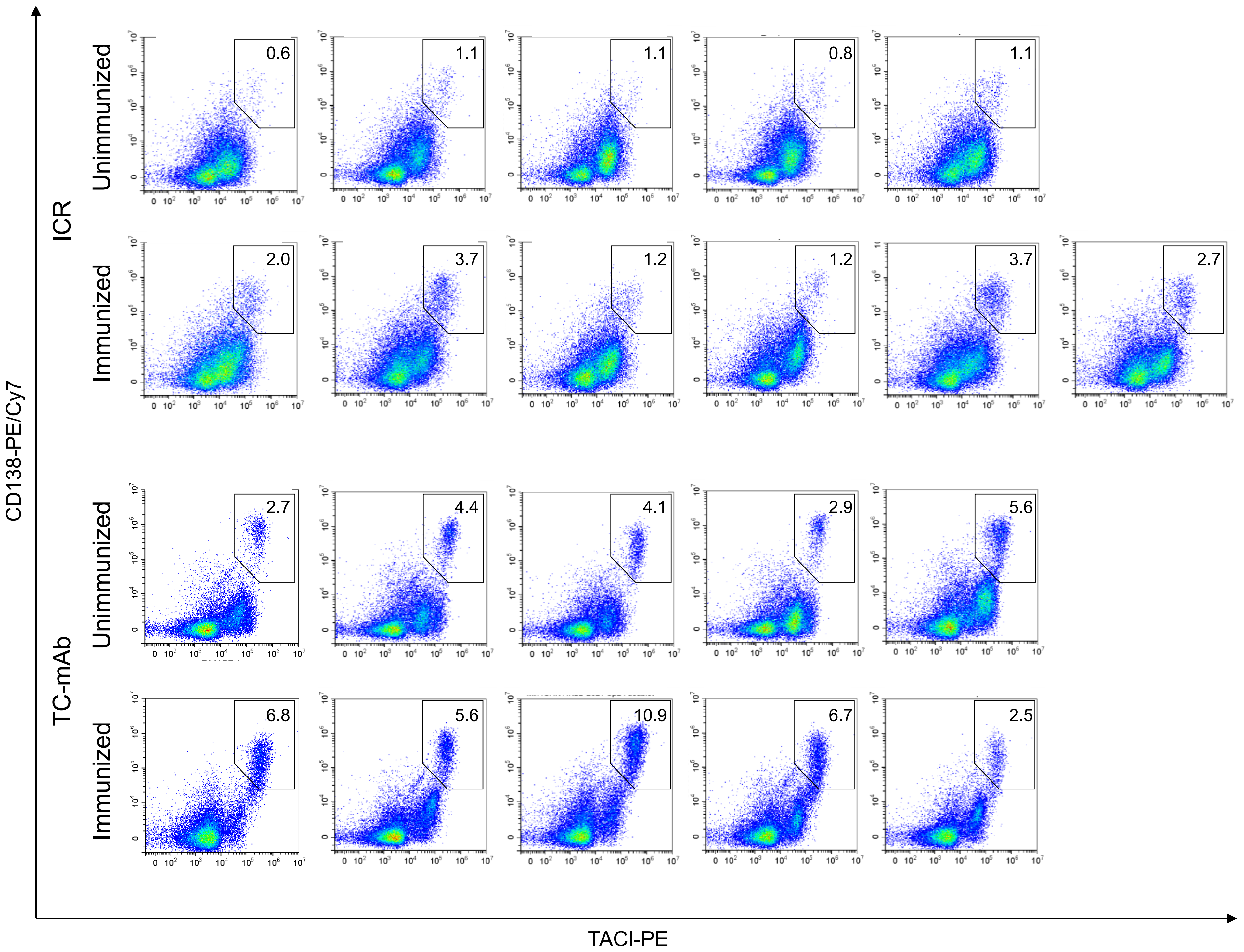

Supplementary Figure 39| Representation of flow cytometry in plasmablasts/plasma cells in the spleen cells.
The compartments of lymphocyte, plasmablasts/plasma cells ($CD138^{+}TACI^{+)}$ B cells in the spleen of approximately 21 weeks-age ICR mice (unimmunized mice,n=5; immunized mice, n=6) and TC-mAb mice (n=5). Numbers in the flow cytometry results indicate the percentage of each B cell subset(s).

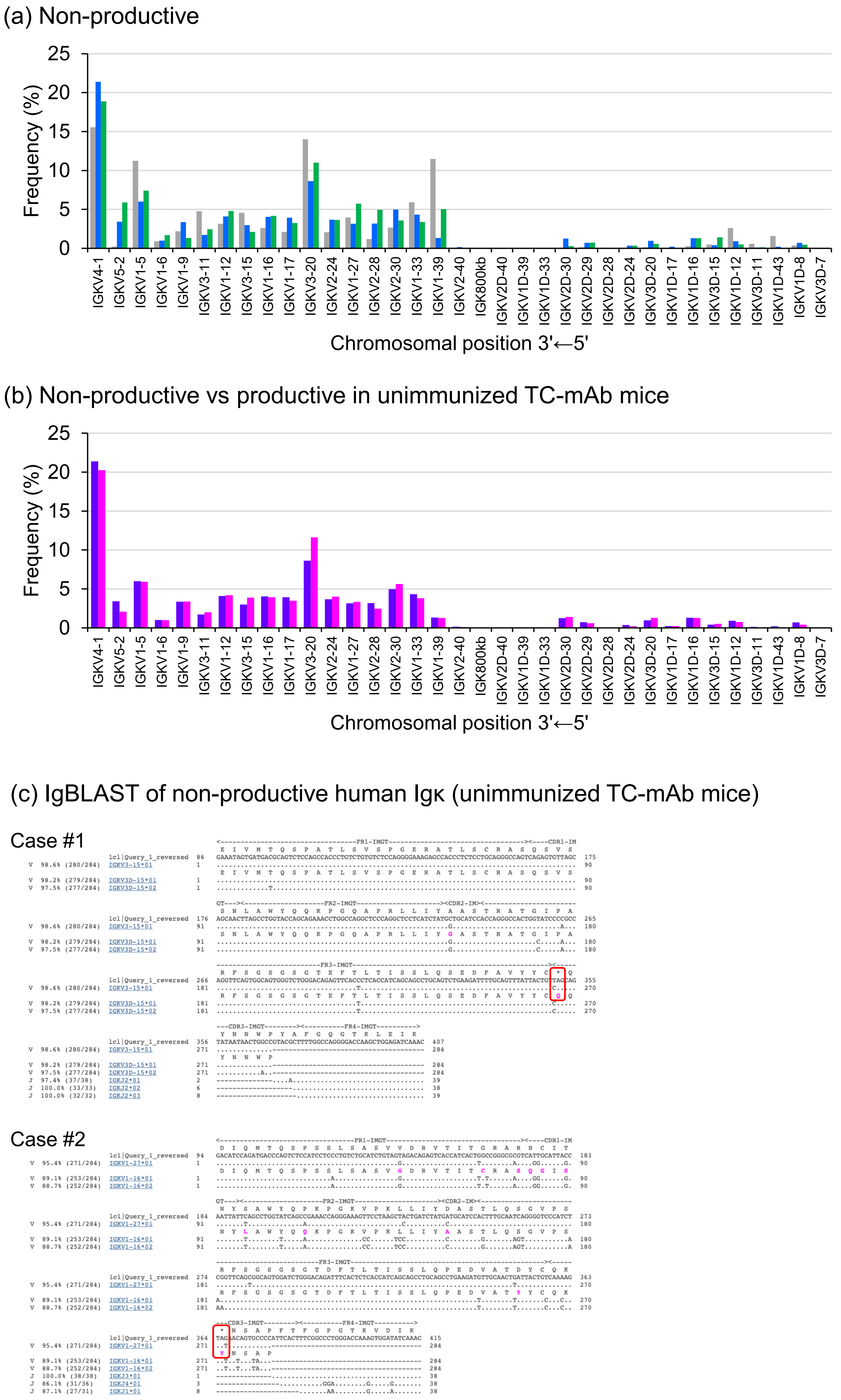

Supplementary Figure 40| Repertoire analysis of non-productive human Igκ.
(a) Frequency usage of V segments in non-productive reads of human Igκ. (b) Comparison of V segment usage between non-productive and productive reads in unimmunized TC-mAb mice. (c) Nucleotide and amino acid sequences of non-productive human Igκ. The stop codon is indicated by a red box. This figure is included in the response to a question from Reviewer 2 in the second revised revision.

Supplementary Table 1| Data collection of NGS.						
**Species**	**TC-mAb mice**				**hPBMCs**	
**Sample collection**	Unimmunized<br>*n=5*		Immunized<br>*n=1*		Healthy donors (Asians)<br>426 male/female, ages: 18-54	
**Analysed chain**	IgG and IgM	Igκ	IgG and IgM	Igκ	IgG and IgM	Igκ
**Original reads**	2,811,364	1,398,612	3,567,368	2,540,916	1,662,560	1,483,520
**Merged reads**	1,256,816	782,060	1,863,712	1,845,272	627,292	1,082,276
**Annotated reads**	312,705	195,281	465,431	460,851	156,798	270,485
**CDR3 reads**	280,739	188,313	450,749	438,134	139,745	262,936
**Productive reads (%)**	263,500 (93.9)	180,629 (95.9)	418,032 (92.7)	419,601(95.8)	127,103 (91.0)	248,872 (94.7)

Merged reads represented the number of NGS reads recovered from preprocessing with merge paired-end reads of original reads. Annotated reads and CDR3 reads were determined as human Ig-sequence identified by IgBlast. Productive reads (%) indicated the percentage of productive amino acid sequence with in-frame and contained no stop codon.

Heavy chain

Supplementary Table 2| Frequency usage of V-gene segments.

	hPBMC	Unimmunized	Immunized
IGHV6-1	0.53	4.39	9.02
IGHV1-2	2.42	1.35	0.41
IGHV1-3	2.96	1.49	2.00
IGHV4-4	1.77	0.04	0.05
IGHV7-4	2.44	4.07	6.16
IGHV2-5	4.02	9.25	7.46
IGHV3-7	5.34	1.55	1.84
IGHV1-8	0.45	7.39	3.80
IGHV3-9	5.09	6.36	7.25
IGHV3-11	2.45	2.45	1.28
IGHV3-13	1.18	1.41	0.68
IGHV3-15	2.40	2.29	1.79
IGHV1-18	5.74	3.61	6.79
IGHV3-20	0.02	0.50	0.28
IGHV3-21	2.32	2.36	2.26
IGHV3-23	12.27	8.34	2.52
IGHV1-24	0.53	6.70	0.34
IGHV2-26	0.98	3.07	0.44
IGHV4-28	0.21	0.07	0.18
IGHV3-30	5.32	0.69	0.38
IGHV4-31	1.19	0.05	0.01
IGHV3-33	2.84	7.98	3.95
IGHV4-34	4.21	4.08	4.14
IGHV4-39	2.41	6.94	6.85
IGHV3-43	0.90	0.27	0.37
IGHV1-45	0.01	0.01	0.03
IGHV1-46	1.00	0.45	1.06
IGHV3-48	4.82	2.80	3.79
IGHV3-49	0.89	0.16	0.06
IGHV5-51	2.80	0.69	4.90
IGHV3-53	1.38	0.87	0.61
IGHV1-58	0.39	0.05	0.01
IGHV4-59	4.92	3.83	7.81
IGHV4-61	1.15	0.44	2.58
IGHV3-64	0.72	0.04	0.01
IGHV3-66	0.95	0.08	0.08
IGHV1-69	4.42	1.98	1.67
IGHV2-70	1.41	0.75	4.97
IGHV3-72	0.74	0.08	0.07
IGHV3-73	0.49	0.41	0.20
IGHV3-74	2.46	0.50	1.83

Supplementary Table 2| Frequency usage of D-gene segments.

	hPBMC	Unimmunized	Immunized
IGHD1-1	2.49	1.47	0.55
IGHD2-2	9.96	1.21	0.78
IGHD3-3	2.73	0.45	0.79
IGHD4-4	0.00	0.00	0.00
IGHD5-5	0.00	0.00	0.00
IGHD6-6	3.03	0.06	0.09
IGHD1-7	0.88	0.22	0.14
IGHD2-8	0.97	0.20	0.10
IGHD3-9	5.41	8.93	6.54
IGHD3-10	11.79	33.46	30.38
IGHD4-11	0.37	0.11	0.24
IGHD5-12	3.81	2.47	2.56
IGHD6-13	9.84	11.96	7.69
IGHD1-14	1.98	1.96	1.26
IGHD2-15	4.50	1.78	0.70
IGHD3-16	3.82	1.87	1.35
IGHD4-17	4.27	2.28	7.05
IGHD5-18	2.92	0.94	2.02
IGHD6-19	6.30	16.77	21.08
IGHD1-20	0.37	0.29	0.24
IGHD2-21	2.23	0.86	2.29
IGHD3-22	7.30	1.29	2.60
IGHD4-23	2.44	1.42	1.67
IGHD5-24	4.64	2.21	0.62
IGHD6-25	0.20	0.08	0.13
IGHD1-26	7.48	5.57	8.01
IGHD7-27	0.24	2.14	1.14

Supplementary Table 2| Frequency usage of J-gene segments.

	hPBMC	Unimmunized	Immunized
IGHJ1	0.68	3.02	3.17
IGHJ2	3.33	5.09	5.10
IGHJ3	4.98	9.96	10.76
IGHJ4	42.61	40.72	52.35
IGHJ5	6.87	5.74	10.47
IGHJ6	41.53	35.48	18.15

(continued)

Kappa chain

Supplementary Table 2| Frequency usage of V-gene segments.		hPBMC	Unimmunized	Immunized
Proximal cluster	IGKV4-1	12.68	22.26	16.41
	IGKV5-2	0.60	2.42	5.66
	IGKV1-5	11.92	6.52	7.50
	IGKV1-6	1.12	1.09	1.71
	IGKV1-9	2.93	3.70	1.22
	IGKV3-11	6.54	2.18	2.94
	IGKV1-12	3.59	4.62	4.59
	IGKV3-15	8.71	4.23	2.50
	IGKV1-16	3.34	4.32	4.25
	IGKV1-17	1.59	3.86	3.13
	IGKV3-20	18.47	12.57	14.56
	IGKV2-24	1.53	5.75	5.54
	IGKV1-27	2.37	3.65	5.86
	IGKV2-28	1.73	2.76	5.33
	IGKV2-30	3.10	6.21	3.56
	IGKV1-33	4.06	4.87	4.31
	IGKV1-39	13.05	1.41	4.99
	IGKV2-40	0.06	0.18	0.25
	800 kbp			
Distal cluster	IGKV2D-40	0.00	0.00	0.00
	IGKV1D-39	0.00	0.00	0.00
	IGKV1D-33	0.00	0.00	0.00
	IGKV2D-30	0.00	1.54	0.21
	IGKV2D-29	0.12	0.67	0.80
	IGKV2D-28	0.00	0.00	0.00
	IGKV2D-24	0.05	0.23	0.10
	IGKV3D-20	0.19	1.37	0.57
	IGKV1D-17	0.00	0.24	0.01
	IGKV1D-16	0.36	1.39	1.12
	IGKV3D-15	0.92	0.59	1.83
	IGKV1D-12	0.67	0.82	0.43
	IGKV3D-11	0.04	0.05	0.19
	IGKV1D-43	0.14	0.02	0.01
	IGKV1D-8	0.12	0.48	0.43
	IGKV3D-7	0.00	0.00	0.00

Supplementary Table 2| Frequency usage of J-gene segments.

Sample	hPBMC	Unimmunized	Immunized
IGKJ1	29.07	37.40	40.56
IGKJ2	29.79	23.88	21.83
IGKJ3	11.12	15.26	12.57
IGKJ4	22.47	18.91	19.13
IGKJ5	7.57	4.56	5.90

The relative usage of human variable regions in hPBMCs and TC-mAb mice was indicated. The genes segments annotated as an open reading frame or pseudogene[18,19] were excluded.

Supplementary Table 3| Data Shannon-Weaver Index (H′).

	hPBMCs	Immunized	Unimmunized
**HV**	3.371	3.129	3.143
**HD**	3.018	2.305	2.300
**HJ**	1.386	1.209	1.384
**LV**	2.594	2.910	3.051
**LJ**	1.495	1.442	1.452

The Shannon-Weaver diversity indexes were represented based on communication theory. The indexes were calculated using productive reads indicated in Supplementary Table 1.

Supplementary Table 4| The 20 most frequently used clone lineages in heavy chain.

				Annotated Reads			SHM%		Average length of CDR3	
Clone Lineage	V	D	J	Immunized	Unimmunized	Fold Change	Immunized	Unimmunized	Immunized	Unimmunized
CLH001	IGHV4-59*01	IGHD3-10*01	IGHJ4*02	11108	591	18.8	39.3	29.1	10.8	13.6
CLH002	IGHV6-1*01	IGHD3-10*01	IGHJ6*02	8352	741	11.3	43.6	55.9	16.1	16.6
CLH003	IGHV7-4-1*02	IGHD6-19*01	IGHJ4*02	7235	340	21.3	31.7	31.8	12.9	11.1
CLH004	IGHV6-1*01	IGHD6-19*01	IGHJ4*02	6442	1144	5.6	47.9	40.2	9.8	11.7
CLH005	IGHV3-9*01	IGHD4-17*01	IGHJ6*02	6797	68	100.0	42.9	45.6	16.1	16.8
CLH006	IGHV2-5*02	IGHD6-19*01	IGHJ4*02	5603	894	6.3	18.5	20.4	14.4	12.8
CLH007	IGHV4-39*01	IGHD1-26*01	IGHJ4*02	5439	278	19.6	84.3	23.0	10.0	9.7
CLH008	IGHV6-1*01	IGHD3-10*01	IGHJ4*02	4829	596	8.1	44.0	45.1	13.8	12.6
CLH009	IGHV2-70*01	IGHD3-10*01	IGHJ6*02	5050	159	31.8	23.9	27.0	18.6	17.8
CLH010	IGHV2-70*01	IGHD6-19*01	IGHJ6*02	4948	245	20.2	72.5	41.2	18.0	17.0
CLH011	IGHV1-18*01	IGHD1-26*01	IGHJ6*02	4742	179	26.5	38.4	38.5	16.8	17.1
CLH012	IGHV2-5*02	IGHD3-10*01	IGHJ4*02	4858	8594	0.6	59.1	16.3	15.8	12.6
CLH013	IGHV1-18*01	IGHD3-9*01	IGHJ6*02	3921	1237	3.2	41.7	38.7	21.4	19.2
CLH014	IGHV4-59*01	IGHD3-10*01	IGHJ6*02	2763	986	2.8	55.7	31.0	17.8	18.2
CLH015	IGHV1-18*01	IGHD5-12*01	IGHJ6*02	3461	200	17.3	35.1	82.0	18.0	18.1
CLH016	IGHV3-9*01	IGHD3-10*01	IGHJ6*02	2927	1532	1.9	64.4	61.3	17.6	18.3
CLH017	IGHV3-9*01	IGHD6-19*01	IGHJ6*02	1845	939	2.0	67.4	77.2	16.6	19.4
CLH018	IGHV3-33*01	IGHD6-19*01	IGHJ4*02	2610	1009	2.6	52.1	44.7	11.4	12.8
CLH019	IGHV2-5*02	IGHD5-18*01	IGHJ4*02	2799	166	16.9	30.8	18.1	12.9	11.8
CLH020	IGHV3-21*01	IGHD3-10*01	IGHJ6*02	2399	665	3.6	47.1	52.6	19.9	20.5
Average						16.0	47.0	41.0	15.4	15.4

Supplementary Table 4| The 20 most frequently used clone lineages in kappa chain.

				Annotated Reads			SHM%		Average length of CDR3	
Clone Lineage	V	D	J	Immunized	Unimmunized	Fold Change	Immunized	Unimmunized	Immunized	Unimmunized
CLL001	IGKV4-1*01	-	IGKJ1*01	37330	12723	2.9	29.0	35.2	9.0	9.0
CLL002	IGKV3-20*01	-	IGKJ1*01	28397	8806	3.2	70.4	52.9	8.3	9.0
CLL003	IGKV1-5*03	-	IGKJ1*01	17544	4416	4.0	41.6	28.1	8.8	8.6
CLL004	IGKV5-2*01	-	IGKJ2*01	13024	1354	9.6	40.0	33.8	9.0	9.1
CLL005	IGKV4-1*01	-	IGKJ2*01	12437	8426	1.5	41.8	25.6	8.8	9.0
CLL006	IGKV2-24*01	-	IGKJ1*01	10555	4171	2.5	53.6	34.5	8.6	9.0
CLL007	IGKV1-27*01	-	IGKJ1*01	11035	2504	4.4	52.3	45.3	9.0	9.0
CLL008	IGKV3-20*01	-	IGKJ2*01	10693	3618	3.0	71.6	46.5	8.9	8.9
CLL009	IGKV4-1*01	-	IGKJ4*01	8613	4254	2.0	52.1	33.6	9.0	9.0
CLL010	IGKV5-2*01	-	IGKJ1*01	8688	1566	5.5	62.7	33.1	8.4	8.4
CLL011	IGKV1-39*01	-	IGKJ4*01	8209	450	18.2	88.7	37.8	9.0	8.9
CLL012	IGKV1-27*01	-	IGKJ4*01	8017	970	8.3	92.6	55.4	9.0	9.0
CLL013	IGKV3-20*01	-	IGKJ4*01	6929	2694	2.6	82.9	31.6	8.7	8.6
CLL014	IGKV2-28*01	-	IGKJ1*01	5997	644	9.3	78.6	56.8	9.0	9.0
CLL015	IGKV3-20*01	-	IGKJ3*01	6552	1847	3.5	56.7	38.2	8.5	9.0
CLL016	IGKV1-12*01	-	IGKJ1*01	6561	882	7.4	86.5	49.2	9.1	9.0
CLL017	IGKV2-28*01	-	IGKJ3*01	5085	335	15.2	44.6	66.0	9.0	9.0
CLL018	IGKV1-5*03	-	IGKJ2*01	5565	2138	2.6	48.8	27.2	8.7	8.4
CLL019	IGKV1-12*01	-	IGKJ3*01	5049	1440	3.5	55.8	49.3	8.9	9.0
CLL020	IGKV2-30*02	-	IGKJ4*01	5013	1328	3.8	65.0	33.4	9.0	9.0
Average						5.7	60.8	40.7	8.8	8.9

Relative frequency of top 20 clone lineages (CLH001-020 of VH and CLL001-020 of VK) were summarized with the fold change of annotated reads, the average length of CDR3, and the percentage of SHM with at least two mutations in a variable region.

Supplementary Table 5| Serum Ig-levels in TC-mAb mice.			
	Average (μg/ml)	SD	Range
**Unimmunized TC-mAb mice**			
**Number of mice=17**			
**hIgμ**	518.1	242.2	270.6-1067.8
**hIgγ**	153.2	72.6	22.8-306.4
**hIgκ**	688.6	209.6	396.3-1095.2
**hIgα**	3.4	2.7	0.6-11.1
**hIgε**	0.2	0.3	0.0-1.3
**mIgμ**	21.3	7.1	9.2-36.1
**mIgγ**	11.5	11.2	0.9-45.7
**mIgκ**	29.7	9.3	20.6-57.8
**mIgλ**	4.6	1.7	2.0-8.6
**Number of mice=9**			
**hIgγ1**	82.2	59.3	17.8-176.0
**hIgγ2**	109.3	68.9	33.3-257.7
**hIgγ3**	4.6	3.8	0.7-11.6
**hIgγ4**	13.3	18.3	0.3-47.2
**Immunized TC-mAb mice**			
**Number of mice=21**			
**hIgμ**	2,097	1,396.0	485.5-5534.2
**hIgγ**	1,322	1,044.8	318.5-5070.4
**hIgκ**	4,122	2,980.9	923.0-14103.6
**Unimmunized ICR mice**			
**Number of mice=12**			
**mIgμ**	248.0	130.8	107.8-511.3
**mIgγ**	993.4	992.6	141.3-1779.8
**mIgκ**	637.0	546.5	124.6-1842.7
**OVA-immunized TC-mAb mice**			
**Number of mice=5**			
**hIgμ**	2,457	1,496.6	1281.8-5041.6
**hIgγ**	1,087	605.0	44.6-1573.8
**OVA-immunized ICR mice**			
**Number of mice=8**			
**mIgμ**	636.1	320.7	191.1-1208.9
**mIgγ**	12,835	3,679.0	3679.0-19597.5

Supplementary Table 6| Monoclonal antibody production in mice.			
**Mouse**	**TC-mAb**		**Balb/c**
	Individual A	Individual B	
**Immunization**	Primary and 4 boosters	Primary and 7 boosters	Primary and 6 boosters
**Lymphocytes (cells/mouse)**	1.87×10^{8}	2.40×10^{8}	1.91×10^{8}
**Lymphocytes/cell fusion (cells)**	0.9×10^{8}	0.9×10^{8}	1.4×10^{8}
**ELISA positive well after HAT selection (well)**	303	428	220
**ELISA positive well after second screening (clones)**	80	349	51
**Determination of subclass (clones)**	80	297	8
**Bound to native antigen (clones)**	20	52	8
**Cloned by limiting dilution (clones) (Success rate of cloning)**	20 (100%)	50 (96.1%)	8 (100%)

Production and screening steps of hybridoma cell lines producing anti-EpCAM mAbs are summarized. The numbers of hybridoma cells (clones) are indicated when Balb/c and TC-mAb mice were immunized with the Trx-EpEX protein as antigen. A portion of the harvested lymphocytes was used for cell fusion, as indicated in the 'lymphocytes/cell fusion' row. After selection with HAT medium, the antigen-positive wells, which contained a few of hybridoma cell colonies, were screened and hybridoma cells were picked from those wells. After a second screening to check the antigen-specific binding of Abs, positive hybridoma cells were analysed for subclass determination. After analysing the binding activity of Abs to native EpCAM on the cell surface of human HCT116 cells, the positive mAb-producing hybridoma cells were established by the limiting dilution procedure. The number of established clones is indicated in the last row.

To assess the difference in mAb productivity between TC-mAb and WT mice, we can focus on the proportion rather than the absolute number of antigen-specific mAb-producing B cells. Because a sufficient number of hybridoma cells ($2–8\times10^{4}$ cells) can be obtained from a fraction of the lymphocytes in spleen (1×10^{8} cells) by electrofusion, the efficiency of hybridoma production depends on the proportion of antigen-specific B cells in the spleen.

Supplementary Table 7| K_D values of obtained mAbs.				
**Target protein**	**Clone name**	**ka (1/Ms)**	**kd (1/s)**	**KD (M)**
**EpCAM**	hZAK1C008	7.60×10^4	4.88×10^{-3}	6.42×10^{-8}
	hZAK1C015	1.92×10^4	5.18×10^{-4}	2.70×10^{-8}
	hZAK2C018	12.70×10^4	11.29×10^{-3}	8.85×10^{-8}
	hZAK3C014	2.51×10^4	5.13×10^{-4}	2.05×10^{-8}
	hZAK3C101	8.10×10^4	13.93×10^{-3}	1.71×10^{-8}
**AMIGO2**	hTNK1C006	5.77×10^4	5.11×10^{-4}	8.86×10^{-9}
	hTNK1C017	2.93×10^4	2.67×10^{-4}	9.11×10^{-9}
	hTNK1C032	3.57×10^4	2.45×10^{-4}	6.86×10^{-9}
	hTNK1C041	2.20×10^4	2.92×10^{-4}	1.33×10^{-8}
	hTNK1C099	2.21×10^4	3.43×10^{-4}	1.55×10^{-8}

K_D values of mAbs isolated from TC-mAb mice immunized with recombinant protein EpCAM or AMIGO2, determined by SPR. The recombinant proteins containing the whole region of the extracellular domain were applied to the Ab captured sensor chip, respectively. And the K_D values were determined by kinetic titration procedure.

Supplementary Table 8| Antibody reagents used in experiments.					
**Antibody**	**Target species**	**fluorochrome**	**Dilution**	**Supplier**	**Clone**
**B220**	Mouse	APC	1:50	Biolegend	RA3-6B2
	Mouse	BV650	1:100	Biolegend	RA3-6B2
	Mouse	PE/Cy7	1:200	Biolegend	RA3-6B2
**CD19**	Mouse	BUV661	1:100	BD Biosciences	1D3
	Mouse	PE	1:200	Biolegend	1D3
	Mouse	PerCP/Cy5.5	1:200	Biolegend	1D3
	Isotype control	PerCP/Cy5.5	1:200	Biolegend	RTK2758
**IgM**	Human	PE/CF594	1:100	BD Biosciences	G20-127
	Mouse	PE/CF594	1:100	BD Biosciences	R6-60.2
**IgD**	Human	PE/Cy7	1:100	Biolegend	IA6-2
	Mouse	PE/Cy7	1:100	Biolegend	11-26c.2a
**IgG**	Human	PE/Cy7	1:100	Biolegend	M1310G05
	Mouse	BV421	1:200	Jackson ImmunoResearch	Goat poly 115-675-071
**Ig kappa**	Human	biotin	1:500	Biolegend	MHK-49
	Isotype control	biotin	1:500	Biolegend	MOPC-21
	Mouse	biotin	1:500	Biolegend	RMK-12
	Isotype control	biotin	1:500	BD Biosciences	R3-34
**Ig lambda**	Mouse	BV650	1:200	BD Biosciences	R26-46
	Isotype control	BV650	1:200	BD Biosciences	R35-95
**GL7**	Mouse	Alexa Fluor 647	1:100	Biolegend	GL7
	Mouse	PE	1:200	Biolegend	GL7
	Mouse	Pacific Blue	1:500	Biolegend	GL7
**CD35**	Mouse	biotin	1:100	BD Biosciences	8C12
**CD38**	Mouse	BV650	1:100	BD Biosciences	90/CD38
	Mouse	PE/Dazzle594	1:200	Biolegend	90/CD38
**CXCR4**	Mouse	PE/Dazzle594	1:200	Biolegend	L276F12
**CD86**	Mouse	PE/Cy7	1:200	Biolegend	GL-1
**CD93**	Mouse	BUV661	1:200	BD Biosciences	AA4.1
	Isotype control	BUV661	1:200	BD Biosciences	R35-38
**CD21**	Mouse	PE/Cy7	1:100	Biolegend	7E9
	Mouse	BV421	1:200	Biolegend	7E9
**CD23**	Mouse	BV650	1:200	BD Biosciences	B3B4
**CD43**	Mouse	PE/Dazzle594	1:200	Biolegend	S11
**CD5**	Mouse	PE/Cy5	1:200	Biolegend	53-7.3
**CD138**	Mouse	PE/Cy7	1:100	Biolegend	281-2
**TACI**	Mouse	PE	1:100	Biolegend	8F10

Supplementary Table 9| B cell development (Unimmunized state).			
**Mice**	**Gating conditions**	**ICR (n=5) (±SD)**	**TC-mAb mice (n=5) (±SD)**
**Bone marrow**			
Total cells (×10^6)		38.1 (±12.7)	20.0 (±0.8)
Lymphocytes (×10^6)	FSC, SSC	5.8 (±1.3)	1.5 (±0.7)
**% Lymphocyte**			
Pro-B and Pre-B	$B220^+IgM^-$	42.5 (±7.3)	39.8 (±7.1)
Immature B	$B220^+IgM^+$	27.8 (±2.8)	14.1 (±1.7)
Recirculating B	$B220^{hi}IgM^+$	11.7 (±3.7)	11.1 (±6.0)

Data represent the average ± SD of five independent experiments from different animals.

Supplementary Table 10| Data collection of B cell development (IgM/IgD).

Mice	Gating conditions	ICR		TC-mAb mice	
		Unimmunized (n=5)		Unimmunized (n=5(Sp),4(LN))	
		Absolute number	%Subset	Absolute number	%Subset
**Spleen**		**(×10^6)**	**%Lymphocyte**	**(×10^6)**	**%Lymphocyte**
Total cells		99.6 (±23.1)		37.6 (±11.2)	
Lymphocytes	FSC, SSC	41.3 (±4.9)		21.6 (±4.8)	
Igκ$^+$	CD19$^+$Igκ$^+$	12.8 (±2.7)	31.0 (±4.8)	3.4 (±1.3)	16.1 (±5.7)
Immature B	CD19$^+$CD93$^+$	0.6 (±0.5)	1.4 (±1.1)	0.1 (±0.1)	0.5 (±0.4)
Igκ$^+$ Immature B	CD19$^+$CD93$^+$Igκ$^+$	0.6 (±0.5)	1.4 (±1.0)	0.1 (±0.1)	0.4 (±0.3)
			**% Igκ$^+$ Immature B**		**% Igκ$^+$ Immature B**
T1	CD19$^+$CD93$^+$Igκ$^+$IgM^{hi}CD23$^-$	0.2 (±0.2)	35.5 (±8.9)	0.01 (±0.01)	18.5 (±2.8)
T2	CD19$^+$CD93$^+$Igκ$^+$IgM^{hi}CD23$^+$	0.1 (±0.1)	14.9 (±4.2)	0.03 (±0.02)	49.1 (±6.7)
T3	CD19$^+$CD93$^+$Igκ$^+$IgM^{lo}CD23$^+$	0.1 (±0.1)	15.0 (±5.1)	0.004 (±0.005)	3.9 (±2.2)
			**% Mature B**		**% Mature B**
Mature B	CD19$^+$CD93$^-$	12.5 (±2.6)		3.6 (±1.3)	
Follicular B	CD19$^+$CD93$^-$CD21^{lo}CD23^{hi}	8.7 (±1.0)	71.2(±7.4)	2.2 (±0.6)	62.6 (±6.2)
Marginal zone B	CD19$^+$CD93$^-$CD21^{hi}CD23^{lo}	1.8 (±0.5)	13.9 (±2.3)	0.6 (±0.5)	15.8 (±5.8)
			**% Igκ$^+$ Follicular B**		**% Igκ$^+$ Follicular B**
Igκ$^+$ Follicular B	CD19$^+$CD93$^-$CD21^{lo}CD23^{hi}Igκ$^+$	8.6 (±1.0)		2.1 (±0.6)	
IgM$^+$IgD$^-$	CD19$^+$CD93$^-$CD21^{lo}CD23^{hi}Igκ$^+$IgM$^+$IgD$^-$	0.002 (±0.001)	0.03 (±0.01)	0.4 (±0.1)	18.1 (±3.2)
IgM$^+$IgD$^+$	CD19$^+$CD93$^-$CD21^{lo}CD23^{hi}Igκ$^+$IgM$^+$IgD$^+$	8.5 (±1.0)	99.0 (±0.5)	1.7 (±0.4)	81.8 (±3.2)
IgM$^-$IgD$^+$	CD19$^+$CD93$^-$CD21^{lo}CD23^{hi}Igκ$^+$IgM$^-$IgD$^+$	0.09 (±0.04)	1.0 (±0.5)	0.0001 (±0.0001)	0.004 (±0.005)
**Lymph node**		**(×10^5)**	**%Lymphocyte**	**(×10^5)**	**%Lymphocyte**
Total cells		40.6 (±1.8)		53.3 (±18.0)	
Lymphocytes	FSC, SSC	28.4 (±12.2)		39.9 (±14.4)	
Igκ$^+$	CD19$^+$Igκ$^+$	0.2 (±0.1)	6.5 (±1.3)	0.2 (±0.1)	5.3 (±1.8)
			**% Mature B**		**% Mature B**
Mature B	CD19$^+$CD93$^-$	1.9 (±1.0)		2.3 (±1.4)	
Follicular B	CD19$^+$CD93$^-$CD21^{lo}CD23^{hi}	1.8 (±1.0)	95.4 (±1.2)	2.2 (±1.3)	94.0 (±2.0)
			**% Igκ$^+$ Follicular B**		**% Igκ$^+$ Follicular B**
Igκ$^+$ Follicular B	CD19$^+$CD93$^-$CD21^{lo}CD23^{hi}Igκ$^+$	1.8 (±0.9)		2.0 (±1.2)	
IgM$^+$IgD$^-$	CD19$^+$CD93$^-$CD21^{lo}CD23^{hi}Igκ$^+$IgM$^+$IgD$^-$	0.0005 (±0.0003)	0.03 (±0.03)	0.3 (±0.2)	13.6 (±2.9)
IgM$^+$IgD$^+$	CD19$^+$CD93$^-$CD21^{lo}CD23^{hi}Igκ$^+$IgM$^+$IgD$^+$	1.7 (±0.8)	95.5 (±1.8)	1.7 (±1.0)	86.4 (±3.0)
IgM$^-$IgD$^+$	CD19$^+$CD93$^-$CD21^{lo}CD23^{hi}Igκ$^+$IgM$^-$IgD$^+$	0.1 (±0.1)	4.5 (±1.7)	0.0003 (±0.0002)	0.02 (±0.01)

Data represent the average ± SD of five or four independent experiments from different animals.

Supplementary Table 11| Data collection of B cell development (B1a/b. B2).

		ICR mice						TC-mAb mice					
**Mice**	**Gating conditions**	**6w (n=3)**		**8w (n=3)**		**20w (n=3)**		**6w (n=3)**		**8w (n=3)**		**20w (n=3)**	
		**Absolute number**	**%Subset**	**Absolute number**	**%Subset**	**Absolute number**	**%Subset**	**Absolute number**	**%Subset**	**Absolute number**	**%Subset**	**Absolute number**	**%Subset**
**Spleen**		**(×10^6)**		**(×10^6)**		**(×10^6)**		**(×10^6)**		**(×10^6)**		**(×10^6)**	
Total cells		145.9 (±34.2)		114.9 (±12.9)		200.8 (±78.3)		38.1 (±6.8)		41.3 (±13.5)		45.9 (±17.7)	
Lymphocytes	FSC, SSC	78.9 (±12.8)		60.8 (±5.7)		113.0 (±32.3)		18.3 (±4.2)		25.4 (±6.6)		26.3 (9.6)	
**B2**			**%Lymphocyte**		**%Lymphocyte**		**%Lymphocyte**		**%Lymphocyte**		**%Lymphocyte**		**%Lymphocyte**
Mature B	$B220^{hi}CD19^{+}CD93^{-}$	27.5 (±2.4)	35.6 (±4.9)	26.1 (±1.5)	43.1 (±1.7)	53.3 (±13.5)	47.7 (±2.0)	3.1 (±0.6)	17.0 (±0.5)	4.2 (±1.0)	17.8 (±5.7)	7.0 (±2.0)	26.9 (±1.2)
			**%Mature B**		**%Mature B**		**%Mature B**		**%Mature B**		**%Mature B**		**%Mature B**
Follicular B	$B220^{hi}CD19^{+}CD93^{-}CD21^{lo}CD23^{hi}$	21.2 (±1.5)	77.1 (±1.9)	20.2 (±0.6)	77.7 (±4.0)	40.3 (±12.9)	74.0 (±6.4)	2.3 (±0.5)	74.1 (±1.4)	3.1 (±0.5)	74.7 (±6.4)	5.3 (±1.5)	76.4 (±1.6)
Marginal zone B	$B220^{hi}CD19^{+}CD93^{-}CD21^{hi}CD23^{lo}$	2.4 (±0.2)	8.8 (±0.9)	3.1 (±0.3)	11.7 (±0.3)	6.6 (±1.4)	14.4 (±7.5)	0.3 (±0.1)	10.6 (±1.2)	0.6 (±0.2)	12.7 (±2.4)	0.9 (±0.2)	13.2 (±0.8)
MZ/FOL ratio		0.11 (±0.01)		0.15 (±0.01)		0.20 (±0.12)		0.14 (±0.02)		0.17 (±0.04)		0.17 (±0.01)	
**B1a**			**%Lymphocyte**		**%Lymphocyte**		**%Lymphocyte**		**%Lymphocyte**		**%Lymphocyte**		**%Lymphocyte**
$CD19^{+}B220^{lo}$	$CD19^{+}B220^{lo}$	1.2 (±0.2)	1.7 (±0.2)	1.3 (±0.8)	2.4 (±1.5)	1.9 (±1.1)	1.8 (±0.9)	1.6 (±0.5)	8.1 (±0.6)	1.3 (±0.3)	5.2 (±0.2)	1.0 (±0.3)	3.8 (±0.2)
B1a	$CD19^{+}B220^{lo}CD43^{+}CD5^{+}$	1.1 (±0.2)	1.6 (±0.2)	1.2 (±0.8)	2.2 (±1.4)	1.6 (±1.1)	1.5 (±0.8)	1.5 (±0.5)	7.6 (±0.5)	1.2 (±0.3)	4.8 (±0.2)	0.8 (±0.3)	3.1 (±0.6)
**PECs**		**(×10^5)**		**(×10^5)**		**(×10^5)**		**(×10^5)**		**(×10^5)**		**(×10^5)**	
Total cells		102. 0(±33.8)		126.5 (±81.0)		120.4 (±14.4)		32.8 (±7.7)		39.8 (±21.3)		85.5 (±19.0)	
Live cells	FSC, SSC, $DAPI^{-}$	51. 5(±16.7)		62.5 (±35.9)		63.7 (±9.4)		20.5 (±3.6)		19.6 (±10.4)		42.6 (±12.4)	
			**% Live cells**		**% Live cells**		**% Live cells**		**% Live cells**		**% Live cells**		**% Live cells**
$CD19^{+}$	$CD19^{+}$	3.2 (±2.0)	6.1 (±4.4)	14.6 (±11.3)	21.5 (±9.0)	18.9 (±14.6)	30.6 (±24.5)	2.8 (±0.7)	13.6 (±1.6)	5.9 (±1.8)	33.9 (±6.9)	14.1 (±8.2)	33.1 (±18.3)
B1a	$CD19^{+}CD23^{-}CD5^{+}$	1.3 (±0.9)	2.3 (±1.6)	5.2 (±2.5)	10.5 (±7.6)	9.5 (±8.4)	15.6 (±14.0)	2.2 (±0.6)	10.5 (±1.2)	4.2 (±1.3)	24.2 (±4.8)	9.3 (±5.8)	21.7 (±12.4)
B1b	$CD19^{+}CD23^{-}CD5^{-}$	0.3 (±0.1)	0.6 (±0.2)	1.6 (±1.6)	1.9 (±1.1)	2.4 (±1.4)	3.8 (±2.4)	0.2 (±0.1)	1.2 (±0.3)	0.8 (±0.3)	4.3 (±1.0)	2.6 (±1.5)	5.9 (±2.9)
B2	$CD19^{+}CD23^{+}CD5^{-}$	1.5 (±1.1)	3.0 (±2.6)	7.6 (±8.4)	8.8 (±6.1)	6.9 (±4.8)	11.1 (±8.0)	0.3 (±0.1)	1.6 (±0.3)	0.8 (±0.2)	4.9 (±2.0)	2.1 (±1.1)	5.3 (±3.2)

Data represent the average ± SD of three independent experiments from different animals.

Supplementary Table 12| Data collection of B cell development (Germinal centre B).

Mice	Gating conditions			OVA-immunized							
		Unimmunized (n=3)		Day7 (n=3)		Day14 (n=3)		Day21 (n=3)		Day28 (n=3)	
		Absolute number ($\times 10^6$)	%Subset	Absolute number ($\times 10^6$)	%Subset	Absolute number ($\times 10^6$)	%Subset	Absolute number ($\times 10^6$)	%Subset	Absolute number ($\times 10^6$)	%Subset
**ICR Spleen**											
Total cells		174.3 (±37.8)		298.7 (±81.0)		310.6 (±127.6)		155.4 (±69.2)		205.7 (±74.8)	
Lymphocytes	FSC, SSC	97.7 (±22.3)		71.5 (±33.1)		73.7 (±17.4)		65.6 (±25.0)		79.3 (±21.9)	
			**%Lymphocyte**		**%Lymphocyte**		**%Lymphocyte**		**%Lymphocyte**		**%Lymphocyte**
$CD19^+$	$CD19^+$	57.3 (±12.7)	58.8 (±3.5)	40.4 (±19.3)	56.1 (±5.0)	43.2 (±12.4)	58.4 (±8.5)	40.7 (±17.8)	60.7 (±7.2)	44.0 (±9.6)	56.8 (±5.3)
Germinal centre B	$CD19^+CD38^{lo/-}GL7^+$	0.2 (±0.3)	0.3 (±0.2)	0.1 (±0.1)	0.2 (±0.1)	1.3 (±1.2)	1.8 (±1.4)	0.3 (±0.3)	0.6 (±0.6)	0.3 (±0.3)	0.5 (±0.5)
			**%GC**		**%GC**		**%GC**		**%GC**		**%GC**
Dark zone B	$CD19^+CD38^{lo/-}GL7^+CXCR4^{hi}CD86^{lo}$	0.2 (±0.2)	53.9 (±11.6)	0.1 (±0.1)	63.4 (±3.0)	0.7 (±0.7)	55.7 (±7.3)	0.1 (±0.1)	47.3 (±8.2)	0.1 (±0.1)	42.0 (±9.3)
Light zone B	$CD19^+CD38^{lo/-}GL7^+CXCR4^{lo}CD86^{hi}$	0.1 (±0.1)	33.9 (±4.4)	0.04 (±0.03)	29.8 (±2.4)	0.5 (±0.4)	34.0 (±6.5)	0.2 (±0.2)	41.1 (±10.4)	0.1 (±0.1)	30.0 (±14.6)
**TC-mAb Spleen**											
Total cells		44.0 (±10.0)		100.0 (±31.2)		103.7 (±43.2)		147.3 (±50.3)		67.5 (±7.1)	
Lymphocytes	FSC, SSC	21.7 (±4.9)		16.9 (±3.1)		23.0 (±2.3)		35.3 (±8.3)		25.5 (±3.9)	
			**%Lymphocyte**		**%Lymphocyte**		**%Lymphocyte**		**%Lymphocyte**		**%Lymphocyte**
$CD19^+$	$CD19^+$	7.2 (±2.1)	32.3 (±3.4)	5.6 (±1.0)	33.2 (±2.5)	8.6 (±1.1)	37.6 (±4.0)	11.3 (±2.8)	32.1 (±1.1)	9.1 (±1.9)	35.4 (±2.0)
Germinal centre B	$CD19^+CD38^{lo/-}GL7^+$	0.18 (±0.01)	0.24 (±0.01)	0.1 (±0.1)	0.4 (±0.1)	0.3 (±0.2)	1.2 (±1.0)	0.81 (±0.02)	2.8 (±0.1)	0.7 (±0.1)	1.6 (±0.3)
			**%GC**		**%GC**		**%GC**		**%GC**		**%GC**
Dark zone B	$CD19^+CD38^{lo/-}GL7^+CXCR4^{hi}CD86^{lo}$	0.12 (±0.03)	67.0 (±12.0)	0.07 (±0.04)	68.7 (±6.0)	0.2 (±0.1)	61.4 (±3.7)	0.50 (±0.04)	62.4 (±4.0)	0.4 (±0.1)	51.2 (±6.9)
Light zone B	$CD19^+CD38^{lo/-}GL7^+CXCR4^{lo}CD86^{hi}$	0.03 (±0.02)	14.9 (±5.8)	0.02 (±0.01)	18.5 (±1.6)	0.1 (±0.1)	24.9 (±7.7)	0.24 (±0.02)	29.2 (±2.5)	0.3 (±0.1)	41.1 (±9.9)

Data represent the average ± SD of three independent experiments from different animals.

Supplementary Table 13| Data collection of B cell development (Allelic exclusion).					
		ICR Unimmunized (n=3)		TC-mAb mice Unimmunized (n=3)	
**Mice**	**Gating conditions**	**Absolute number ($\times 10^6$)**	**%Subset**	**Absolute number ($\times 10^6$)**	**%Subset**
**Spleen**					
Total cells		141.5 ($\pm$46.5)		33.5 ($\pm$11)	
Lymphocytes	FSC, SSC	78.2 ($\pm$20.6)		18.1 ($\pm$4.6)	
			**%Lymphocyte**		**%Lymphocyte**
Immature B	$CD19^+CD93^+$	2.9 ($\pm$1.3)	3.4 ($\pm$1.0)	0.1 ($\pm$0.1)	0.8 ($\pm$0.1)
Mature B	$CD19^+CD93^-$	37.9 ($\pm$9.4)	49.2 ($\pm$6.1)	5.7 ($\pm$1.8)	31.2 ($\pm$2.9)
			**%IgM$^+$ Mature B**		**%IgM$^+$ Mature B**
IgM$^+$ mature B	$CD19^+CD93^-IgM^+$	37.3 ($\pm$9.2)		5.7 ($\pm$1.7)	
$Ig\kappa^+Ig\lambda^-$	$CD19^+CD93^-IgM^+Ig\kappa^+Ig\lambda^-$	33.7 ($\pm$8.3)	90.4 ($\pm$3.4)	4.5 ($\pm$1.2)	79.9 ($\pm$3.9)
$Ig\kappa^-Ig\lambda^+$	$CD19^+CD93^-IgM^+Ig\kappa^-Ig\lambda^+$	0.7 ($\pm$0.3)	1.9 ($\pm$0.5)	0.6 ($\pm$0.4)	10.1 ($\pm$4.0)
$Ig\kappa^+Ig\lambda^+$	$CD19^+CD93^-IgM^+Ig\kappa^+Ig\lambda^+$	0.0 ($\pm$0.0)	0.0 ($\pm$0.0)	0.0 ($\pm$0.0)	0.0 ($\pm$0.0)
			**%IgM$^+$ Immature B**		**%IgM$^+$ Immature B**
IgM$^+$ Immature B	$CD19^+CD93^+IgM^+$	2.8 ($\pm$1.3)		0.14 ($\pm$0.04)	
$Ig\kappa^+Ig\lambda^-$	$CD19^+CD93^+IgM^+Ig\kappa^+Ig\lambda^-$	1.9 ($\pm$1.0)	62.2 ($\pm$14.3)	0.05 ($\pm$0.01)	36.8 ($\pm$11.2)
$Ig\kappa^-Ig\lambda^+$	$CD19^+CD93^+IgM^+Ig\kappa^-Ig\lambda^+$	0.2 ($\pm$0.1)	6.7 ($\pm$2.7)	0.07 ($\pm$0.04)	47.4 ($\pm$13.5)
$Ig\kappa^+Ig\lambda^+$	$CD19^+CD93^+IgM^+Ig\kappa^+Ig\lambda^+$	0.04 ($\pm$0.02)	1.5 ($\pm$0.2)	0.004 ($\pm$0.0001)	2.7 ($\pm$1.1)

Data represent the average $\pm$ SD of three independent experiments from different animals.

Supplementary Table 14| Data collection of B cell development (Comparison between unimmunized and immunized state).

		ICR				TC-mAb mice			
		Unimmunized (n=5)		Immunized (n=5)		Unimmunized (n=5)		Immunized (n=5)	
**Mice**	**Gating conditions**	**Absolute number**	**%Subset**	**Absolute number**	**%Subset**	**Absolute number**	**%Subset**	**Absolute number**	**%Subset**
		**($\times 10^6$)**		**($\times 10^6$)**		**($\times 10^6$)**		**($\times 10^6$)**	
**Spleen**									
Total cells		132.0 (± 32.3)		428.7 (± 64.8)		27.0 (± 8.3)		122.1 (± 54.1)	
Lymphocytes	FSC, SSC	84.6 (± 25.1)		231.6 (± 28.9)		17.0 (± 6.6)		54.4 (± 18.6)	
			**%Lymphocyte**		**%Lymphocyte**		**%Lymphocyte**		**%Lymphocyte**
Plasmablast, Plasma cell	$CD138^{+}TACI^{+}$	0.6 (± 0.2)	0.8 (± 0.2)	5.7 (± 3.0)	2.4 (± 1.0)	0.7 (± 0.3)	3.9 (± 1.1)	3.5 (± 2.0)	6.5 (± 2.7)

Data represent the average ± SD of five independent experiments from different animals.

Supplementary Table 15| Characteristics of TC-mAb mice for antibodies production.

	Wild type mice	TC-mAb mice	Double Tc mice[11]	KM mouse[12]	Tc cattle[28]	XenoMouse[18]
**Carrier of human Ig locus**	—	IGHK-NAC	HCF14 and HCF2	HCF14 and *IGK* gene mini-locus	istHAC	Non-targeting insertion
**human Ig-genes**	—	Full length of *IGH* and *IGK*	Full length of *IGH* and *IGK*	Full length of *IGH* and partial *IGK*	Full length of *IGH*, *IGK,* and *IGL*	Partial length of *IGH* and *IGK*
**Stability of Ig-genes**	Stable (endogenous)	Stable	Instable	Instable (*IGH*)	Stable	Stable
**IgG concentration in anti-sera**	Normal	Decrease	Decrease	—	Normal	Decrease
**Immune response**	Normal	Delayed	—	—	—	—
**Hybridoma production frequency**	Normal	High	One-tenth of normal	Improved against Double Tc mice	—	—
**Antibody titre**	Normal	Normal	Normal or weak	—	Normal	Normal or weak
**IgG subclass (unimmunized)**	Normal	IgG1≈2>3,4	IgG1>2>3,4	—	—	—
**IgG subclass (immunized with protein)**	Normal	IgG1>3>2>4	—	—	—	—
**Human Ig repertoires**	—	Closely similar to hPBMCs	—	—	—	Some differences
**Spleen size and number of lymphocytes**	Normal	Decrease	—	—	—	—
**B cell development**	Normal	Adverse	Adverse	—	—	Adverse

-: There is no known indication.

Supplementary Table 16| Primer list.

	Gene or aim	Primer name (forward)	Forward primer (5′-3′)	Primer name (reverse)	Reverse primer (5′-3′)	Product size
**Genomic PCR**						
	hChr.2: arm for loxP insertion	cos138-F6B	TCGAGGATCCCACATAGACATTCAACCGCAAAGCAG	cos138-R6B	TCGAGGATCCAGGCCCTACACATCAAAAAGTGAAGCAG	9.5 kb
	hChr.2: loxP targeting check	cos138 sp L	CTGAGAAGAGTCATTGTTTATGGTAGACT	cos138 sp R	ATCCCCATGTGTATCACTGGCAAACTGT	4,143 bp
	hChr.2: loxP targeting check	x6.1cosRa L	GGGGAATAAACACCCTTTCCAAATCCTC	x6.1cosRa R	ACCAAGTAACCGATCAAACCAACCCTTG	9,117 bp
	D2S177	D2S177 F	AGCTCAGAGACACCTCTCCA	D2S177 R	CTGTATTAGGATACTTGGCTATTGA	292 bp
	FABP1	FABP1-F	TATCAAGGGGGTGTCGGAAATCGTG	FABP1-R	ACTGGGCCTGGGAGAACCTGAGACT	444 bp
	EIF2AK3	EIF2AK3-F	AGGTGCTGCTGGGTGGTCAAGT	EIF2AK3-R	GCTCCTGCAAATGTCTCCTGTCA	695 bp
	RPIA	RPIA-F	CTTACCCAGGCTCCAGGCTCTATT	RPIA-R	CTCTACCTCCCTACCCCATCATCAC	631 bp
	IGKC	IGKC-F	TGGAAGGTGGATAACGCCCT	IGKC-R	TCATTCTCCTCCAACATTAGCA	377 bp
	IGKV	IGKV-F	AGTCAGGGCATTAGCAGTGC	IGKV-R	GCTGCTGATGGTGAGAGTGA	156 bp
	IGKV	Vk3-2 F	CTCTCCTGCAGGGCCAGTCA	Vk3-2 R	TGCTGATGGTGAGAGTGAACTC	169 bp
	D2S159_1	D2S159_1 F	CTCTAACTGAATCAAGGGAATGAAC	D2S159_1 R	AGCAGTTTGAGTTTAGGATGAAGG	201 bp
	hChr.2: arm for FRT insertion	kD-R9La L	TCGAGCGGCCGCAGGATCTTTGGGGGACTGAATGGGGTGTGCT	kD-R9La R	TCGAACGCGTTGGAACCCTCATACGTTGCTGGTGGAATGT	4.0 kb
	hChr.2: arm for FRT insertion	KD-F9Ra L	CGAGGATCCATTTCTCCACATCCTAGCCAACACTTGACATTTCCT	KD-F9Ra R	TCGAGGATCCGCCAGGGAGACAGATGCCAAGTACGGTTTAG	3.2 kb
	hChr.2: FRT targeting check	kD9 tcLa L	TGAGAACACAGGGGTCTCCATTCTGACT	kD9 tcLa R	ACAATCAACAGCATCCCCATCTCTGAAG	4,951 bp
	hChr.2: FRT targeting check	kD9 tcRa L	GACGTGCTACTTCCATTTGTCACGTCCT	kD9 tcRa R	TGGTCACTGAAGCTTTCCATCTGCTCTT	3,538 bp
	Cre-loxP recombination	TRANSL1	TGGAGGCCATAAACAAGAAGAC	TRANSR1	CCCCTTGACCCAGAAATTCCA	409 bp
	Cre-loxP recombination	kj neo	CATCGCCTTCTATCGCCTTCTTGACG	PGKr-2	ATCTGCACGAGACTAGTGAGACGTGCTA	~600 bp
	hChr.14: arm for FRT insertion	NotISC355-F	TCGAGCGGCCGCGTACAATCTTGGATCACTACAACCTCTGCCTA	AscISC355-R	TCGAGGCGCGCCAGGATTATAGATGTGAGCCATCACTAAGACTCCT	3.8 kb
	hChr.14: arm for FRT insertion	SalISC355-F4	TCGAGTCGACAGCACGTTGGGAGGCCAAGGCAGGAGAATA	BamHISC355-R4	TCGAGGATCCTGGCTGACACAGCCAGTCCCGGATT	4.2 kb
	hChr.14: FRT targeting check	14TarC_La F	AGCAATTAGGGCCTGTGCATCTCACTTT	14TarC_La R	CCAGCTCATTCCTCCCACTCATGATCTA	4,151 bp
	hChr.14: FRT targeting check	14TarC_Ra F	CATCTGGAGTCCTATTGACATCGCCAGT	14TarC_Ra R	CTTATTCCTCCTTCTGCCCACCCTTCAT	5,026 bp
	MTA1	MTA1-F3	AGCACTTTACGCATCCCAGCATGT	MTA1-R3	CCAAGAGAGTAGTCGTGCCCCTCA	486 bp
	ELK2P2	ELK2P2-F	CCCACTTTACCGTGCTCATT	ELK2P2-R	ATGAAGGTCCGTGACTTTGG	540 bp
	IGHG1	g1(g2)-F	ACCCCAAAGGCCAAACTCTCCACTC	g1(g2)-R	CACTTGTACTCCTTGCCATTCAGC	520 bp
	IGHV3-74	VH3-F	AGTGAGATAAGCAGTGGATG	VH3-R	CTTGTGCTACTCCCATCACT	247 bp
	IGHM	CH3F3	AGGCCAGCATCTGCGAGGAT	CH4R2	GTGGCAGCAAGTAGACATCG	326 bp
	Flp-FRT recombination	TRANSL1	TGGAGGCCATAAACAAGAAGAC	TRANSR1	CCCCTTGACCCAGAAATTCCA	409 bp
	Flp-FRT recombination	PGKr-2	ATCTGCACGAGACTAGTGAGACGTGCTA	CMVr-1	CCTATTGGCGTTACTATGGGAACATACG	444 bp
**RT-PCR**						
	IgK	Vk1BACK	GACATCCAGCTGACCCAGTCTCC	Ck	CAGAGGCAGTTCCAGATTTC	
	IgM	VH4BACK	CAGGTGCAGCTGCAGGAGTCGGG	Cmu-1	CAGGAGAAAGTGATGGAGTC	
	GAPDH	RPC1	CCATCTTCCAGGAGCGAGA	RPC2	TGTCATACCAGGAAATGAGC	722 bp
**Deep sequencing**						
	cDNA synthesis	BSL-18E	AAAGCGGCCGCATGCTTTTTTTTTTTT TTTTTTVN			
	Adaptor	P10EA	GGGAATTCGG			
	Adaptor	P20EA	TAATACGACTCCGAATTCCC			
	IgG constant region	CG1	CACCTTGGTGTTGCTGGGCTT			
	Second PCR	CB2	AGGCAGTATCTGGAGTCATTGAG			
	Second PCR	CG2	TCCTGAGGACTGTAGGACAGC			
	Second PCR	P22EA-ST1	GTCTCGTGGGCTCGGAGATGTGTATAAGAGACAGCTAATACGACTCCGAATTCCC	CB-ST1-R	TCGTCGGCAGCGTCAGATGTGTATAAGAGACAGGCTCAAACACAGCGACCTC	
	Second PCR	P22EA-ST1	GTCTCGTGGGCTCGGAGATGTGTATAAGAGACAGCTAATACGACTCCGAATTCCC	CG-ST1-R	TCGTCGGCAGCGTCAGATGTGTATAAGAGACAGTGAGTTCCACGACACCGTCAC	
**Antigen cloning and expression**						
	ExEX	Forward	AAAGATATCGGATCCTCAGGAAGAATGTGTCTGTGA	Reverse	ATAAAGCTTTTTTAGACCCTGCATTGAGAATTC	751 bp
	AMIGO2	Forward	GCGAAGCTTGTGTGCCCCACCGCTTGCAT	Reverse	GCGCTCGAGTGTGTTAAATGCCTCATGAGCATGGG	1095 bp
	Inserted fragment in pGEX6P1		ACGAGATCTGCCATGGACAAGCTTGTCGACACGAGCTCGAATTCGGATCCCCCGGGGCTCGAGCACCACCACCACCACCACTGAGCTGAGCGGCCGCTCA			100 bp

Supplementary Table 17| Antibodies for determining serum immunoglobulin concentrations.						
		**Antibody**	**Labelling**	**Dilution**	**Supplier**	**Clone**
**hIg μ**	Capture	Goat anti-Human IgM	-	1:100	Bethyl Laboratories	Goat polyclonal A80-100A-11
	Detector	Goat anti-Human IgM	HRP	1:75000	Bethyl Laboratories	Goat polyclonal A80-100P-37
**hIg γ**	Capture	Goat anti-human IgG-Fc	-	1:100	Bethyl Laboratories	Goat polyclonal A80-104A-9
	Detector	Goat anti-human IgG-Fc	HRP	1:150000	Bethyl Laboratories	Goat polyclonal A80-104P-90
**hIg κ**	Capture	Goat anti-Human Ig kappa	-	1:300	Bethyl Laboratories	Goat polyclonal A80-115A-6
	Detector	Goat anti-Human Ig kappa	HRP	1:150000	Bethyl Laboratories	Goat polyclonal A80-115P-43
**hIg α**	Capture	Goat anti-Human IgA	-	1:100	Bethyl Laboratories	Goat polyclonal A80-102A-6
	Detector	Goat anti-Human IgA	HRP	1:75000	Bethyl Laboratories	Goat polyclonal A80-102P-26
**hIg ε**	Capture	Goat anti-Human IgE	-	1:100	Bethyl Laboratories	Goat polyclonal A80-108A-15
	Detector	Goat anti-Human IgE	HRP	1:75000	Bethyl Laboratories	Goat polyclonal A80-108P-35
**mIg μ**	Capture	Goat anti-Mouse IgM	-	1:100	Bethyl Laboratories	Goat polyclonal A90-101A-22
	Detector	Goat anti-Mouse IgM	HRP	1:75000	Bethyl Laboratories	Goat polyclonal A90-101P-34
**mIg γ**	Capture	Goat anti-Mouse IgG-Fc	-	1:100	Bethyl Laboratories	Goat polyclonal A90-131A-16
	Detector	Goat anti-Mouse IgG-Fc	HRP	1:100000	Bethyl Laboratories	Goat polyclonal A90-131P-38
**mIg κ**	Capture	Goat anti-Mouse Ig kappa	-	1:100	Bethyl Laboratories	Goat polyclonal A90-119A-14
	Detector	Goat anti-Mouse Ig kappa	HRP	1:100000	Bethyl Laboratories	Goat polyclonal A90-119P
**mIg λ**	Capture	Goat anti-Mouse Ig lambda	-	1:100	Bethyl Laboratories	Goat polyclonal A90-121A-12
	Detector	Goat anti-Mouse Ig lambda	HRP	1:100000	Bethyl Laboratories	Goat polyclonal A90-121P-18
