# Supplementary material for: Efficient human-like antibody repertoire and hybridoma production in trans-chromosomic mice carrying megabase-sized human immunoglobulin loci
Source: Nat Commun. 2022 Apr 5;13:1841. doi: 10.1038/s41467-022-29421-2 (PMC8983744; doi:10.1038/s41467-022-29421-2)
Supplement: Supplementary file 3 — Reporting Summary [file 41467_2022_29421_MOESM3_ESM.pdf]

nature research

Corresponding author(s): Yasuhiro Kazuki

Last updated by author(s): Feb 24, 2022

Reporting Summary

Nature Research wishes to improve the reproducibility of the work that we publish. This form provides structure for consistency and transparency in reporting. For further information on Nature Research policies, see our Editorial Policies and the Editorial Policy Checklist.

Statistics

For all statistical analyses, confirm that the following items are present in the figure legend, table legend, main text, or Methods section.

n/a	Confirmed	
☐	☒	The exact sample size (*n*) for each experimental group/condition, given as a discrete number and unit of measurement
☐	☒	A statement on whether measurements were taken from distinct samples or whether the same sample was measured repeatedly
☐	☒	The statistical test(s) used AND whether they are one- or two-sided<br>*Only common tests should be described solely by name; describe more complex techniques in the Methods section.*
☐	☒	A description of all covariates tested
☐	☒	A description of any assumptions or corrections, such as tests of normality and adjustment for multiple comparisons
☐	☒	A full description of the statistical parameters including central tendency (e.g. means) or other basic estimates (e.g. regression coefficient) AND variation (e.g. standard deviation) or associated estimates of uncertainty (e.g. confidence intervals)
☐	☒	For null hypothesis testing, the test statistic (e.g. *F*, *t*, *r*) with confidence intervals, effect sizes, degrees of freedom and *P* value noted<br>*Give P values as exact values whenever suitable.*
☒	☐	For Bayesian analysis, information on the choice of priors and Markov chain Monte Carlo settings
☒	☐	For hierarchical and complex designs, identification of the appropriate level for tests and full reporting of outcomes
☐	☒	Estimates of effect sizes (e.g. Cohen's *d*, Pearson's *r*), indicating how they were calculated

Our web collection on statistics for biologists contains articles on many of the points above.

Software and code

Policy information about availability of computer code

Data collection	FISH analysis : Metafer4 slide scanning platform software V3.10.5 (Meta Systems)<br>Antibody repertoire analysis : Sequence using on the illumina Hiseq2000/Hiseq2500 Machine<br>ELISA : BioTek EPOCH2 Gen5 version 3.03<br>Surface plasmon resonance : Biacore T200 Control and Evaluation software version 3.2.1<br>Flow cytometry data : Beckman Coulter Cytexpert version 2.3<br>IHC images : Zeiss LSM700 confocal microscope
Data analysis	FISH analysis : Isis Fluorescence Imaging System software V5.4.12 (Meta Systems)<br>Repertoire analysis : R software version 3.4.2, vegan : Community Ecology Package. R package version 2.5-6, IGBLAST database version IMTG database human Ig 20190724, IGBLASTN version 1.10.0, BLASTN version 2.6.0, and ClustalW version 2.1.<br>Principal component analysis was performed using the prcomp-function in R (version 3.4.2).<br>Circos plot and polygenetic tree images : R software using circos open-source software (www.circos.ca).<br>T20 score : T20 Cutoff Human Database (http://abanalyzer.lakepharma.com)<br>Antibody concentration : ELISA data were analyzed by Biotek Gen5 version 3.03 using 4-parameter logistic curve fitting model.<br>Statistical analyses were performed using Graphpad Prism 8 or 9 Software Inc., La Jolla, CA).<br>Flow cytometry data analysis : Kaluza software version2.1.<br>IHC images : ImageJ software.

For manuscripts utilizing custom algorithms or software that are central to the research but not yet described in published literature, software must be made available to editors and reviewers. We strongly encourage code deposition in a community repository (e.g. GitHub). See the Nature Research guidelines for submitting code & software for further information.

Data

Policy information about availability of data

All manuscripts must include a data availability statement. This statement should provide the following information, where applicable:

- Accession codes, unique identifiers, or web links for publicly available datasets
- A list of figures that have associated raw data
- A description of any restrictions on data availability

All data supporting the findings of this study are available from the corresponding author on reasonable request.

Field-specific reporting

Please select the one below that is the best fit for your research. If you are not sure, read the appropriate sections before making your selection.

☒ Life sciences ☐ Behavioural & social sciences ☐ Ecological, evolutionary & environmental sciences

For a reference copy of the document with all sections, see nature.com/documents/nr-reporting-summary-flat.pdf

Life sciences study design

All studies must disclose on these points even when the disclosure is negative.

Sample size	Sample size was determined based on literature data (Tomizuka, K. et al., Proc. Natl. Acad. Sci., 2000; Lee, E. C. et al., Nat. Biotechnol., 2014; Murphy, A. J. et al., Proc. Natl. Acad. Sci., 2014; Green, L. L. et al., Nat. Genet., 1994; Longo, N. S. et al., J. Immunol., 2017).
Data exclusions	No data were excluded.
Replication	Figure legends and Source Data contain exact number of samples and animals used in this study.
Randomization	The mice were randomly grouped prior to be treated. No randomization was used in other experiments.
Blinding	The teams involved in NGS data collection and analysis were blinded. In other experiments, the investigators were not blinded to allocation during experiments and outcome assessment. Blinding was not possible as the same investigator processed the animals and analyzed the data.

Behavioural & social sciences study design

All studies must disclose on these points even when the disclosure is negative.

Study description	*Briefly describe the study type including whether data are quantitative, qualitative, or mixed-methods (e.g. qualitative cross-sectional, quantitative experimental, mixed-methods case study).*
Research sample	*State the research sample (e.g. Harvard university undergraduates, villagers in rural India) and provide relevant demographic information (e.g. age, sex) and indicate whether the sample is representative. Provide a rationale for the study sample chosen. For studies involving existing datasets, please describe the dataset and source.*
Sampling strategy	*Describe the sampling procedure (e.g. random, snowball, stratified, convenience). Describe the statistical methods that were used to predetermine sample size OR if no sample-size calculation was performed, describe how sample sizes were chosen and provide a rationale for why these sample sizes are sufficient. For qualitative data, please indicate whether data saturation was considered, and what criteria were used to decide that no further sampling was needed.*
Data collection	*Provide details about the data collection procedure, including the instruments or devices used to record the data (e.g. pen and paper, computer, eye tracker, video or audio equipment) whether anyone was present besides the participant(s) and the researcher, and whether the researcher was blind to experimental condition and/or the study hypothesis during data collection.*
Timing	*Indicate the start and stop dates of data collection. If there is a gap between collection periods, state the dates for each sample cohort.*
Data exclusions	*If no data were excluded from the analyses, state so OR if data were excluded, provide the exact number of exclusions and the rationale behind them, indicating whether exclusion criteria were pre-established.*
Non-participation	*State how many participants dropped out/declined participation and the reason(s) given OR provide response rate OR state that no participants dropped out/declined participation.*
Randomization	*If participants were not allocated into experimental groups, state so OR describe how participants were allocated to groups, and if allocation was not random, describe how covariates were controlled.*

Ecological, evolutionary & environmental sciences study design

All studies must disclose on these points even when the disclosure is negative.

Study description	*Briefly describe the study. For quantitative data include treatment factors and interactions, design structure (e.g. factorial, nested, hierarchical), nature and number of experimental units and replicates.*
Research sample	*Describe the research sample (e.g. a group of tagged Passer domesticus, all Stenocereus thurberi within Organ Pipe Cactus National Monument), and provide a rationale for the sample choice. When relevant, describe the organism taxa, source, sex, age range and any manipulations. State what population the sample is meant to represent when applicable. For studies involving existing datasets, describe the data and its source.*
Sampling strategy	*Note the sampling procedure. Describe the statistical methods that were used to predetermine sample size OR if no sample-size calculation was performed, describe how sample sizes were chosen and provide a rationale for why these sample sizes are sufficient.*
Data collection	*Describe the data collection procedure, including who recorded the data and how.*
Timing and spatial scale	*Indicate the start and stop dates of data collection, noting the frequency and periodicity of sampling and providing a rationale for these choices. If there is a gap between collection periods, state the dates for each sample cohort. Specify the spatial scale from which the data are taken*
Data exclusions	*If no data were excluded from the analyses, state so OR if data were excluded, describe the exclusions and the rationale behind them, indicating whether exclusion criteria were pre-established.*
Reproducibility	*Describe the measures taken to verify the reproducibility of experimental findings. For each experiment, note whether any attempts to repeat the experiment failed OR state that all attempts to repeat the experiment were successful.*
Randomization	*Describe how samples/organisms/participants were allocated into groups. If allocation was not random, describe how covariates were controlled. If this is not relevant to your study, explain why.*
Blinding	*Describe the extent of blinding used during data acquisition and analysis. If blinding was not possible, describe why OR explain why blinding was not relevant to your study.*

Did the study involve field work? ☐ Yes ☐ No

Field work, collection and transport

Field conditions	*Describe the study conditions for field work, providing relevant parameters (e.g. temperature, rainfall).*
Location	*State the location of the sampling or experiment, providing relevant parameters (e.g. latitude and longitude, elevation, water depth).*
Access & import/export	*Describe the efforts you have made to access habitats and to collect and import/export your samples in a responsible manner and in compliance with local, national and international laws, noting any permits that were obtained (give the name of the issuing authority, the date of issue, and any identifying information).*
Disturbance	*Describe any disturbance caused by the study and how it was minimized.*

Reporting for specific materials, systems and methods

We require information from authors about some types of materials, experimental systems and methods used in many studies. Here, indicate whether each material, system or method listed is relevant to your study. If you are not sure if a list item applies to your research, read the appropriate section before selecting a response.

Materials & experimental systems

n/a	Involved in the study	
☐	☒	Antibodies
☐	☒	Eukaryotic cell lines
☒	☐	Palaeontology and archaeology
☐	☒	Animals and other organisms
☒	☐	Human research participants
☒	☐	Clinical data
☒	☐	Dual use research of concern

Methods

n/a	Involved in the study	
☒	☐	ChIP-seq
☐	☒	Flow cytometry
☒	☐	MRI-based neuroimaging

Antibodies

Antibodies used	For the list of used antibodies, also see Supplemental Table 8 and 17.<br>B220-APC Biolegend RA3-6B2 Cat#103212 1:50<br>B220-BV650 Biolegend RA3-6B2 Cat#103241 1:100<br>B220-PE/Cy7 Biolegend RA3-6B2 Cat#103222 1:200<br>CD19-BUV661 BD Biosciences 1D3 Cat#563557 1:100<br>CD19-PE BD Biolegend 1D3 Cat# 152408 1:200<br>CD19-PerCP/Cy5.5 Biolegend 1D3 Cat#152406 1:200<br>Isotype control-PerCP/Cy5.5 Biolegend RTK2758 Cat#400532 1:200<br>human IgM-PE/CF594 BD Biosciences G20-127 Cat#562539 1:100<br>mouse IgM-PE/CF594 BD Biosciences R6-60.2 Cat#562565 1:100<br>human IgD-PE/Cy7 Biolegend IA6-2 Cat#348209 1:100<br>mouse IgD-PE/Cy7 Biolegend 11-26c.2a Cat#405719 1:100<br>human IgG-PE/Cy7 Biolegend M1310G05 Cat#410721 1:100<br>mouse IgG-BV421 Jackson ImmunoResearch Goat poly Cat#115-675-071 1:200<br>human Ig kappa-biotin Biolegend MHK-49 Cat#316504 1:500<br>Isotype control-biotin Biolegend MOPC-21 Cat#400103 1:500<br>mouse Ig kappa-biotin Biolegend RMK-12 Cat# 407204 1:500<br>Isotype control-biotin BD Biosciences R3-34 Cat#553923 1:500<br>mouse Ig lambda-BV650 BD Biosciences R26-46 Cat#744526 1:200<br>Isotype control BV650 BD Biosciences R35-95 Cat#563236 1:200<br>GL7-Alexa Fluor 647 Biolegend GL7 Cat#144605 1:100<br>GL7-PE Biolegend GL7 Cat#144608 1:200<br>GL7-Pacific Blue Biolegend GL7 Cat#144614 1:500<br>CD35-biotin BD Biosciences 8C12 Cat#553816 1:100<br>CD38-BV650 BD Biosciences 90/CD38 Cat#740489 1:200<br>CD38-PE/Dazzle594 Biolgend 90/CD38 Cat# 102730 1:200<br>CXCR4-PE/Dazzle594 Biolegend L276F12 Cat#146510 1:200<br>CD86-PE/Cy7 Biolegend GL-1 Cat#146510 1:200<br>CD93-BUV661 BD Biosciences AA4.1 Cat# 741574 1:200<br>Isotype control-BUV661 BD Biosciences R35-38 Cat#612978 1:200<br>CD21-PE/Cy7 Biolegend 7E9 Cat#123420 1:200<br>CD23-BV650 BD Biosciences B3B4 Cat#740456 1:200<br>CD43-PE/Dazzle594 Biolegend S11 Cat# 143218 1:200<br>CD138-PE/Cy7 Biolegend 281-2 Cat#142514 1:100<br>TACI-PE Biolegend 8F10 Cat#133403 1:100<br>CD5-PE/Cy5 Biolegend 53-7.3 Cat#100610 1:200<br><br>hIg mu Goat anti-Human IgM Bethyl Laboratories A80-100A-11 1:100<br>hIg mu Goat anti-Human IgMBethyl Laboratories A80-100P-37 1:75000<br>hIg gamma Goat anti-human IgG-FcBethyl Laboratories A80-104A-9 1:100<br>hIg gamma Goat anti-human IgG-FcBethyl Laboratories A80-104P-90 1:150000<br>hIg kappa Goat anti-Human Ig kappaBethyl Laboratories A80-115A-6 1:300<br>hIg kappa Goat anti-Human Ig kappaBethyl Laboratories A80-115P-43 1:150000<br>hIg alpha Goat anti-Human IgABethyl Laboratories A80-102A-6 1:100<br>hIg alpha Goat anti-Human IgABethyl Laboratories A80-102P-26 1:75000<br>hIg epsilon Goat anti-Human IgEBethyl Laboratories A80-108A-15 1:100<br>hIg epsilon Goat anti-Human IgEBethyl Laboratories A80-108P-35 1:75000<br>mIg mu Goat anti-Mouse IgMBethyl Laboratories A90-101A-22 1:100<br>mIg mu Goat anti-Mouse IgMBethyl Laboratories A90-101P-34 1:75000<br>mIg gamma Goat anti-Mouse IgG-FcBethyl Laboratories A90-131A-16 1:100<br>mIg gamma Goat anti-Mouse IgG-FcBethyl Laboratories A90-131P-38 1:100000<br>mIg kappa Goat anti-Mouse Ig kappaBethyl Laboratories A90-119A-14 1:100<br>mIg kappa Goat anti-Mouse Ig kappaBethyl Laboratories A90-119P 1:100000<br>mIg lambda Goat anti-Mouse Ig lambdaBethyl Laboratories A90-121A-12 1:100<br>mIg lambda Goat anti-Mouse Ig lambdaBethyl Laboratories A90-121P-18 1:100000<br>Goat Anti-Human IgG H&L (HRP) preadsorbed Abcam Cat#97175 1:400
Validation	All antibodies are commercially available and validated by manufactures for the applications used in this study.<br>See manufactures websites for validation statements (https://www.biolegend.com/; https://www.bdbiosciences.com/en-us; https://www.bethyl.com/;https://www.jacksonimmuno.com/).<br>B220-APC Biolegend RA3-6B2 Cat#103212 1:50<br>https://www.biolegend.com/ja-jp/products/apc-anti-mouse-human-cd45r-b220-antibody-442?Clone=RA3-6B2<br>Isotype Control<br>APC Rat IgG2a, κ Isotype Ctrl<br>Reactivity<br>Mouse, Human, Cross-Reactivity: Cat (Feline)

Antibody Type
Monoclonal
Host Species
Rat
Immunogen
Abelson murine leukemia virus-induced pre-B tumor cells
Concentration
0.2 mg/ml
Application
FC - Quality tested
Application Notes
Clone RA3-6B2 has been described to react with an epitope on the extracellular domain of the transmembrane CD45 glycoprotein which is dependent upon the expression of exon A and specific carbohydrate residues. Additional reported applications (for the relevant formats) include: immunoprecipitation1, in vitro and in vivo modulation of B cell responses2-4, and immunohistochemistry of acetone-fixed frozen sections and formalin-fixed paraffin-embedded sections5,6.
RRID
AB_312996 (BioLegend Cat. No. 103211)
AB_312997 (BioLegend Cat. No. 103212)

B220-BV650 Biolegend RA3-6B2 Cat#103241 1:100
https://www.biolegend.com/ja-jp/products/brilliant-violet-650-anti-mouse-human-cd45r-b220-antibody-7844?Clone=RA3-6B2
Isotype Control
Brilliant Violet 650™ Rat IgG2a, κ Isotype Ctrl
Reactivity
Mouse, Human, Cross-Reactivity: Cat (Feline)
Antibody Type
Monoclonal
Host Species
Rat
Immunogen
Abelson murine leukemia virus-induced pre-B tumor cells
Application
FC - Quality tested
Application Notes
Clone RA3-6B2 has been described to react with an epitope on the extracellular domain of the transmembrane CD45 glycoprotein which is dependent upon the expression of exon A and specific carbohydrate residues. Additional reported applications (for the relevant formats) include: immunoprecipitation1, in vitro and in vivo modulation of B cell responses2-4, and immunohistochemistry of acetone-fixed frozen sections and formalin-fixed paraffin-embedded sections5,6.
RRID
AB_11204069 (BioLegend Cat. No. 103241)

B220-PE/Cy7 Biolegend RA3-6B2 Cat#103222 1:200
https://www.biolegend.com/ja-jp/products/pe-cyanine7-anti-mouse-human-cd45r-b220-antibody-1930?Clone=RA3-6B2
Isotype Control
PE/Cyanine7 Rat IgG2a, κ Isotype Ctrl
Reactivity
Mouse, Human, Cross-Reactivity: Cat (Feline)
Antibody Type
Monoclonal
Host Species
Rat
Immunogen
Abelson murine leukemia virus-induced pre-B tumor cells
Application
FC - Quality tested
Recommended Usage
Each lot of this antibody is quality control tested by immunofluorescent staining with flow cytometric analysis. For flow cytometric staining, the suggested use of this reagent is ≤ 0.25 µg per 106 cells in 100 µl volume. It is recommended that the reagent be titrated for optimal performance for each application.
Application Notes
Clone RA3-6B2 has been described to react with an epitope on the extracellular domain of the transmembrane CD45 glycoprotein which is dependent upon the expression of exon A and specific carbohydrate residues. Additional reported applications (for the relevant formats) include: immunoprecipitation1, in vitro and in vivo modulation of B cell responses2-4, and immunohistochemistry of acetone-fixed frozen sections and formalin-fixed paraffin-embedded sections5,6.
RRID
AB_313004 (BioLegend Cat. No. 103221)
AB_313005 (BioLegend Cat. No. 103222)

CD19-BUV661 BD Biosciences 1D3 Cat#563557 1:100
https://www.bdbiosciences.com/ja-jp/products/reagents/flow-cytometry-reagents/research-reagents/single-color-antibodies-ruo/

buv395-rat-anti-mouse-cd19.563557
The 1D3 antibody reacts with CD19, a B lymphocyte-lineage differentiation antigen. CD19, a 95-kDa transmembrance glycoprotein, is a member of the immunoglobulin superfamily and is expressed throughout B-lymphocyte development from the pro-B cell through the mature B-cell stages. Terminally differentiated plasma cells do not express CD19. On the surface of mature B cells, the CD19 molecule associates with CD21 (CR-2) and CD81 (TAPA-1), and this multimolecular complex synergizes with surface immunoglobulin to promote cellular activation. Studies with CD19-deficient mice have suggested that the level of CD19 expression affects the generation and maturation of B cells in the bone marrow and periphery. B-1 lineage B cells, also known as CD5+ B cells, are drastically reduced or absent in CD19-deficient mice. Increased levels of CD19 expression correlate with increased frequencies of peritonal and splenic B-1 cells and reduced numbers of conventional B lymphocytes in the periphery. CD19 participates in B-lymphocyte development, B-cell activation, maturation of memory B cells and regulation of tolerance. CD19 has also been detected on peritoneal mast cells, co-localized with CD21/CD35, and it is proposed to play a role in complement-mediated mast-cell activation.

Reactivity:Mouse (QC Testing)
Isotype:Rat LEW, also known as Lewis IgG2a, κ
Immunogen:Mouse CD19 Transfected Cell Line
Application:Flow cytometry (Routinely Tested)
Entrez Gene ID:12478
RRID:AB_2722495

CD19-PE BD Biolegend 1D3 Cat# 152408 1:200
https://www.biolegend.com/ja-jp/products/pe-anti-mouse-cd19-antibody-13641?Clone=1D3/CD19
Isotype Control
PE Rat IgG2a, κ Isotype Ctrl
Reactivity
Mouse
Antibody Type
Monoclonal
Host Species
Rat
Immunogen
Transfected k562 cells expressing the extracellular domain of murine CD19.
Application
FC - Quality tested
Application Notes
Additional reported applications (for the relevant formats) include: blocking1,2 and immunoprecipitation1.
Application References
(PubMed link indicates BioLegend citation)
1. Krop I, et al. 1996. Eur. J. Immunol. 26:238. (FC, Blocking, IP)
2. Abraham P, et al. 2014. Clin. Exp. Immunol. 175:181. (Blocking)
RRID
AB_2629816 (BioLegend Cat. No. 152407)
AB_2629817 (BioLegend Cat. No. 152408)

CD19-PerCP/Cy5.5 Biolegend 1D3 Cat#152406 1:200
https://www.biolegend.com/ja-jp/products/percp-cyanine5-5-anti-mouse-cd19-antibody-13640?Clone=1D3/CD19
Isotype Control
PerCP/Cyanine5.5 Rat IgG2a, κ Isotype Ctrl
Reactivity
Mouse
Antibody Type
Monoclonal
Host Species
Rat
Immunogen
Transfected k562 cells expressing the extracellular domain of murine CD19.
Application
FC - Quality tested
RRID
AB_2629814 (BioLegend Cat. No. 152405)
AB_2629815 (BioLegend Cat. No. 152406)

Isotype control-PerCP/Cy5.5 Biolegend RTK2758 Cat#400532 1:200
https://www.biolegend.com/ja-jp/products/percp-cyanine5-5-rat-igg2a-kappa-isotype-ctrl-4201?Clone=RTK2758
Reactivity
KLH
Antibody Type
Monoclonal
Host Species
Rat

Immunogen
Trinitrophenol + KLH
Application
FC, ICFC - Quality tested
Application Notes
Additional reported applications (for the relevant formats) include: Intracellular Flow Cytometry (ICFC), Immunocytochemistry (ICC), Immunohistochemistry (IHC), Immunoprecipitation (IP), Western Blotting (WB), and Functional Assay (FA).
RRID
AB_2864286 (BioLegend Cat. No. 400531)

human IgM-PE/CF594 BD Biosciences G20-127 Cat#562565 1:100
https://www.bdbiosciences.com/ja-jp/products/reagents/flow-cytometry-reagents/research-reagents/single-color-antibodies-ruo/pe-cf594-rat-anti-mouse-igm.562565
R6-60.2
The R6-60.2 antibody monoclonal antibody specifically binds to mouse Immunoglobulin M (IgM) of Igh-C[a] and Igh-C[b] haplotypes. It does not react with other Ig isotypes. The R6-60.2 antibody has not been shown to stimulate B-cell proliferation.
Reactivity:Mouse (QC Testing)
Isotype:Rat LOU, also known as Louvain, LOU/C, LOU/M IgG2a, κ
Immunogen:Pooled Mouse Ig
Application:Flow cytometry (Routinely Tested)
RRID:AB_2737658

mouse IgM-PE/CF594 BD Biosciences R6-60.2 Cat#562539 1:100
https://www.bdbiosciences.com/ja-jp/products/reagents/flow-cytometry-reagents/research-reagents/single-color-antibodies-ruo/pe-cf594-mouse-anti-human-igm.562539
G20-127
IgM is an important component in the first line of defense against foreign pathogens, but may also play a role in autoimmune diseases. IgM monomers consist of two light and two heavy chains. Unlike the heavy chain of an IgG antibody which contains 3 constant Ig domains, the μ heavy chain of IgM contains 4 constant Ig domains. Five IgM monomers complex with a small polypeptide (J-chain) to form pentameric IgM that can be found in human plasma. In an immune response, the binding of IgM to a cell surface antigen enables C1q to activate interactions with downstream components in the classical complement pathway. Mature B lymphocytes express IgM. The G20-127 monoclonal antibody binds to the heavy chain of human IgM. The G20-127 antibody is not thought to react with other immunoglobulin heavy chain isotypes.
Reactivity:Human (QC Testing)
Isotype:Mouse IgG1, κ
Application:Flow cytometry (Routinely Tested)
Vol. Per Test:5 μl
Entrez Gene ID:3507
RRID:AB_2737641

human IgD-PE/Cy7 Biolegend IA6-2 Cat#348209 1:100
https://www.biolegend.com/ja-jp/products/pe-cyanine7-anti-human-igd-antibody-6996?Clone=IA6-2
Isotype Control
PE/Cyanine7 Mouse IgG2a, κ Isotype Ctrl
Reactivity
Human
Antibody Type
Monoclonal
Host Species
Mouse
Immunogen
Human IgD
Application
FC - Quality tested
Application Notes
Additional reported applications (for the relevant formats) include: immunohistochemical staining of paraformaldehyde fixed frozen sections.4
Application References
(PubMed link indicates BioLegend citation)
1. Chen K, et al. 2009. Nat. Immunol. 10:889.
2. Lee CH, et al. 2005. J. Exp. Med. 203:63.
3. Sutter JA, et al. 2008. Clin. Immunol. 126:282.
4. Li H and Pauza CD. 2015. Eur. J. Immunol. 45:298. (IHC)
RRID
AB_10683460 (BioLegend Cat. No. 348209)
AB_10680462 (BioLegend Cat. No. 348210)

mouse IgD-PE/Cy7 Biolegend 11-26c.2a Cat#405719 1:100
https://www.biolegend.com/ja-jp/products/pe-cyanine7-anti-mouse-igd-8398?Clone=11-26c.2a
Isotype Control

PE/Cyanine7 Rat IgG2a, κ Isotype Ctrl
Reactivity
Mouse
Antibody Type
Monoclonal
Host Species
Rat
Application
FC - Quality tested
Application Notes
The 11-26c.2a antibody reacts with immunoglobulin D in all tested mouse haplotypes. The antibody binds membrane IgD expressed on most B cells. The 11-26c.2a antibody neither induces proliferation of splenic B cells nor induces B cell activation. Additional reported applications (for the relevant formats) include: immunohistochemical staining of acetone-fixed frozen sections2,3.
Application References
(PubMed link indicates BioLegend citation)
1. Nitschke L, et al. 1993. P. Natl. Acad. Sci. USA 90:1887. (FC)
2. Weih D, et al. 2001. J. Immunol. 167:1909. (IHC)
3. Koni PA, et al. 2001. J. Exp. Med. 193:741. (IHC)
4. Ahuja A, et al. 2007. J. Immunol. 179:3351. (FC) PubMed
5. Haynes NM, et al. 2007. J. Immunol. 179:5099. (FC)
6. Good-Jacobson KL, et al. 2010. Nat. Immunol. 11:535. (FC) PubMed
7. Tomayko MM, et al. 2010. J. Immunol. 185:7146. PubMed
8. Park SY, et al. 2013. J. Immunol. 190:1094. PubMed
9. Rouaud P, et al. 2014. J Exp Med. 211:975. PubMed
RRID
AB_2561875 (BioLegend Cat. No. 405719)
AB_2561876 (BioLegend Cat. No. 405720)

human IgG-PE/Cy7 Biolegend M1310G05 Cat#410721 1:100
https://www.biolegend.com/ja-jp/products/pe-cyanine7-anti-human-igg-fc-antibody-16288?Clone=M1310G05
Isotype Control
PE/Cyanine7 Rat IgG2a, κ Isotype Ctrl
Reactivity
Human
Antibody Type
Monoclonal
Host Species
Rat
Immunogen
Human Siglec-E-IgG Fc fusion protein.
Application
FC - Quality tested
Application Notes
Clone M1310G05 recognizes IgG in the membrane of memory B cells, has a stronger affinity for IgG1 and IgG3 than for IgG2 and IgG4, and does not cross react with IgD, IgE, or IgM.
RRID
AB_2750226 (BioLegend Cat. No. 410721)
AB_2750227 (BioLegend Cat. No. 410722)

mouse IgG-BV421 Jackson ImmunoResearch Goat poly Cat#115-675-071 1:200
https://www.jacksonimmuno.com/catalog/products/115-675-071
Target: Mouse
Host: Goat
Antibody Format: Whole IgG
Specificity: IgG, Fcγ fragment specific
Minimal Cross Reactivity: Human, Bovine, Horse Serum Proteins
Conjugate: Brilliant Violet 421™
Product Category: Brilliant Violet™ Conjugates for Multiple Labeling
Clonality: Polyclonal
RRID: AB_2651084
Based on immunoelectrophoresis and/or ELISA, the antibody reacts with the Fc portion of mouse IgG heavy chain but not with the Fab portion of mouse immunoglobulins. No antibody was detected against mouse IgM or non-immunoglobulin serum proteins. The antibody has been tested by ELISA and/or solid-phase adsorbed to ensure minimal cross-reaction with human, bovine and horse serum proteins, but it may cross-react with immunoglobulins from other species.

Whole IgG antibodies are isolated as intact molecules from antisera by immunoaffinity chromatography. They have an Fc portion and two antigen binding Fab portions joined together by disulfide bonds and therefore they are divalent. The average molecular weight is reported to be about 160 kDa. The whole IgG form of antibodies is suitable for the majority of immunodetection procedures and is the most cost effective.

human Ig kappa-biotin Biolegend MHK-49 Cat#316504 1:500
https://www.biolegend.com/ja-jp/products/biotin-anti-human-ig-light-chain-kappa-antibody-3296?Clone=MHK-49
The MHK-49 antibody reacts with both soluble and membrane human immunoglobulin light chain kappa (κ). It does not react with human immunoglobulin light chain lambda (λ) or heavy chain. The MHK-49 antibody can be used as primary or secondary reagent for immunofluorescent staining or ELISA analysis.
Isotype Control
Biotin Mouse IgG1, κ Isotype Ctrl
Reactivity
Human, Cynomolgus
Antibody Type
Monoclonal
Host Species
Mouse
Immunogen
Human Ig cocktail
Application
FC - Quality tested
ELISA - Reported in the literature, not verified in house
Application References
(PubMed link indicates BioLegend citation)
1. Lockridge JL, et al. 2013. Biol. Blood Marrow Transplant 9:1310-22. (ELISA)
RRID
AB_493609 (BioLegend Cat. No. 316504)

Isotype control-biotin Biolegend MOPC-21 Cat#400103 1:500
https://www.biolegend.com/ja-jp/products/biotin-mouse-igg1-kappa-isotype-ctrl-1405?Clone=MOPC-21
The MOPC-21 immunoglobulin has unknown specificity. The isotype of this antibody is mouse IgG1, κ. This antibody was chosen as an isotype control after screening on a variety of resting, activated, live, and fixed mouse, rat and human tissues.
Antibody Type
Monoclonal
Host Species
Mouse
Application
FC, ICFC - Quality tested
Application Notes
Additional reported applications (for the relevant formats) include: Intracellular Flow Cytometry (ICFC), Immunocytochemistry (ICC), Immunohistochemistry (IHC), Immunoprecipitation (IP), Western Blotting (WB), Functional Assay (FA)
Application References
(PubMed link indicates BioLegend citation)
1. Carlsten M, et al. 2007. Cancer Res. 67:1317. PubMed
2. Smed-Sörensen A, et al. 2008. Blood 111:5037. PubMed (FA)
3. Bunesmann MM, et al. 2011. Am. J. Respir. Cell. Mol. Biol. Epub. PubMed
4. Matsuyama T, et al. 2005. Infect. Immun. 73:1044. (IF)
5. Correia DV, et al. 2011. Blood 118:992. (FC) PubMed
6. Lian IA, et al. 2011. Placenta. 32:823. PubMed
7. Bufe B, et al. 20015. J Biol Chem. 290:7369. PubMed

mouse Ig kappa-biotin Biolegend RMK-12 Cat# 407204 1:500

https://www.biolegend.com/ja-jp/products/biotin-anti-mouse-ig-light-chain-kappa-2630?Clone=RMK-12
The RMK-12 monoclonal antibody reacts with immunoglobulin light chain kappa in all tested mouse haplotype (Igh-a and b). It does not react with lambda chain. The RMK-12 monoclonal antibody may be used as a primary antibody for ELISA.
Reactivity
Mouse
Antibody Type
Monoclonal
Host Species
Rat
Immunogen
Mouse Ig cocktail
Application
ELISA, FC
RRID
AB_345328 (BioLegend Cat. No. 407204)

Isotype control-biotin BD Biosciences R3-34 Cat#553923 1:500
https://www.bdbiosciences.com/ja-jp/products/reagents/flow-cytometry-reagents/research-reagents/flow-cytometry-controls-and-lysates/biotin-rat-igg1-isotype-control.553923

R3-34
Following immunization of a rat with mouse immunoglobulin (Ig), the Ig from the R3-34 hybridoma was identified as a non-reactive clone. The R3-34 immunoglobulin was selected as an Ig isotype control following screening for low background staining on a variety of mouse and human cells and tissues.
Isotype:Rat IgG1, κ
Immunogen:Mouse immunoglobulin
Application:Flow cytometry, Isotype control (Routinely Tested)
RRID:AB_395138

mouse Ig lambda-BV650 BD Biosciences R26-46 Cat#744526 1:200
https://www.bdbiosciences.com/ja-jp/products/reagents/flow-cytometry-reagents/research-reagents/single-color-antibodies-ruo/bv650-rat-anti-mouse-ig-1-2-3-light-chain.744526
R26-46
The R26-46 antibody reacts specifically with mouse Igs bearing λ1, λ2, or λ3 light chains. It does not react with κ light chain or heavy chain. Detection of surface immunoglobulin on Ig λ chain-secreting hybridoma cells has been demonstrated with R26-46 mAb.
Reactivity:Mouse (Tested in Development)
Isotype:Rat IgG2a, κ
Immunogen:Pooled Mouse Ig
Application:Flow cytometry (Qualified)
Entrez Gene ID:111519
RRID:AB_2742300

Isotype control BV650 BD Biosciences R35-95 Cat#563236 1:200
https://www.bdbiosciences.com/ja-jp/products/reagents/flow-cytometry-reagents/research-reagents/flow-cytometry-controls-and-lysates/bv650-rat-igg2a-isotype-control.563236
R35-95
The R35-95 hybridoma was generated by hybridization of Y3 myeloma cells with spleen cells from LOU rats immunized with mouse immunoglobulins. The R35-95 hybridoma produces rat IgG2a, κ immunoglobulin that has no measurable reactivity with mouse immunoglobulins. The R35-95 immunoglobulin was selected as an isotype control following screening for low background binding on a variety of mouse and human tissues.
Isotype:Rat LOU, also known as Louvain, LOU/C, LOU/M IgG2a, κ
Immunogen:Mouse Pooled Immunoglobulin
Application:Flow cytometry, Isotype control (Routinely Tested)
RRID:AB_2869472

GL7-Alexa Fluor 647 Biolegend GL7 Cat#144605 1:100
https://www.biolegend.com/ja-jp/products/alexa-fluor-647-anti-mouse-human-gl7-antigen-t-and-b-cell-activation-marker-antibody-8602?Clone=GL7
Isotype Control
Alexa Fluor® 647 Rat IgM, κ Isotype Ctrl
Reactivity
Mouse, Human
Antibody Type
Monoclonal
Host Species
Rat
Immunogen
LPS activated DBA/J mouse B cells
Application
FC - Quality tested
IHC-F - Verified
Application Notes
The GL7 antibody does not block the binding of CD22 with sulfated a2-6-sialyl-LacNAc.
Cross-reactivity to ferret has been reported by a collaborator, but not verified in house.
Application References
(PubMed link indicates BioLegend citation)
1. Laszlo G, et al. 1993. J. Immunol. 150:5252. (FC, IP)
2. Hartgring SA, et al. 2012. Arthritis Res. Ther. 14:R137. (FC)
3. Taylor JJ, et al. 2012. J. Exp. Med. 209:597. (FC, IHC)
4. Balogh A, et al. 2010. Immunol. Lett. 130:89. (IHC)
5. Kimura N, et al. 2007. J. Biol. Chem. 282:32200. (ELISA, FC)
RRID
AB_2562184 (BioLegend Cat. No. 144605)
AB_2562185 (BioLegend Cat. No. 144606)

GL7-PE Biolegend GL7 Cat#144608 1:200
https://www.biolegend.com/ja-jp/products/pe-anti-mouse-human-gl7-antigen-t-and-b-cell-activation-marker-antibody-9122?Clone=GL7
Isotype Control

PE Rat IgM, κ Isotype Ctrl
Reactivity
Mouse, Human
Antibody Type
Monoclonal
Host Species
Rat
Immunogen
LPS activated DBA/J mouse B cells
Application
FC - Quality tested
Application Notes
The GL7 antibody does not block the binding of CD22 with sulfated a2-6-sialyl-LacNAc.
Cross-reactivity to ferret has been reported by a collaborator, but not verified in house.
Application References
(PubMed link indicates BioLegend citation)
1. Laszlo G, et al. 1993. J. Immunol. 150:5252. (FC, IP)
2. Hartgring SA, et al. 2012. Arthritis Res. Ther. 14:R137. (FC)
3. Taylor JJ, et al. 2012. J. Exp. Med. 209:597. (FC, IHC)
4. Balogh A, et al. 2010. Immunol. Lett. 130:89. (IHC)
5. Kimura N, et al. 2007. J. Biol. Chem. 282:32200. (ELISA, FC)
RRID
AB_2562925 (BioLegend Cat. No. 144607)
AB_2562926 (BioLegend Cat. No. 144608)

GL7-Pacific Blue Biolegend GL7 Cat#144614 1:500
Isotype Control
Pacific Blue™ Rat IgM, κ Isotype Ctrl
Reactivity
Mouse, Human
Antibody Type
Monoclonal
Host Species
Rat
Immunogen
LPS activated DBA/J mouse B cells
Application
FC - Quality tested
Application Notes
The GL7 antibody does not block the binding of CD22 with sulfated a2-6-sialyl-LacNAc.
Cross-reactivity to ferret has been reported by a collaborator, but not verified in house.
Application References
(PubMed link indicates BioLegend citation)
1. Laszlo G, et al. 1993. J. Immunol. 150:5252. (FC, IP)
2. Hartgring SA, et al. 2012. Arthritis Res. Ther. 14:R137. (FC)
3. Taylor JJ, et al. 2012. J. Exp. Med. 209:597. (FC, IHC)
4. Balogh A, et al. 2010. Immunol. Lett. 130:89. (IHC)
5. Kimura N, et al. 2007. J. Biol. Chem. 282:32200. (ELISA, FC)
RRID
AB_2563291 (BioLegend Cat. No. 144613)
AB_2563292 (BioLegend Cat. No. 144614)

CD35-biotin BD Biosciences 8C12 Cat#553816 1:100
https://www.bdbiosciences.com/ja-jp/products/reagents/flow-cytometry-reagents/research-reagents/single-color-antibodies-ruo/biotin-rat-anti-mouse-cd35.553816
8C12
The 8C12 antibody recognizes an epitope present on the 190-kDa complement receptor protein, originally designated CR1 (CD35), but not the 145-150-kDa CR2 (CD21) molecule. Unlike the human system, in which these proteins are products of independent genes, both of these mouse receptors are membrane proteins resulting from the alternative splicing of mRNA transcribed from the Cr2 gene. Therefore, an alternative nomenclature has been proposed, designating the proteins Cr2-190 (CD21b) and Cr2-145 (CD21a), respectively. The epitope recognized by 8C12 mAb is only present on CD35/CD21b. Moreover, it has also been proposed that Crry is the true mouse genetic homologue of human CR1 (CD35). In the mouse, CD35 is expressed on the majority of peripheral B cells, on the majority of resident peritoneal macrophages, on peripheral blood granulocytes after treatment with N-formyl-Met-Leu-Phe, and on follicular dendritic cells, but not on thymocytes, T cells, erythrocytes, or platelets. In addition, it has not been detected, at the protein or mRNA level, in the macrophage cell line J774, bone marrow-derived macrophages, or thioglycollate-elicited peritoneal macrophages. The 8C12 mAb has been reported to inhibit rosette formation by C3bbearing sheep erythrocytes, to block the complement-dependent trapping of immune complexes by follicular dendritic cells, and to down-regulate mouse CD35 expression upon in vivo application, inhibiting only some primary antibody responses to immunization. B lymphocytes of Cr2[null] mice display impaired humoral immune responses in vivo. The 8C12 mAb recognizes an epitope on mouse CD35 distinct from the epitope recognized by anti-mouse CD21/CD35 mAb 7G6, and it does not block binding by 7G6 mAb to CD35.

*Please note that the isotype of 8C12 mAb was originally reported to be Rat IgG2c. Further investigations have demonstrated that the isotype of 8C12 mAb is Rat IgG2a.
Reactivity:Mouse (QC Testing)
Isotype:Rat SD, also known as Sprague-Dawley (outbred) IgG2a, κ
Immunogen:Purified mouse CR1
Application:Flow cytometry (Routinely Tested), Immunohistochemistry-frozen (Reported)
Entrez Gene ID:12902
RRID:AB_395068

CD38-BV650 BD Biosciences 90/CD38 Cat#740489 1:200
https://www.bdbiosciences.com/ja-jp/products/reagents/flow-cytometry-reagents/research-reagents/single-color-antibodies-ruo/bv650-rat-anti-mouse-cd38.740489
90/CD38
The 90 monoclonal antibody specifically binds to CD38, a 42 kDa transmembrane glycoprotein on immature and mature, resting and activated, B lymphocytes. In contrast to humans, CD38 expression is down-regulated on mouse germinal center B cells and plasma cells. CD38 is also expressed on a subpopulation of thymic and peripheral T cells, NK cells, and splenic macrophages. Furthermore, CD38 has been detected on bone marrow-derived hematopoietic stem cells. The CD38 molecule is reported to exhibit both cyclase and hydrolase activities and plays a role in lymphocyte activation. CD31, both human and mouse, is reported to be a ligand for human CD38.
Reactivity:Mouse (Tested in Development)
Isotype:Rat IgG2a, κ
Immunogen:Mouse Bone Marrow Pre-B cells
Application:Flow cytometry (Qualified)
Entrez Gene ID:12494
RRID:AB_2740212

CD38-PE/Dazzle594 Biolgend 90/CD38 Cat#102730 1:200
https://www.biolegend.com/ja-jp/products/pe-dazzle-594-anti-mouse-cd38-antibody-13852?Clone=90
Isotype Control
PE/Dazzle™ 594 Rat IgG2a, κ Isotype Ctrl
Reactivity
Mouse
Antibody Type
Monoclonal
Host Species
Rat
Immunogen
Mouse bone marrow pre-B cells
Application
FC - Quality tested
RRID
AB_2632890 (BioLegend Cat. No. 102729)
AB_2632891 (BioLegend Cat. No. 102730)

CXCR4-PE/Dazzle594 Biolegend L276F12 Cat#146510 1:200
https://www.biolegend.com/ja-jp/products/percp-cyanine5-5-anti-mouse-cd184-cxcr4-antibody-9059?Clone=L276F12
Isotype Control
PerCP/Cyanine5.5 Rat IgG2b, κ Isotype Ctrl
Reactivity
Mouse
Antibody Type
Monoclonal
Host Species
Rat
Immunogen
Mouse CXCR4-transfected cells
Application
FC - Quality tested
Application Notes
Additional reported applications (for the relevant formats) include: in vivo blocking1
Application References
(PubMed link indicates BioLegend citation)
1. Costa MJ, et al. 2018. PLoS One. 13:e0194688 (Block) PubMed
RRID
AB_2562786 (BioLegend Cat. No. 146509)
AB_2562787 (BioLegend Cat. No. 146510)

CD86-PE/Cy7 Biolegend GL-1 Cat#146510 1:200
https://www.biolegend.com/ja-jp/products/percp-cyanine5-5-anti-mouse-cd184-cxcr4-antibody-9059?Clone=L276F12

Isotype Control
PerCP/Cyanine5.5 Rat IgG2b, κ Isotype Ctrl
Reactivity
Mouse
Antibody Type
Monoclonal
Host Species
Rat
Immunogen
Mouse CXCR4-transfected cells
Application
FC - Quality tested
Application Notes
Additional reported applications (for the relevant formats) include: in vivo blocking1
Application References
(PubMed link indicates BioLegend citation)
1. Costa MJ, et al. 2018. PLoS One. 13:e0194688 (Block) PubMed
RRID
AB_2562786 (BioLegend Cat. No. 146509)
AB_2562787 (BioLegend Cat. No. 146510)

CD93-BUV661 BD Biosciences AA4.1 Cat#741574 1:200
https://www.bdbiosciences.com/ja-jp/products/reagents/flow-cytometry-reagents/research-reagents/single-color-antibodies-ruo/buv661-rat-anti-mouse-cd93-early-b-lineage.741574
AA4.1
The AA4.1 monoclonal antibody specifically recognizes the Early B Lineage antigen which is also known as CD93, AA4 antigen, Ly-68, and Complement component C1q receptor (C1qRp). This 130-140-kDa type I transmembrane glycoprotein is expressed on immature B lymphocytes in the adult bone marrow and on hematopoietic progenitors and stem cells in adult bone marrow, fetal liver, and embryonic yolk sac. Although CD93+ cells are most plentiful in adult mouse bone marrow, a smaller number of CD93+ cells which express lower CD93 levels can be detected in the adult spleen using bright fluorescent conjugates of the AA4.1 antibody or an amplified indirect immunofluorescent staining procedure. It has been observed that the staining pattern of the 493 monoclonal antibody is similar to that of the AA4.1 antibody, in that both antibodies precipitate molecules of the same molecular weight. Staining with the AA4.1 antibody is not blocked by the 493 antibody. These results suggest that the antibodies recognize separate epitopes on the same Early B Lineage antigen.
Reactivity:Mouse (Tested in Development)
Isotype:Rat SD, also known as Sprague-Dawley (outbred) IgG2b, κ
Immunogen:Pre-B lymphoma 70Z/3, derived from (C57BL/6 x DBA/2)F1 mouse
Application:Flow cytometry (Qualified)
RRID:AB_2870995

Isotype control-BUV661 BD Biosciences R35-38 Cat#612978 1:200
https://www.bdbiosciences.com/ja-jp/products/reagents/flow-cytometry-reagents/research-reagents/flow-cytometry-controls-and-lysates/buv661-rat-igg2b-isotype-control.612978
R35-38
The R35-38 hybridoma produces monoclonal rat IgG2b, κ. The target molecule specificity of this clone is unknown. R35-38 has little or no reactivity with mammalian cells and has been found useful as an immunoglobulin isotype control.
Isotype:Rat IgG2b, κ
Application:Flow cytometry, Isotype control (Routinely Tested)
RRID:AB_2869647

CD21-PE/Cy7 Biolegend 7E9 Cat#123420 1:200
https://www.biolegend.com/ja-jp/products/pe-cyanine7-anti-mouse-cd21-cd35-cr2-cr1-antibody-6228?Clone=7E9
Isotype Control
PE/Cyanine7 Rat IgG2a, κ Isotype Ctrl
Reactivity
Mouse
Antibody Type
Monoclonal
Host Species
Rat
Immunogen
CD35/CFA
Application
FC - Quality tested

Application References
(PubMed link indicates BioLegend citation)
1. Boackle S, et al. 2001 Immunity 15:775.
2. de Andres B, et al. 2012. J. Immunol. 189:2300. PubMed
3. Chiu YK, et al. 2014. J Immunol. 193:2207. PubMed

4. Koening PA, et al. 2014. J Biol Chem. 289:34490. PubMed
RRID
AB_1953276 (BioLegend Cat. No. 123419)
AB_1953277 (BioLegend Cat. No. 123420)

CD23-BV650 BD Biosciences B3B4 Cat#740456 1:200
https://www.bdbiosciences.com/ja-jp/products/reagents/flow-cytometry-reagents/research-reagents/single-color-antibodies-ruo/bv650-rat-anti-mouse-cd23.740456
B3B4
The B3B4 monoclonal antibody specifically binds to CD23, the low affinity IgE Fc receptor (FcεRII) expressed on mature resting conventional B lymphocytes, but not on B-1 cells (CD5+ B cells) or T lymphocytes. It does not react with high-affinity IgE receptors, as demonstrated on mouse mast cell lines. The regulation of CD23 surface expression on activated B cells appears to be complex, depending upon the mode of activation and the presence of cytokines. IgE synthesis is negatively regulated by CD23, and CD23 expression is upregulated on splenocytes in the presence of IgE. CD23 is also upregulated on follicular dendritic cells in the lymph nodes of immunized mice, and a subset of splenic dendritic cells expresses CD23. The B3B4 antibody abrogates antigen-specific IgE-dependent modulation of immune responses in normal mice. This monoclonal antibody also blocks IgE binding and eosinophil infiltration in the lung of immunized mice. Different in vivo results have been obtained when using the intact B3B4 antibody or the F(ab')2 fragments. B3B4 mAb does not cross-react with rat or human IgE Fc Receptor.
Reactivity:Mouse (Tested in Development)
Isotype:Rat LOU, also known as Louvain, LOU/C, LOU/M IgG2a, κ
Immunogen:FcεR isolated from the mouse B hybridoma line O1.2B2
Application:Flow cytometry (Qualified)
Entrez Gene ID:14128
RRID:AB_2740183

CD43-PE/Dazzle594 Biolegend S11 Cat#143218 1:200
https://www.biolegend.com/ja-jp/products/pedazzle-594-anti-mouse-cd43-antibody-17487?Clone=S11
Isotype Control
PE/Dazzle™ 594 Rat IgG2b, κ Isotype Ctrl
Reactivity
Mouse
Antibody Type
Monoclonal
Host Species
Rat
Immunogen
Mouse plasmacytoma cells
Application
FC - Quality tested
Application Notes
Additional reported applications (for the relevant formats) include: Western blotting3. The S11 antibody reacts with pan-CD43.
Application References
(PubMed link indicates BioLegend citation)
1. Gaspari AA, et al. 1993. J. Invest. Dermatol. 100:247. (FC)
2. Merzaban JS, et al. 2005. J. Immunol. 174:4051. (FC)
3. Baecher-Allan CM, et al. 1993. Immunogenetics. 37:183. (WB)

CD138-PE/Cy7 Biolegend 281-2 Cat#142514 1:100
https://www.biolegend.com/ja-jp/products/pe-cyanine7-anti-mouse-cd138-syndecan-1-antibody-8601?Clone=281-2
Isotype Control
PE/Cyanine7 Rat IgG2a, κ Isotype Ctrl
Reactivity
Mouse
Antibody Type
Monoclonal
Host Species
Rat
Immunogen
Mouse mammary gland epithelial cell line NMuMG
Application
FC - Quality tested
Application Notes
Additional reported applications (for the relevant formats) include: immunohistochemical staining of frozen tissue3 and formalin-fixed paraffin embedded tissue4 and immunofluorescent staining2,3.
Application References
(PubMed link indicates BioLegend citation)
1. Jalkanen M, et al. 1985. J. Cell. Biol. 101:976. (FC)
2. Miettinen H, et al. 1994. J. Cell. Sci. 107:1571. (IF)
3. Li Q, et al. 2002. Cell 111:635. (IF, IHC)

4. McCarthy BA, et al. 2012. BMC Cancer. 12:203. (IHC)
RRID
AB_2562197 (BioLegend Cat. No. 142513)
AB_2562198 (BioLegend Cat. No. 142514)

TACI-PE Biolegend 8F10 Cat#133403 1:100
https://www.biolegend.com/ja-jp/products/pe-anti-mouse-cd267-taci-antibody-5807?Clone=8F10
Isotype Control
PE Rat IgG2a, κ Isotype Ctrl
Reactivity
Mouse
Antibody Type
Monoclonal
Host Species
Rat
Immunogen
Rat RBL transfected cell line
Application
FC - Quality tested

Application References
(PubMed link indicates BioLegend citation)
1. Siegel RM, et al. 2001. Nat. Immunol. 2:577.
2. Von Bulow GU, et al. 1997. Science 278:138.
3. Yan M, et al. 2001. Nat. Immunol. 2:638.
4. Diaz-de-Durana Y, et al. 2006. Blood 107:594.
5. Moir S, et al. 2012. Blood. 120:4850. PubMed.
RRID
AB_2203542 (BioLegend Cat. No. 133403)
AB_2240584 (BioLegend Cat. No. 133404)

CD5-PE/Cy5 Biolegend 53-7.3 Cat#100610 1:200
https://www.biolegend.com/ja-jp/products/pe-cyanine5-anti-mouse-cd5-antibody-161?Clone=53-7.3
Isotype Control
PE/Cyanine5 Rat IgG2a, κ Isotype Ctrl
Reactivity
Mouse
Antibody Type
Monoclonal
Host Species
Rat
Immunogen
Mouse thymus or spleen
Application
FC - Quality tested
Application Notes
Additional reported applications (for the relevant formats) include: immunoprecipitation1, and immunohistochemistry2 of acetone-fixed frozen tissue sections, zinc-fixed paraffin-embedded sections and formalin-fixed paraffin-embedded sections.
Application References
(PubMed link indicates BioLegend citation)
1. Ledbetter JA, et al. 1979. Immunol. Rev. 47:63. (IP)
2. Ledbetter JA, et al. 1980. J. Exp. Med. 152:280. (FC, IHC)
3. Bourdeau A, et al. 2007. Blood doi:10.1182/blood-2006-08-044370.
RRID
AB_312738 (BioLegend Cat. No. 100609)
AB_312739 (BioLegend Cat. No. 100610)

hIg mu Goat anti-Human IgM Bethyl Laboratories A80-100A-11 1:100
https://www.fortislife.com/products/immunoglobulins/goat-anti-human-igm-antibody/A80-100A
Target:Human IgM
Reactivity:Human
Application:ELISA, ICC, IHC, WB
Host:Goat
Clonality:Polyclonal
Format:Whole IgG
IsoType:IgG
Purity:Antigen Affinity Purified
Antiserum was solid phase adsorbed to ensure class specificity. The antibody was isolated by affinity chromatography using antigen coupled to agarose beads. Antibody concentration was determined by extinction coefficient: absorbance at 280 nm of 1.4 equals 1.0

mg of IgG. By immunoelectrophoresis and ELISA this antibody reacts specifically with human IgM. Cross reactivity with other immunoglobulins and light chains is less than 0.1%. This antibody may cross react with IgM from other species.
Western Blot 1:1,000 - 1:30,000
Immunohistochemistry 1:200 - 1:2,000
Immunocytochemistry 1:200 - 1:2,000
ELISA 1:1,000 - 1:30,000; for coating plates 1:100 - 1:500

hIg mu Goat anti-Human IgM Bethyl Laboratories A80-100P-37 1:75000
https://www.fortislife.com/products/secondary-antibodies/goat-anti-human-igm-antibody-hrp-conjugated/A80-100P
Target:Human IgM
Reactivity:Human
Application:ELISA, ICC, IHC, WB
Host:Goat
Clonality:Polyclonal
Format:Whole IgG
IsoType:IgG
Conjugate:HRP
Purity:Antigen Affinity Purified
Antiserum was solid phase adsorbed to ensure class specificity. The antibody was isolated by affinity chromatography using antigen coupled to agarose beads and conjugated to horseradish peroxidase (HRP). Antibody concentration was determined by extinction coefficient prior to conjugation: absorbance at 280 nm of 1.4 equals 1.0 mg of IgG. Molar enzyme/antibody protein ratio is 4:1. By immunoelectrophoresis and ELISA this antibody reacts specifically with human IgM. Cross reactivity with other immunoglobulins and light chains is less than 0.1%. This antibody may cross react with IgM from other species.
Western Blot 1:2,000 - 1:20,000
Immunohistochemistry 1:200 - 1:2,000
Immunocytochemistry 1:200 - 1:2,000
ELISA 1:10,000 - 1:100,000

hIg gamma Goat anti-human IgG-FcBethyl Laboratories A80-104A-9 1:100
https://www.fortislife.com/products/immunoglobulins/goat-anti-human-igg-fc-fragment-antibody/A80-104A
Target:Human IgG-Fc Fragment
Reactivity:Human
Application:ELISA, ICC, IHC, WB
Host:Goat
Clonality:Polyclonal
Format:Whole IgG
IsoType:IgG
Purity:Antigen Affinity Purified
Antiserum was solid phase adsorbed to ensure class specificity. The antibody was isolated by affinity chromatography using antigen coupled to agarose beads. Antibody concentration was determined by extinction coefficient: absorbance at 280 nm of 1.4 equals 1.0 mg of IgG. By immunoelectrophoresis and ELISA this antibody reacts specifically with human IgG. Cross reactivity with IgA, IgM and light chains is less than 1%. This antibody may cross react with IgG from other species.
Western Blot 1:1,000 - 1:30,000
Immunohistochemistry 1:200 - 1:2,000
Immunocytochemistry 1:200 - 1:2,000
ELISA 1:1,000 - 1:30,000; for coating plates 1:100 - 1:500

hIg gamma Goat anti-human IgG-FcBethyl Laboratories A80-104P-90 1:150000
https://www.fortislife.com/products/secondary-antibodies/goat-anti-human-igg-fc-fragment-antibody-hrp-conjugated/A80-104P
Target:Human IgG-Fc Fragment
Reactivity:Human
Application:ELISA, ICC, IHC, WB
Host:Goat
Clonality:Polyclonal
Format:Whole IgG
IsoType:IgG
Conjugate:HRP
Purity:Antigen Affinity Purified
Antiserum was solid phase adsorbed to ensure class specificity. The antibody was isolated by affinity chromatography using antigen coupled to agarose beads and conjugated to horseradish peroxidase (HRP). Antibody concentration was determined by extinction coefficient prior to conjugation: absorbance at 280 nm of 1.4 equals 1.0 mg of IgG. Molar enzyme/antibody protein ratio is 4:1. By immunoelectrophoresis and ELISA this antibody reacts specifically with human IgG. Cross reactivity with IgM, IgA and light chains is less than 1%. This antibody may cross react with IgG from other species.
Western Blot 1:5,000 - 1:50,000
Immunohistochemistry 1:200 - 1:5,000
Immunocytochemistry 1:200 - 1;5,000
ELISA 1:10,000 - 1:100,000

hIg kappa Goat anti-Human Ig kappaBethyl Laboratories A80-115A-6 1:300
https://www.fortislife.com/products/immunoglobulins/goat-anti-human-kappa-light-chain-antibody/A80-115A

Target:Human Kappa Light Chain
Reactivity:Human
Application:ELISA, ICC, IHC, WB
Host:Goat
Clonality:Polyclonal
Format:Whole IgG
IsoType:IgG
Purity:Antigen Affinity Purified
The antibody was isolated by affinity chromatography using antigen coupled to agarose beads. Antibody concentration was determined by extinction coefficient: absorbance at 280 nm of 1.4 equals 1.0 mg of IgG. By immunoelectrophoresis and ELISA this antibody reacts specifically with kappa light chains common to all human immunoglobulins. No antibody was detected against lambda light chain or non-immunoglobulin serum proteins. This antibody may cross react with kappa light chain from other species.
Western Blot 1:1,000 - 1:30,000
Immunohistochemistry 1:200 - 1:2,000
Immunocytochemistry 1:200 - 1:2,000
ELISA 1:1,000 - 1:30,000; for coating plates 1:100 - 1:500

hIg kappa Goat anti-Human Ig kappaBethyl Laboratories A80-115P-43 1:150000
https://www.fortislife.com/products/secondary-antibodies/goat-anti-human-kappa-light-chain-antibody-hrp-conjugated/A80-115P
Target:Human Kappa Light Chain
Reactivity:Human
Application:ELISA, ICC, IHC, WB
Host:Goat
Clonality:Polyclonal
Format:Whole IgG
IsoType:IgG
Conjugate:HRP
Purity:Antigen Affinity Purified
The antibody was isolated by affinity chromatography using antigen coupled to agarose beads and conjugated to horseradish peroxidase (HRP). Antibody concentration was determined by extinction coefficient prior to conjugation: absorbance at 280 nm of 1.4 equals 1.0 mg of IgG. Molar enzyme/antibody protein ratio is 4:1. By immunoelectrophoresis and ELISA this antibody reacts specifically with kappa light chains common to all human immunoglobulins. No antibody was detected against lambda or nonimmunoglobulin serum proteins. This antibody may cross react with kappa light chain from other species.

Western Blot 1:2,000 - 1:20,000
Immunohistochemistry 1:200 - 1:2,000
Immunocytochemistry 1:200 - 1:2,000
ELISA 1:10,000 - 1:100,000

hIg alpha Goat anti-Human IgA Bethyl Laboratories A80-102A-6 1:100
https://www.fortislife.com/products/immunoglobulins/goat-anti-human-iga-antibody/A80-102A
Target:Human IgA
Reactivity:Human
Application:ELISA, ICC, IHC, WB
Host:Goat
Clonality:Polyclonal
Format:Whole IgG
IsoType:IgG
Purity:Antigen Affinity Purified
Antiserum was solid phase adsorbed to ensure class specificity. The antibody was isolated by affinity chromatography using antigen coupled to agarose beads. Antibody concentration was determined by extinction coefficient: absorbance at 280 nm of 1.4 equals 1.0 mg of IgG. By immunoelectrophoresis and ELISA this antibody reacts specifically with human IgA. Cross reactivity with IgG, IgM and light chains is less than 0.1%. This antibody may cross react with IgA from other species.
Western Blot 1:1,000 - 1:30,000
Immunohistochemistry 1:200 - 1:2,000
Immunocytochemistry 1:200 - 1:2,000
ELISA 1:1,000 - 1:30,000; for coating plates 1:100 - 1:500

hIg alpha Goat anti-Human IgA Bethyl Laboratories A80-102P-26 1:75000
https://www.fortislife.com/products/secondary-antibodies/goat-anti-human-iga-antibody-hrp-conjugated/A80-102P
Target:Human IgA
Reactivity:Human
Application:ELISA, ICC, IHC, WB
Host:Goat
Clonality:Polyclonal
Format:Whole IgG
IsoType:IgG
Conjugate:HRP
Purity:Antigen Affinity Purified

Antiserum was solid phase adsorbed to ensure class specificity. The antibody was isolated by affinity chromatography using antigen coupled to agarose beads and conjugated to horseradish peroxidase (HRP). Antibody concentration was determined by extinction coefficient prior to conjugation: absorbance at 280 nm of 1.4 equals 1.0 mg of IgG. Molar enzyme/antibody protein ratio is 4:1. By immunoelectrophoresis and ELISA this antibody reacts specifically with human IgA. Cross reactivity with IgG, IgM and light chains is less than 0.1%. This antibody may cross react with IgA from other species.
Western Blot 1:2,000 - 1:20,000
Immunohistochemistry 1:200 - 1:2,000
Immunocytochemistry 1:200 - 1:2,000
ELISA 1:10,000 - 1:100,000

hIg epsilon Goat anti-Human IgE Bethyl Laboratories A80-108A-15 1:100
https://www.fortislife.com/products/immunoglobulins/goat-anti-human-ige-antibody/A80-108A
Target:Human IgE
Reactivity:Human
Application:ELISA, ICC, IHC, WB
Host:Goat
Clonality:Polyclonal
Format:Whole IgG
IsoType:IgG
Purity:Antigen Affinity Purified
Antiserum was solid phase adsorbed to ensure class specificity. The antibody was isolated by affinity chromatography using antigen coupled to agarose beads. Antibody concentration was determined by extinction coefficient: absorbance at 280 nm of 1.4 equals 1.0 mg of IgG. By immunoelectrophoresis and ELISA this antibody reacts specifically with human IgE. Cross reactivity with other human immunoglobulins and light chains is less than 1%.
Western Blot 1:1,000 - 1:30,000
Immunohistochemistry 1:200 - 1:2,000
Immunocytochemistry 1:200 - 1:2,000
ELISA 1:1,000 - 1:30,000; for coating plates 1:100 - 1:500

hIg epsilon Goat anti-Human IgE Bethyl Laboratories A80-108P-35 1:75000
https://www.fortislife.com/products/secondary-antibodies/goat-anti-human-ige-antibody-hrp-conjugated/A80-108P
Target:Human IgE
Reactivity:Human
Application:ELISA, ICC, IHC, WB
Host:Goat
Clonality:Polyclonal
Format:Whole IgG
IsoType:IgG
Conjugate:HRP
Purity:Antigen Affinity Purified
Antiserum was solid phase adsorbed to ensure class specificity. The antibody was isolated by affinity chromatography using antigen coupled to agarose beads and conjugated to horseradish peroxidase (HRP). Antibody concentration was determined by extinction coefficient prior to conjugation: absorbance at 280 nm of 1.4 equals 1.0 mg of IgG. Molar enzyme/antibody protein ratio is 4:1. By immunoelectrophoresis and ELISA this antibody reacts specifically with human IgE. Cross reactivity with other human immunoglobulins and light chains is less than 1%.
Western Blot 1:2,000 - 1:20,000
Immunohistochemistry 1:200 - 1:2,000
Immunocytochemistry 1:200 - 1:2,000
ELISA 1:10,000 - 1:100,000

mIg mu Goat anti-Mouse IgM Bethyl Laboratories A90-101A-22 1:100
https://www.fortislife.com/products/secondary-antibodies/goat-anti-mouse-igm-antibody/A90-101A
Target:Mouse IgM
Reactivity:Mouse
Application:ELISA, ICC, IHC, WB
Host:Goat
Clonality:Polyclonal
Format:Whole IgG
IsoType:IgG
Purity:Antigen Affinity Purified
Antiserum was solid phase adsorbed to ensure class specificity. The antibody was isolated by affinity chromatography using antigen coupled to agarose beads. Antibody concentration was determined by extinction coefficient: absorbance at 280 nm of 1.4 equals 1.0 mg of IgG. By immunoelectrophoresis and ELISA this antibody reacts specifically with mouse IgM. Cross reactivity with IgG subclasses and IgA is negligible. This antibody may cross react with IgM from other species. Some hybridoma clones may express aberrant immunoglobulin-related peptides that are improperly recognized by this antibody.
Western Blot 1:1,000 - 1:30,000
Immunohistochemistry 1:200 - 1:2,000
Immunocytochemistry 1:200 - 1:2,000
ELISA 1:1,000 - 1:30,000; for coating plates 1:100 - 1:500

mIg mu Goat anti-Mouse IgM Bethyl Laboratories A90-101P-34 1:75000
https://www.fortislife.com/products/secondary-antibodies/goat-anti-mouse-igm-antibody-hrp-conjugated/A90-101P
Target:Mouse IgM
Reactivity:Mouse
Application:ELISA, ICC, IHC, WB
Host:Goat
Clonality:Polyclonal
Format:Whole IgG
IsoType:IgG
Conjugate:HRP
Purity:Antigen Affinity Purified
Antiserum was solid phase adsorbed to ensure class specificity. The antibody was isolated by affinity chromatography using antigen coupled to agarose beads and conjugated to horseradish peroxidase (HRP). Antibody concentration was determined by extinction coefficient prior to conjugation: absorbance at 280 nm of 1.4 equals 1.0 mg of IgG. Molar enzyme/antibody protein ratio is 4:1. By immunoelectrophoresis and ELISA this antibody reacts specifically with mouse IgM. Cross reactivity with IgG subclasses and IgA is negligible. This antibody may cross react with IgM from other species.
Western Blot 1:2,000 - 1:20,000
Immunohistochemistry 1:200 - 1:2,000
Immunocytochemistry 1:200 - 1:2,000
ELISA 1:10,000 - 1:100,000

mIg gamma Goat anti-Mouse IgG-Fc Bethyl Laboratories A90-131A-16 1:100
https://www.fortislife.com/products/secondary-antibodies/goat-anti-mouse-igg-fc-fragment-antibody/A90-131A
Target:Mouse IgG-Fc Fragment
Reactivity:Mouse
Application:ELISA, ICC, IHC, WB
Host:Goat
Clonality:Polyclonal
Format:Whole IgG
IsoType:IgG
Purity:Antigen Affinity Purified
Antiserum was solid phase adsorbed to ensure class specificity. The antibody was isolated by affinity chromatography using antigen coupled to agarose beads. Antibody concentration was determined by extinction coefficient: absorbance at 280 nm of 1.4 equals 1.0 mg of IgG. By immunoelectrophoresis and ELISA this antibody reacts specifically with mouse IgG. Cross reactivity with IgA and IgM is negligible. This antibody may cross react with IgG from other species.
Western Blot 1:1,000 - 1:30,000
Immunohistochemistry 1:200 - 1:2,000
Immunocytochemistry 1:200 - 1:2,000
ELISA 1:1,000 - 1:30,000; for coating plates 1:100 - 1:500

mIg gamma Goat anti-Mouse IgG-Fc Bethyl Laboratories A90-131P-38 1:100000
https://www.fortislife.com/products/secondary-antibodies/goat-anti-mouse-igg-fc-fragment-antibody-hrp-conjugated/A90-131P
Target:Mouse IgG-Fc Fragment
Reactivity:Mouse
Application:ELISA, ICC, IHC, WB
Host:Goat
Clonality:Polyclonal
Format:Whole IgG
IsoType:IgG
Conjugate:HRP
Purity:Antigen Affinity Purified
Antiserum was solid phase adsorbed to ensure class specificity. The antibody was isolated by affinity chromatography using antigen coupled to agarose beads and conjugated to horseradish peroxidase (HRP). Antibody concentration was determined by extinction coefficient prior to conjugation: absorbance at 280 nm of 1.4 equals 1.0 mg of IgG. Molar enzyme/antibody protein ratio is 4:1. By immunoelectrophoresis and ELISA this antibody reacts specifically with Mouse IgG-Fc Fragment. Cross reactivity with IgA and IgM is negligible. This antibody may cross react with IgG from other species.
Western Blot 1:5,000 - 1:50,000
Immunohistochemistry 1:200 - 1:5,000
Immunocytochemistry 1:200 - 1:5,000
ELISA 1:10,000 - 1:100,000

mIg kappa Goat anti-Mouse Ig kappa Bethyl Laboratories A90-119A-14 1:100
https://www.fortislife.com/products/secondary-antibodies/goat-anti-mouse-kappa-light-chain-antibody/A90-119A
Target:Mouse Kappa Light Chain
Reactivity:Mouse
Application:ELISA, ICC, IHC, WB
Host:Goat
Clonality:Polyclonal
Format:Whole IgG
IsoType:IgG

Purity:Antigen Affinity Purified
The antibody was isolated by affinity chromatography using antigen coupled to agarose beads. Antibody concentration was determined by extinction coefficient: absorbance at 280 nm of 1.4 equals 1.0 mg of IgG. By immunoelectrophoresis and ELISA this antibody reacts specifically with kappa light chain common to all mouse immunoglobulins. Less than 1% cross-reactivity was detected against lambda light chain and non-immunoglobulin serum proteins. This antibody may cross react with kappa from other species. Some hybridoma clones may express aberrant immunoglobulin-related peptides that are improperly recognized by this antibody.
Western Blot 1:1,000 - 1:30,000
Immunohistochemistry 1:200 - 1:2,000
Immunocytochemistry 1:200 - 1:2,000
ELISA 1:1,000 - 1:30,000; for coating plates 1:100 - 1:500

mIg kappa Goat anti-Mouse Ig kappa Bethyl Laboratories A90-119P 1:100000
https://www.fortislife.com/products/secondary-antibodies/goat-anti-mouse-kappa-light-chain-antibody-hrp-conjugated/A90-119P
Target:Mouse Kappa Light Chain
Reactivity:Mouse
Application:ELISA, ICC, IHC, WB
Host:Goat
Clonality:Polyclonal
Format:Whole IgG
IsoType:IgG
Conjugate:HRP
Purity:Antigen Affinity Purified
Antiserum was solid phase adsorbed to ensure class specificity. The antibody was isolated by affinity chromatography using antigen coupled to agarose beads and conjugated to horseradish peroxidase (HRP). Antibody concentration was determined by extinction coefficient prior to conjugation: absorbance at 280 nm of 1.4 equals 1.0 mg of IgG. Molar enzyme/antibody protein ratio is 4:1. By immunoelectrophoresis and ELISA this antibody reacts specifically with kappa light chain common to all mouse immunoglobulins. No antibody was detected against lambda or nonimmunoglobulin serum proteins. This antibody may cross react with kappa from other species. Some hybridoma clones may express aberrant immunoglobulin-related peptides that are improperly recognized by this antibody.
Western Blot 1:2,000 - 1:20,000
Immunohistochemistry 1:200 - 1:2,000
Immunocytochemistry 1:200 - 1:2,000
ELISA 1:10,000 - 1:100,000

mIg lambda Goat anti-Mouse Ig lambda Bethyl Laboratories A90-121A-12 1:100
https://www.fortislife.com/products/secondary-antibodies/goat-anti-mouse-lambda-light-chain-antibody/A90-121A
Target:Mouse Lambda Light Chain
Reactivity:Mouse
Application:ELISA, ICC, IHC, WB
Host:Goat
Clonality:Polyclonal
Format:Whole IgG
IsoType:IgG
Purity:Antigen Affinity Purified
The antibody was isolated by affinity chromatography using antigen coupled to agarose beads. Antibody concentration was determined by extinction coefficient: absorbance at 280 nm of 1.4 equals 1.0 mg of IgG. By immunoelectrophoresis and ELISA this antibody reacts specifically with lambda light chain common to all mouse immunoglobulins. No antibody was detected against kappa or nonimmunoglobulin serum proteins. This antibody may cross react with lambda from other species.
Western Blot 1:1,000 - 1:30,000
Immunohistochemistry 1:200 - 1:2,000
Immunocytochemistry 1:200 - 1:2,000
ELISA 1:1,000 - 1:30,000; for coating plates 1:100 - 1:500

mIg lambda Goat anti-Mouse Ig lambda Bethyl Laboratories A90-121P-18 1:100000
https://www.fortislife.com/products/secondary-antibodies/goat-anti-mouse-lambda-light-chain-antibody-hrp-conjugated/A90-121P
Target:Mouse Lambda Light Chain
Reactivity:Mouse
Application:ELISA, ICC, IHC, WB
Host:Goat
Clonality:Polyclonal
Format:Whole IgG
IsoType:IgG
Conjugate:HRP
Purity:Antigen Affinity Purified
Antiserum was solid phase adsorbed to ensure class specificity. The antibody was isolated by affinity chromatography using antigen coupled to agarose beads and conjugated to horseradish peroxidase (HRP). Antibody concentration was determined by extinction coefficient prior to conjugation: absorbance at 280 nm of 1.4 equals 1.0 mg of IgG. Molar enzyme/antibody protein ratio is 4:1. By immunoelectrophoresis and ELISA this antibody reacts specifically with lambda light chain common to all mouse immunoglobulins. No antibody was detected against kappa or non-immunoglobulin serum proteins. This antibody may cross react with lambda from other species.

Western Blot 1:2,000 - 1:20,000
Immunohistochemistry 1:200 - 1:2,000
Immunocytochemistry 1:200 - 1:2,000
ELISA 1:10,000 - 1:100,000

Goat Anti-Human IgG H&L (HRP) preadsorbed Abcam Cat#97175 1:400
https://www.abcam.co.jp/goat-human-igg-hl-hrp-preadsorbed-ab97175.html
Goat Anti-Human IgG H&L (HRP) preadsorbed
Conjugation: HRP
Host species: Goat
Isotype: IgG polyclonal
Suitable for: ICC, IHC-P, ELISA, WB
By immunoelectrophoresis and ELISA this antibody reacts specifically with Human IgG and with light chains common to other Human immunoglobulins. No antibody was detected against non immunoglobulin serum proteins. Reduced cross-reactivity to bovine, chicken, goat, horse, mouse, pig, rabbit and rat was detected.
Antiserum was cross adsorbed using bovine, chicken, horse, mouse, pig, rabbit and rat immunosorbents to remove cross reactive antibodies. This antibody was isolated by affinity chromatography using antigen coupled to agarose beads and conjugated to Horse Radish Peroxidase (HRP).

Eukaryotic cell lines

Policy information about cell lines

Cell line source(s)	CHO/HGPRT (JCRB bank: JCRB0218), CHO K1 (Riken BRC: RCB0285), DT40 (Riken BRC: RCB1464), TT2F (provided by RIKEN BDR), P3X63Ag8.653 (ATCC: CRL-1580) and HCT116 (ATCC: CCL-247).
Authentication	No authentication was performed for commonly used cell lines. For microcell hybrid cell lines obtained in this study, all cell lines were authenticated by morphology, PCR assays with species-specific primers and karyotyping.
Mycoplasma contamination	All cell lines were tested negative for mycoplasma contamination.
Commonly misidentified lines (See ICLAC register)	No commonly misidentified cell lines were used in this study.

Palaeontology and Archaeology

Specimen provenance	*Provide provenance information for specimens and describe permits that were obtained for the work (including the name of the issuing authority, the date of issue, and any identifying information).*
Specimen deposition	*Indicate where the specimens have been deposited to permit free access by other researchers.*
Dating methods	*If new dates are provided, describe how they were obtained (e.g. collection, storage, sample pretreatment and measurement), where they were obtained (i.e. lab name), the calibration program and the protocol for quality assurance OR state that no new dates are provided.*

☐ Tick this box to confirm that the raw and calibrated dates are available in the paper or in Supplementary Information.

Ethics oversight	*Identify the organization(s) that approved or provided guidance on the study protocol, OR state that no ethical approval or guidance was required and explain why not.*

Note that full information on the approval of the study protocol must also be provided in the manuscript.

Animals and other organisms

Policy information about studies involving animals; ARRIVE guidelines recommended for reporting animal research

Laboratory animals	Jcl:ICR mice (RRID: IMSR_JCL:JCL:mOT-0001, CLEA, Tokyo, Japan) male and female, Age varied in each experiment as described in the text.<br>Crl:CD1(ICR) mice (Charles River, Kanagawa, Japan) male and female, 9 to 20 weeks old.<br>BALB/cAJcl mice (RRID: IMSR_JCL:JCL:mIN-0005, CLEA, Tokyo, Japan) female, 6 weeks old.
Wild animals	This study did not involve wild animals.
Field-collected samples	This study did not involve field-collected samples.
Ethics oversight	All procedures related to animal care and treatment were approved by each local University/Institutional Animal Care and Use Committee (Permit Number: 14-Y-23, 15-Y-31, 16-Y-20, 17-Y-28, 19-Y-22, 20-Y-13, 20-Y-31, 21-Y-26).

Note that full information on the approval of the study protocol must also be provided in the manuscript.

Human research participants

Policy information about studies involving human research participants

Population characteristics	*Describe the covariate-relevant population characteristics of the human research participants (e.g. age, gender, genotypic information, past and current diagnosis and treatment categories). If you filled out the behavioural & social sciences study design questions and have nothing to add here, write "See above."*
Recruitment	*Describe how participants were recruited. Outline any potential self-selection bias or other biases that may be present and how these are likely to impact results.*
Ethics oversight	*Identify the organization(s) that approved the study protocol.*

Note that full information on the approval of the study protocol must also be provided in the manuscript.

Clinical data

Policy information about clinical studies

All manuscripts should comply with the ICMJEguidelines for publication of clinical research and a completedCONSORT checklist must be included with all submissions.

Clinical trial registration	*Provide the trial registration number from ClinicalTrials.gov or an equivalent agency.*
Study protocol	*Note where the full trial protocol can be accessed OR if not available, explain why.*
Data collection	*Describe the settings and locales of data collection, noting the time periods of recruitment and data collection.*
Outcomes	*Describe how you pre-defined primary and secondary outcome measures and how you assessed these measures.*

Dual use research of concern

Policy information about dual use research of concern

Hazards

Could the accidental, deliberate or reckless misuse of agents or technologies generated in the work, or the application of information presented in the manuscript, pose a threat to:

No	Yes	
☒	☐	Public health
☒	☐	National security
☒	☐	Crops and/or livestock
☒	☐	Ecosystems
☒	☐	Any other significant area

Experiments of concern

Does the work involve any of these experiments of concern:

No	Yes	
☒	☐	Demonstrate how to render a vaccine ineffective
☒	☐	Confer resistance to therapeutically useful antibiotics or antiviral agents
☒	☐	Enhance the virulence of a pathogen or render a nonpathogen virulent
☒	☐	Increase transmissibility of a pathogen
☒	☐	Alter the host range of a pathogen
☒	☐	Enable evasion of diagnostic/detection modalities
☒	☐	Enable the weaponization of a biological agent or toxin
☒	☐	Any other potentially harmful combination of experiments and agents

ChIP-seq

Data deposition

☐ Confirm that both raw and final processed data have been deposited in a public database such as GEO.

☐ Confirm that you have deposited or provided access to graph files (e.g. BED files) for the called peaks.

Data access links<br>*May remain private before publication.*	*For "Initial submission" or "Revised version" documents, provide reviewer access links. For your "Final submission" document, provide a link to the deposited data.*
Files in database submission	*Provide a list of all files available in the database submission.*
Genome browser session<br>(e.g. UCSC)	*Provide a link to an anonymized genome browser session for "Initial submission" and "Revised version" documents only, to enable peer review. Write "no longer applicable" for "Final submission" documents.*

Methodology

Replicates	*Describe the experimental replicates, specifying number, type and replicate agreement.*
Sequencing depth	*Describe the sequencing depth for each experiment, providing the total number of reads, uniquely mapped reads, length of reads and whether they were paired- or single-end.*
Antibodies	*Describe the antibodies used for the ChIP-seq experiments; as applicable, provide supplier name, catalog number, clone name, and lot number.*
Peak calling parameters	*Specify the command line program and parameters used for read mapping and peak calling, including the ChIP, control and index files used.*
Data quality	*Describe the methods used to ensure data quality in full detail, including how many peaks are at FDR 5% and above 5-fold enrichment.*
Software	*Describe the software used to collect and analyze the ChIP-seq data. For custom code that has been deposited into a community repository, provide accession details.*

Flow Cytometry

Plots

Confirm that:

- [x] The axis labels state the marker and fluorochrome used (e.g. CD4-FITC).
- [x] The axis scales are clearly visible. Include numbers along axes only for bottom left plot of group (a 'group' is an analysis of identical markers).
- [x] All plots are contour plots with outliers or pseudocolor plots.
- [x] A numerical value for number of cells or percentage (with statistics) is provided.

Methodology

Sample preparation	Single-cell suspensions were prepared from the bone marrow, spleen, and PBMCs. All staining reactions with the appropriate antiboodies or isotype controls were incubated at 4°C for 30 min in 100 μl staining buffer (PBS with 5% FBS:BD Biosciences Brilliant stain buffer; 1:1).
Instrument	Acquisition was performed by Gallios and CytoFLEX S (Beckman Coulter).
Software	Acqusition and data analysis were performed with Cytexpert and Kaluza ver 2.1 (Beckman Coulter).
Cell population abundance	At least 20,000 cells per cells were recorded. Purity was determined by comarison with negative control.
Gating strategy	Single lymphocytes were determined by FSC-H/A as well as SSC-H/SSC-A. The boundaries between "positive" an d "negative" were dedfined by comparison with isotype controls.

- [x] Tick this box to confirm that a figure exemplifying the gating strategy is provided in the Supplementary Information.

Magnetic resonance imaging

Experimental design

Design type	*Indicate task or resting state; event-related or block design.*
Design specifications	*Specify the number of blocks, trials or experimental units per session and/or subject, and specify the length of each trial or block (if trials are blocked) and interval between trials.*
Behavioral performance measures	*State number and/or type of variables recorded (e.g. correct button press, response time) and what statistics were used to establish that the subjects were performing the task as expected (e.g. mean, range, and/or standard deviation across subjects).*

Acquisition

Imaging type(s)	*Specify: functional, structural, diffusion, perfusion.*
Field strength	*Specify in Tesla*
Sequence & imaging parameters	*Specify the pulse sequence type (gradient echo, spin echo, etc.), imaging type (EPI, spiral, etc.), field of view, matrix size, slice thickness, orientation and TE/TR/flip angle.*
Area of acquisition	*State whether a whole brain scan was used OR define the area of acquisition, describing how the region was determined.*

Diffusion MRI ☐ Used ☐ Not used

Preprocessing

Preprocessing software	*Provide detail on software version and revision number and on specific parameters (model/functions, brain extraction, segmentation, smoothing kernel size, etc.).*
Normalization	*If data were normalized/standardized, describe the approach(es): specify linear or non-linear and define image types used for transformation OR indicate that data were not normalized and explain rationale for lack of normalization.*
Normalization template	*Describe the template used for normalization/transformation, specifying subject space or group standardized space (e.g. original Talairach, MNI305, ICBM152) OR indicate that the data were not normalized.*
Noise and artifact removal	*Describe your procedure(s) for artifact and structured noise removal, specifying motion parameters, tissue signals and physiological signals (heart rate, respiration).*
Volume censoring	*Define your software and/or method and criteria for volume censoring, and state the extent of such censoring.*

Statistical modeling & inference

Model type and settings	*Specify type (mass univariate, multivariate, RSA, predictive, etc.) and describe essential details of the model at the first and second levels (e.g. fixed, random or mixed effects; drift or auto-correlation).*
Effect(s) tested	*Define precise effect in terms of the task or stimulus conditions instead of psychological concepts and indicate whether ANOVA or factorial designs were used.*

Specify type of analysis: ☐ Whole brain ☐ ROI-based ☐ Both

Statistic type for inference (See Eklund et al. 2016)	*Specify voxel-wise or cluster-wise and report all relevant parameters for cluster-wise methods.*
Correction	*Describe the type of correction and how it is obtained for multiple comparisons (e.g. FWE, FDR, permutation or Monte Carlo).*

Models & analysis

n/a	Involved in the study
☐	☐ Functional and/or effective connectivity
☐	☐ Graph analysis
☐	☐ Multivariate modeling or predictive analysis

Functional and/or effective connectivity	*Report the measures of dependence used and the model details (e.g. Pearson correlation, partial correlation, mutual information).*
Graph analysis	*Report the dependent variable and connectivity measure, specifying weighted graph or binarized graph, subject- or group-level, and the global and/or node summaries used (e.g. clustering coefficient, efficiency, etc.).*
Multivariate modeling and predictive analysis	*Specify independent variables, features extraction and dimension reduction, model, training and evaluation metrics.*
